# Pharmacokinetic evaluation of single-dose migalastat in non-Fabry disease subjects with ESRD receiving dialysis treatment, and use of modeling to select dose regimens in Fabry disease subjects with ESRD receiving dialysis treatment

**DOI:** 10.1371/journal.pone.0314030

**Published:** 2024-12-05

**Authors:** Franklin K. Johnson, Shirley Wu, Ginny Schmith, Hadis Williams, Jasmine Rutecki, Atef Halabi, Thorsten Feldkamp, Anthony Sileno

**Affiliations:** 1 Amicus Therapeutics, Inc., Princeton, New Jersey, United States of America; 2 Biogen, Inc., Cambridge, Massachusetts, United States of America; 3 Schmith PK/PD Consulting LLC, Cocoa Beach, Florida, United States of America; 4 CRS Clinical Research Services Kiel GmbH, Kiel, Germany; 5 Nephrology Centre Rendsburg-Eckernförde, Rendsburg, Germany; University of Verona: Universita degli Studi di Verona, ITALY

## Abstract

**Background:**

Fabry disease (FD) is an X-linked lysosomal disorder leading to multiorgan dysfunction, including renal impairment and the risk of significant accumulation for renally excreted drugs. Migalastat, an approved therapy in FD patients with amenable variants, is primarily eliminated in urine; however, its use had not been studied in patients with end-stage renal disease (ESRD) receiving dialysis therapy. This study investigated the pharmacokinetics (PK), dialyzability, and tolerability of 123 mg migalastat in non-FD subjects with ESRD on stable hemodialysis/hemodiafiltration (EudraCT 2018-003684-57). Results were analyzed by population PK and physiologically based PK (PBPK) modeling and intended to propose dose regimens resulting in negligible migalastat trough levels in plasma and comparable concentrations above the threshold in target tissues in FD patients with ESRD.

**Methods:**

Subjects with ESRD received 123 mg migalastat 24 hours before dialysis and, following an 8-day washout, immediately before dialysis. Matched controls with normal renal function (NRF) received migalastat 123 mg. Migalastat concentrations were measured in plasma, urine, and dialysate, and modeled to select regimens providing similar disposition to NRF.

**Results:**

Migalastat was extracted by hemodialysis/hemodiafiltration (74%/72%). PBPK modeling predicted that 123 mg every other week (QOW) with regular dialysis 2–3 times weekly in ESRD subjects produced: a fraction of time above EC_50_ similar to FD patients with NRF; adequate C_max_ for intracellular trafficking of mutant α-galactosidase A to the lysosome; and C_trough_ levels near the lower limit of quantification (LLOQ) similar to NRF subjects receiving 123 mg every other day. Migalastat 82 mg weekly produced a greater fraction of time above EC_50_ and longer duration of concentrations above the LLOQ, potentially resulting in accumulation in tissues.

**Conclusion:**

Migalastat was well extracted by hemodialysis/hemodiafiltration. Migalastat 123 mg QOW is the proposed dose regimen for further evaluation in FD patients with ESRD, which could inform expansion of treatment options.

**Trial registration:**

Trial registration: EU Clinical Trials Register, EudraCT number 2018-003684-57.

## Introduction

Fabry disease (FD) is a progressive, X-linked lysosomal disorder caused by deficient or absent lysosomal α-galactosidase A (α-Gal A) activity due to variants in the *GLA* gene [1]. The resulting lack of catalysis of the glycosphingolipid, globotriaosylceramide (GL-3), leads to accumulation, dysfunction, and inflammation in the lysosome and progressive deposition in the cardiovascular-renal system and other tissues [1,2]. In particular, the progressive accumulation of GL-3 in podocytes and other specialized tissues of the kidney leads to gradual deterioration of renal function [1,2]. End-stage renal disease (ESRD), severe cardiovascular, and cerebrovascular complications may cause significant morbidity and can be life-threatening and result in premature death in both males and females [1,3].

Migalastat is an analog of the terminal galactose of GL-3, which acts as a reversible, competitive inhibitor of α-Gal A [4,5]. It is a low molecular weight, orally administered iminosugar that serves as a pharmacological chaperone, allowing trafficking of mutated abnormal folded α-Gal A from the endoplasmic reticulum into the lysosome where it can exert its action [5,6]. In the lysosome, at a lower pH and at a higher concentration of relevant substrates, migalastat dissociates from α-Gal A allowing it to break down GL-3 [5,6]. Migalastat is an approved therapy for FD in patients with amenable *GLA* variants as determined by the GLP HEK migalastat amenability assay [3,5–7]. Approximately 77% of the migalastat dose is excreted in urine [5,6].

Since FD causes deterioration of renal function, consideration for dose adjustment to treat patients with FD who are in ESRD receiving dialysis therapy could be important data to drive informed treatment decisions. A recent review and meta-analysis of FD in patients with chronic kidney disease revealed the overall prevalence to be low (0.10% among dialysis patients with kidney transplantation and 0.17% among those without kidney replacement therapy) [8]. However, timely and effective treatment interventions, including disease-specific therapy, are needed for this FD population [8]. It is important to understand whether migalastat could be sufficiently dialyzed in patients with ESRD to achieve peak and trough levels similar to those observed in individuals with normal renal function (NRF). Widening treatment options in patients with FD and severe renal deterioration has the potential to improve clinical care of these affected patients and provide greater treatment choice.

This pharmacokinetic (PK) investigation was conducted to characterize the PK characteristics and confirm the dialyzability of migalastat in non-FD subjects with ESRD who were receiving hemodialysis (HD) or hemodiafiltration (HDF) and to assess the safety and tolerability of migalastat as part of the ongoing clinical development program. The PK study was followed by an investigation using population pharmacokinetics (popPK) and physiologically based PK (PBPK) modeling to determine the dosing regimen of migalastat for the treatment of FD in subjects with ESRD on HD or HDF. The proposed dose regimen would provide peak and trough plasma migalastat concentrations comparable to those in subjects with NRF, and therefore, demonstrate adequate drug clearance between doses.

## Materials and methods

### Ethics statement

The clinical study protocol and any substantial amendments were reviewed by an Independent Ethics Committee (IEC) and the responsible regulatory authority. Information provided to the subjects and recruitment advertisements (if applicable) were also reviewed by the IEC. The positive vote of the IEC (Ethikkommission bei der Ärztekammer Schleswig-Holstein, Bad Segeberg, Germany; approval date March 29, 2019; reference 024/19 P-EK) and the approval of the regulatory authority were obtained prior to the start of the study.

The study was performed in accordance with the study protocol and with Good Clinical Practice and applicable local laws and regulations, and the ethical principles have their origin in the current accepted version of the Declaration of Helsinki.

Written informed consent was obtained from subjects prior to participation in the study and prior to completion of any study-related procedure.

### Pharmacokinetic investigation

#### Study design

**Fig 1:**
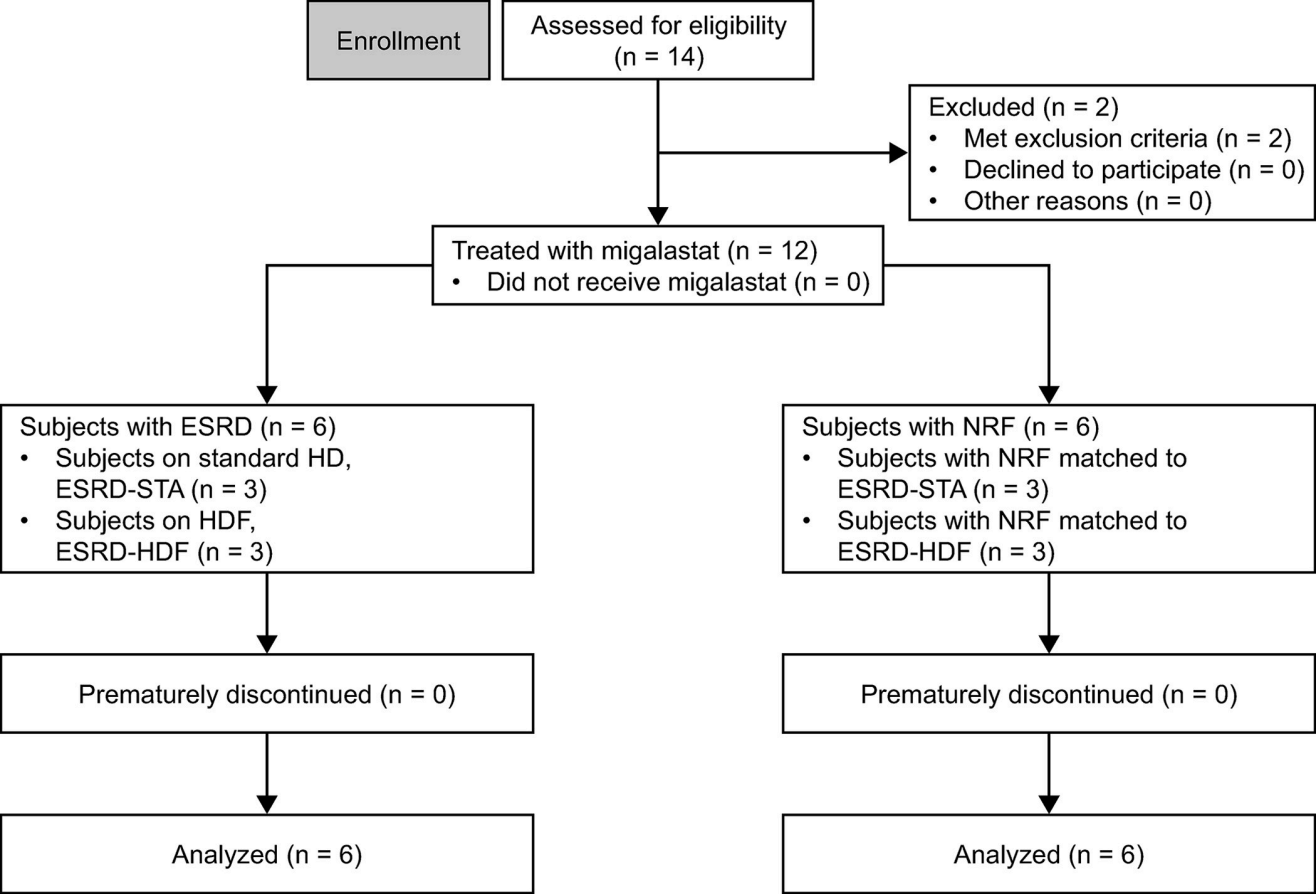
Flow diagram of subject disposition. ESRD, end-stage renal disease; HDF, hemodiafiltration; NRF, normal renal function; STA, standard hemodialysis.

This was a Phase I, open-label, non-randomized study involving six non-FD subjects with ESRD on HD (standard HD and HDF, n = 3 each) and six matched controls with NRF (Fig 1), conducted at a single center (CRS Clinical Research Services Kiel GmbH, Kiel, Germany) from June 5, 2019 (first subject enrolled) to December 20, 2019 (last subject completed). Recruitment started with the subjects with ESRD. Matched control subjects with NRF were recruited in parallel but not treated at the same time; each subject with NRF was enrolled after the follow-up visit of the respective subject with ESRD. The planned sample size of six evaluable subjects per renal function group was judged, based on experience, to be adequate to obtain reliable results meeting the objectives of the study given that it was designed to describe the PK and confirm the dialyzability of migalastat in non-FD subjects with ESRD and not to evaluate statistical differences between ESRD and non-ESRD subjects. Further, based on studies submitted to the FDA, including six to eight subjects per renal function group (in this case ESRD) is considered adequate to obtain a sufficient amount of PK data to make decisions on whether enough drug is removed to consider dose adjustment in this population [9].

#### Study population

Inclusion criteria for ESRD subjects were as follows: estimated glomerular filtration rate according to the Modification of Diet in Renal Disease equation (eGFR_MDRD_) < 15 mL/min/1.73 m^2^; receiving HD or HDF for least 4 hours thrice a week; stable on dialysis regimen for least 2 months; 18 to 79 years of age; body mass index (BMI) within 18.0 to 35.0 kg/m^2^; systolic blood pressure ≥ 90 to ≤ 179 mmHg, diastolic blood pressure ≥ 55 to ≤ 100 mmHg measured after 5 minutes in the supine position; resting pulse rate ≥ 50 to ≤ 99 beats/min measured after 5 minutes in the supine position; electrocardiogram (ECG) without clinically significant abnormalities; no febrile or infectious illness for at least 7 days prior to the first administration of study drug; willing to provide written informed consent, authorization for use and disclosure of personal health information or research-related health information, or has a legally authorized representative who has given written informed consent; and, if of reproductive potential, both male and female subjects agree to use a medically accepted method of contraception during the study and for at least 30 days after the last dose of study drug.

Exclusion criteria for ESRD subjects included: excess xanthine consumption (> 5 cups of coffee or equivalent per day); more than moderate alcohol consumption (> 35 g of ethanol regularly per day or > 245 g regularly per week); any history of alcohol or drug abuse; more than moderate smoking (> 10 cigarettes per day); consumption of xanthine-containing food or beverages within 48 hours before first dosing; consumption of grapefruit-containing food and beverages within 1 week before first dosing; positive urine drug screen; positive alcohol breath test; use of any new medication within 4 weeks before dosing (or at least 10 times the respective elimination half-life, whichever is longer) except hormonal contraceptives, paracetamol, and those drugs the renally impaired subject is currently taking for treatment of renal and/or concomitant disease; any change of chronic medication < 14 days prior to first dosing; requirement for treatment with miglitol or miglustat; previous kidney transplantation; any history of allergy or sensitivity to migalastat or other iminosugars or excipients; any history of drug hypersensitivity, asthma, urticaria, or other severe allergic diathesis as well as current hay fever; any intercurrent illness or condition that may preclude the subject from fulfilling the protocol requirements or suggests to the investigator that the potential subject may have an unacceptable risk by participating in this study; any severe or unsuitable concomitant medical condition (cardiovascular, neurological, hepatic, metabolic, hematological, immunological, pulmonary, or gastrointestinal disorder); any clinically significant abnormal laboratory value(s) and clinically significant ECG findings not due to the underlying disease; positive test for human immunodeficiency virus (HIV) antibodies or HIV-1 p24-antigen; positive hepatitis B-virus surface antigen (HBsAg) test; positive anti-hepatitis C-virus antibodies (anti-HCV) test; treatment with an investigational drug within 30 days of study start; inability to comply with study requirements or deemed otherwise unsuitable for study entry in the opinion of the investigator; female subject is pregnant or breastfeeding; blood donation within 30 days before signing informed consent to this study.

Inclusion criteria for healthy subjects matched to the ESRD subjects for age ± 10 years, body weight ± 10 kg, and sex, included the following: eGFR_MDRD_ ≥ 80 mL/min/1.73 m^2^; 18 to 79 years of age; BMI within 18.0 to 35.0 kg/m^2^; systolic blood pressure ≥ 90 to ≤ 145 mmHg, diastolic blood pressure ≥ 55 to ≤ 89 mmHg measured after 5 minutes in the supine position; resting pulse rate ≥ 50 to ≤ 99 beats/min measured after 5 minutes in the supine position; ECG without clinically significant abnormalities; no febrile or infectious illness for at least 7 days prior to the first administration of study drug; in general good health as determined by medical and surgical history, physical examination, 12-lead ECG, vital signs, and clinical laboratory evaluations; willing to provide written informed consent and authorization for use and disclosure of personal health information or research-related health information; and, if of reproductive potential, both male and female subjects agree to use a medically accepted method of contraception during the study and for at least 30 days after the last dose of study drug.

Exclusion criteria for healthy subjects included: excess xanthine consumption (> 5 cups of coffee or equivalent per day); more than moderate alcohol consumption (> 35 g of ethanol regularly per day or > 245 g regularly per week); any history of alcohol or drug abuse; more than moderate smoking (> 10 cigarettes per day); consumption of xanthine-containing food or beverages within 48 hours before dosing; consumption of grapefruit-containing food and beverages within 1 week before dosing; positive urine drug screen; positive alcohol breath test; any use of medication within 4 weeks before dosing (or at least 10 times the respective elimination half-life, whichever is longer) except hormonal contraceptives and paracetamol; previous kidney transplantation; any history of allergy or sensitivity to migalastat or other iminosugars or excipients; any history of drug hypersensitivity, asthma, urticaria, or other severe allergic diathesis as well as current hay fever; any intercurrent illness or condition that may preclude the subject from fulfilling the protocol requirements or suggests to the investigator that the potential subject may have an unacceptable risk by participating in this study; any history of chronic or recurrent metabolic, renal, hepatic, pulmonary, gastrointestinal, neurological (particularly a history of epileptic seizures), endocrinological, immunological, psychiatric or cardiovascular disease, myopathies, and bleeding tendency; any active physical disease (acute or chronic); laboratory values outside the reference range that are of clinical relevance in the opinion of the investigator (e.g. suggesting an unknown disease and requiring further clinical evaluation assessed by the investigator) especially aspartate aminotransferase (AST), alanine aminotransferase (ALT), gamma glutamyl transpeptidase (gamma-GT); positive test for HIV antibodies or HIV-1 p24-antigen; positive HBsAg test; positive anti-HCV test; treatment with an investigational drug within 30 days of study start; inability to comply with study requirements or deemed otherwise unsuitable for study entry in the opinion of the investigator; female subject is pregnant or breastfeeding; blood donation within 30 days before signing informed consent to this study.

#### Standard hemodialysis and hemodiafiltration

In standard HD, solute removal is achieved across a semi-permeable membrane by diffusion [10]. Larger solutes are less easily removed because of their slower speed of diffusion [10,11]. In HDF, solute removal is achieved across a semi-permeable membrane by convection and diffusion, i.e., positive pressure is used to drive water across the membrane and solutes are dragged with water movement [10]. Larger solutes are removed, and a replacement fluid of high purity is infused into the blood line to replace fluid volume and electrolytes. In this way, HDF clears more molecules of a larger size compared with standard HD [10]. The majority of ESRD patients are treated with standard HD; however, a significant number are treated with HDF in European countries [12].

### Study treatment

**Fig 2:**
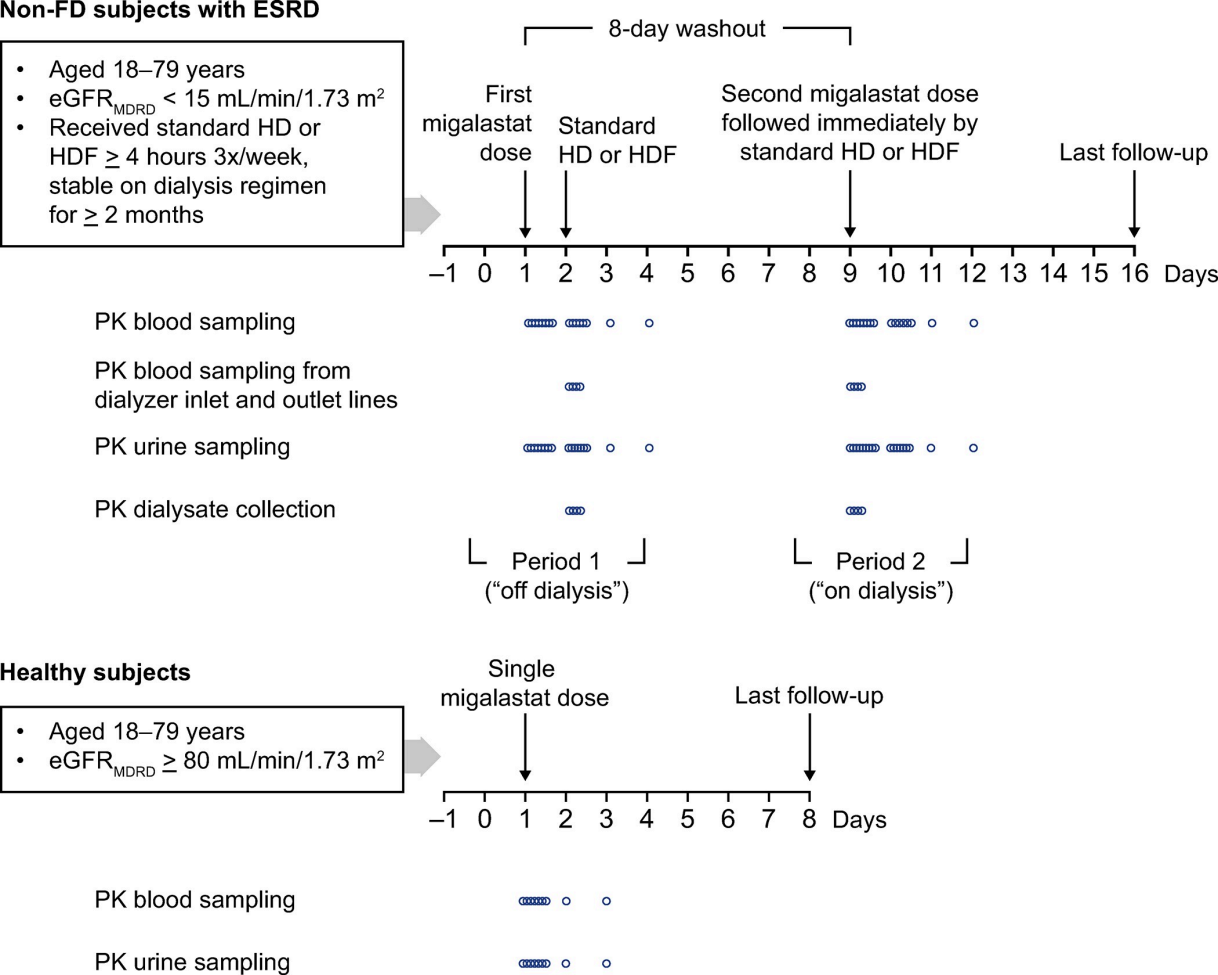
The timing of migalastat dosing and follow-up/sample collection. eGFR_MDRD_, estimated glomerular filtration rate according to the Modification of Diet in Renal Disease equation; ESRD, end-stage renal disease; FD, Fabry disease; HD, hemodialysis; HDF, hemodiafiltration; PK, pharmacokinetic.

All subjects were screened before the first treatment period (day −21 to −2) for eligibility to participate in the trial. On day −1, all qualified subjects were admitted to the clinical testing facility to begin treatment and study assessments. Subjects with ESRD on standard HD or HDF participated in two treatment periods, receiving the following treatments in a fixed sequence, with a washout phase of at least 8 days between dosing (Fig 2): period 1 (“off dialysis”) consisted of a single oral dose of 150 mg (equivalent to 123 mg in free base form) migalastat hydrochloride (HCl) 24 hours before the start of dialysis (i.e., in a dialysis-free interval); period 2 (“on dialysis”) consisted of a single oral dose of 123 mg migalastat immediately before the start of dialysis. Matched healthy control subjects with NRF participated in one treatment period and received a single oral dose of 123 mg migalastat.

### Pharmacokinetic sample collection

Subjects with ESRD on standard HD and HDF were domiciled at the clinical testing facility from the evening of day −1 (~8 pm) until discharge the morning of day 3. In the ESRD group for period 1 (dialysis initiated 24 hours post-dose), assessments included blood samples for plasma migalastat taken just prior to dosing (Time 0), and at 1, 2, 3, 4, 6, 8, 10, 12, 24, 48, and 72 hours post-dose, blood samples for plasma migalastat and dialysate samples for dialysate migalastat taken from the inlet line and outlet lines of the dialysis machine at 24:05, 25:00, 26:00, 27:00, 28:00, and 28:05 hh:mm post-dose. In the ESRD group for period 2 (dialysis initiated immediately post-dose), assessments included blood samples for plasma migalastat taken just prior to dosing (Time 0), at 5 minutes post-dose concurrent with start of dialysis, and at 1, 2, 3, 4, 4:05, 6, 8, 10, 12, 24, 48, and 72 hours post-dose, blood samples for plasma migalastat and dialysate samples for dialysate migalastat taken from the inlet line and outlet lines of the dialysis machine at 00:05, 01:00, 02:00, 03:00, 04:00, and 04:05 hh:mm post-dose. Urine samples for urine migalastat collected on days 1 to 4 at intervals of 0 to 24 hours, 24 to 48 hours, and 48 to 72 hours, in both periods. In the NRF group, subjects were domiciled at the clinical testing facility from the evening of day −1 (~8 pm) until discharge the morning of day 3. Assessments included blood samples for plasma migalastat taken just prior to dosing (Time 0), at 5 minutes post-dose, and at 1, 2, 3, 4, 6, 8, 10, 12, 24, and 48 hours post-dose, and urine samples for urine migalastat collected on days 1 and 2 at intervals of 0 to 24 hours and 24 to 48 hours. During the domiciled period, subjects with ESRD left the study center for dialysis. On day 4, these subjects returned to the study center for an ambulatory visit. Subjects in both groups had an ambulant follow-up visit 7 days after dosing.

### Analytical method

Following completion of the in-clinic portion of the study, samples were shipped to the bioanalytical laboratory for measurement of migalastat concentrations. Migalastat was analyzed using a validated liquid chromatography-tandem mass spectrometry method. The analytical range of the plasma migalastat assay was 5.88 to 2940 ng/mL. The urine migalastat assay comprised two analytical ranges: a low range from 10 to 500 μg/mL and a high range from 100 to 5000 ng/mL. The dialysate migalastat analytical range was 0.1 to 40 μg/mL. The bioanalytical study plan, review of the database, and bioanalytical report for plasma migalastat concentrations, migalastat concentrations in dialysate, and urine migalastat concentrations were successfully audited for compliance with US Federal Regulations by Quality Assurance teams.

### Noncompartmental analysis

Individual plasma migalastat concentration–time profiles were plotted on a linear and log-linear scale using Prism GraphPad, version 7.01. Noncompartmental PK analysis was performed on plasma, urine, and dialysate migalastat concentration–time data using Phoenix WinNonlin software, version 8.0.

Primary outcome measures for plasma migalastat PK parameters included: maximum plasma migalastat concentration (C_max_), time of maximum plasma migalastat concentration (t_max_), plasma migalastat at the last measurable time point (C_last_), plasma migalastat area under the curve (AUC) from Time 0 to the last measurable time point, t (AUC_0-t_), plasma migalastat AUC from Time 0 extrapolated to infinity (AUC_0-∞_), terminal elimination half-life (t_½_), and apparent oral clearance (CL/F) for ESRD and healthy subjects.

Pre- and post-dialyzer blood samples were also taken at limited selected timepoints from the inlet and outlet tubing of the dialysis machine for determination of the area under the concentration–time curve from venous samples entering the dialyzer (AUC_inlet_) and the area under the concentration–time curve from arterial samples leaving the dialyzer (AUC_outlet_), and mean plasma migalastat concentration during the dialysis interval (P). Pre- and post-dialyzer plasma migalastat AUCs were used to determine the dialysis extraction ratio (ED). Urine migalastat PK parameters were determined for ESRD subjects (if available) and healthy control subjects and included total amount of migalastat excreted in urine (A_e_), the fraction of the migalastat dose recovered in urine (F_e_), and renal clearance (CL_R_). Dialysate migalastat PK parameters were determined for ESRD subjects only and included dialysate clearance (CL_D_), migalastat concentration in dialysate (CD), dialyzer blood flow (QD), amount of migalastat recovered in dialysate (A_e_D), and fraction of the migalastat dose recovered in dialysate (F_e_D).

For descriptive statistics of plasma and dialysate migalastat concentrations, values below the lower limit of quantification (LLOQ) were set to missing. For calculation of the PK parameters, concentrations below LLOQ between two quantifiable concentrations and trailing concentrations below LLOQ were set to missing. Otherwise, missing data were not replaced or imputed in any way.

Safety assessments for the PK study (secondary outcome measures) included treatment-emergent adverse events (TEAEs), clinical safety laboratory test results, and ECG results, and vital signs were measured.

### Population pharmacokinetics analysis

The popPK analysis (model optimization) was performed using nonlinear mixed-effects modeling (NONMEM^®^) program version 7.4.4 (ICON Development Solutions, Ellicott City, Maryland, USA).

An earlier popPK model based on data from the original Phase III study in patients with FD served as the starting model (referred to in original publication as the Interim 3 Adult model); this model is described in detail in Leonowens C et al. 2022 [13]. This base model has a two-compartment structure with an absorption lag time and an absorption rate (Ka) that is related to time after dose up to 24 hours using a slope-intercept relationship. The effects of body weight (up to 70 kg) on PK parameters were described using allometric scaling, with an allometric exponent of 0.75 on CL/F and apparent intercompartmental clearance (Q/F), and allometric exponent of 1 on apparent volume of distribution of the central (V_2_/F) and peripheral (V_3_/F) compartments. Above 70 kg, there did not appear to be any correlation between body weight and CL/F, Q/F, V_2_/F and V_3_/F. Additionally, renal function affects CL/F and FD status affects both CL/F and V_2_/F. The effect of renal function on CL/F is characterized as a piecewise function in which CL/F is linearly dependent on estimated glomerular filtration rate (eGFR) until 120 mL/min/1.73 m^2^, above which the effect is a constant. FD status is characterized as a binary effect. Interindividual variability (IIV) is included on CL/F and V_2_/F with correlation and on the slope and intercept on Ka. Additionally, the equations used in the model are presented in S1 Table.

Several possible dialysis-related changes to the model were considered: increase in apparent plasma clearance (CL/F) due to apparent dialysis clearance (CL_D_) during active dialysis; volume changes due to dialysis and fluid accumulation between dialysis; and separate residual variability during dialysis. Model selection considered change in objection function value, goodness-of-fit plots, magnitude of interindividual and residual variabilities, precision of estimates, successful minimization, and physiologic rationale.

The selected model was used for Monte Carlo simulations of the exposures of FD patients with ESRD after various dosing regimens.

For the simulations, two sets of 100 virtual FD subjects were simulated. The set representing subjects with NRF simulated weight and eGFR as normal distribution based on the weight (72.5 ± 15.4 kg) and eGFR (87.8 ± 31.7 mL/min/1.73 m^2^) of FD patients in Study AT1001-011. For the set representing subjects with ESRD, the eGFR was changed to be simulated as a uniform distribution between 5 and 15 mL/min/1.73 m^2^. Dialysis clearance differs within and between subjects with no clear predictable pattern; therefore, dialysis clearance in the ESRD population was simulated as a uniform distribution assuming the range from the current study. Simulation scenarios evaluated twice- and thrice-weekly dialysis and a migalastat dose of 82 or 123 mg administered weekly (QW) or every other week (QOW).

Simulated concentration–time data were used to determine C_max_, concentration at the end of a dosing interval at steady state (C_trough_), and the average plasma concentration (C_avg_). C_avg_ was calculated as the AUC for a steady-state dosing interval (AUC_tau_) divided by the duration of the dosing interval. The PK parameters after each of these two dosing scenarios were compared with the standard migalastat dosing (123 mg every other day [QOD]) in subjects with NRF by calculating the geometric mean ratio (ESRD: NRF) and the 90% confidence interval. Migalastat exposures in each ESRD group and NRF subjects were considered bioequivalent if the 90% confidence intervals of the geometric mean ratio of C_max_ or AUC_tau_ were within the range of 0.8 to 1.25 (i.e., 80% to 125%).

### Physiologically based pharmacokinetic modeling investigation

PBPK modeling was conducted in PK-Sim Version 8 Build 22 (Bayer, Leverkusen, Germany). Data obtained during the PK study in non-FD subjects with ESRD receiving dialysis treatment and matched healthy subjects were combined with subjects from the Phase III study, AT1001-011, conducted in patients with FD and an eGFR ≥ 60 mL/min/1.73 m^2^. These combined data were used to update the previous PBPK model [14] to add components to describe dialysis to allow appropriate migalastat dose regimens for evaluation in a future clinical trial in FD patients with ESRD on dialysis treatment.

Previously, two PBPK models (full dataset and steady state) were developed that featured migalastat clearance from renal filtration, small hepatic metabolism, and minor extrahepatic glucuronidation [14]. The model was modified to evaluate overall metabolic elimination as an enzymatic process to allow for modeling of decreased clearance in ESRD subjects. Inputs defining the two models are summarized in S2 Table. Virtual ESRD subjects were created from subjects with NRF by fitting glomerular filtration rate (GFR) and theoretical hepatic enzyme expression level to the observed plasma concentration–time data of ESRD subjects in the current study. Dialysis was modeled by addition of a dialysis compartment, implementation of dialysis-dependent passive transport processes, and creation of dialysis events in the simulation. The PBPK models were used to simulate 82 and 123 mg migalastat administered QW or QOW and twice- or thrice-weekly dialysis treatment. In lymphoblasts and fibroblasts isolated from males with FD, 75 different missense mutant forms of the α-Gal A enzyme, one insertion, and one splice-site variant were identified. Of these, 49 missense mutant forms had half maximal effective concentration (EC_50_) values ranging from 820 nM to > 1 mM [15]. An approximate median EC_50_ of 1 μM was selected as a target for simulations of time above EC_50_ in ESRD subjects with FD. The predicted total (free + bound) tissue concentrations were evaluated for the time above the concentration producing 50% of maximum effect (EC_50_; 1 μM) during a dosing interval (F_EC50_) or time above EC_50_ over 2 weeks.

## Results

### Pharmacokinetic investigation

#### Subject demographics

In total, 12 subjects were enrolled and completed the study; six non-FD subjects with ESRD receiving dialysis (three on standard HD and three on HDF) and six controls with NRF matched for age, sex, and weight (Table 1). All 12 enrolled subjects were included in PK and safety analyses.

**Table 1:** Demographic characteristics of the enrolled subjects.

Parameter	Statistic	ESRD^a^ (n = 6)	NRF (n = 6)
**Age (years)**	Mean	47.5	48.0
	Median (min–max)	45.0 (37–64)	44.0 (33–69)
**Sex, n (%)**	Female^b^	4 (66.7)	4 (66.7)
	Male	2 (33.3)	2 (33.3)
**Race, n (%)**	White	6 (100)	6 (100)
**Weight (kg)**	Mean	72.7	70.3
	Median (min–max)	75.1 (56–83)	67.5 (60–82)
**BMI (kg/m** ^**2**^ **)**	Mean	26.4	26.1
	Median (min–max)	26.1 (20–31)	26.2 (21–34)
**eGFR (mL/min/1.73 m** ^**2**^ **)**	Mean	10.8	114.7
	Median (min–max)	8.0 (4–26)^c^	98.5 (89–172)^d^

BMI, body mass index; eGFR, estimated glomerular filtration rate; ESRD, end-stage renal disease; HD, hemodialysis; HDF, hemodiafiltration; NRF, normal renal function.

^a^Pooled ESRD group combined subjects on HD and subjects on HDF.

^b^Of the eight women included, five were of childbearing potential, two were at least 2 years postmenopausal, and one was surgically sterilized.

^c^This included one subject with an initial eGFR of 25.8 mL/min/1.73 m^2^; the value was repeated due to low serum creatinine and the updated eGFR was calculated as 12.2 mL/min/1.73 m^2^.

^d^This included one subject with a low serum creatinine, making the eGFR artifactually high.

### Individual plasma migalastat concentrations

**Fig 3:**
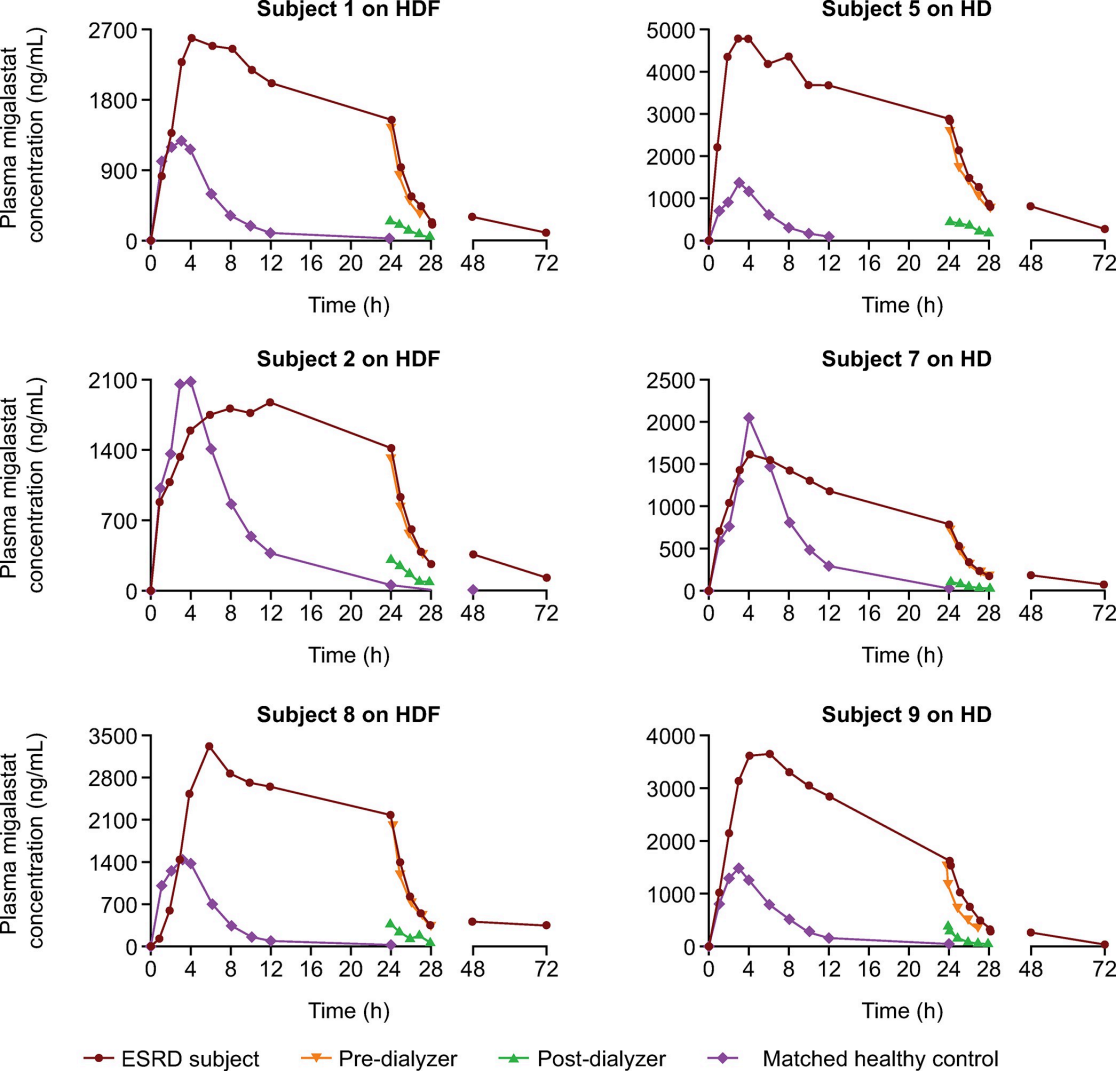
Individual plasma migalastat concentration–time curves for subjects with ESRD and matched controls during period 1 (“off dialysis”). ESRD, end-stage renal disease; HD, hemodialysis; HDF, hemodiafiltration. Period 1: Dose at 24 hours before dialysis.

**Fig 4:**
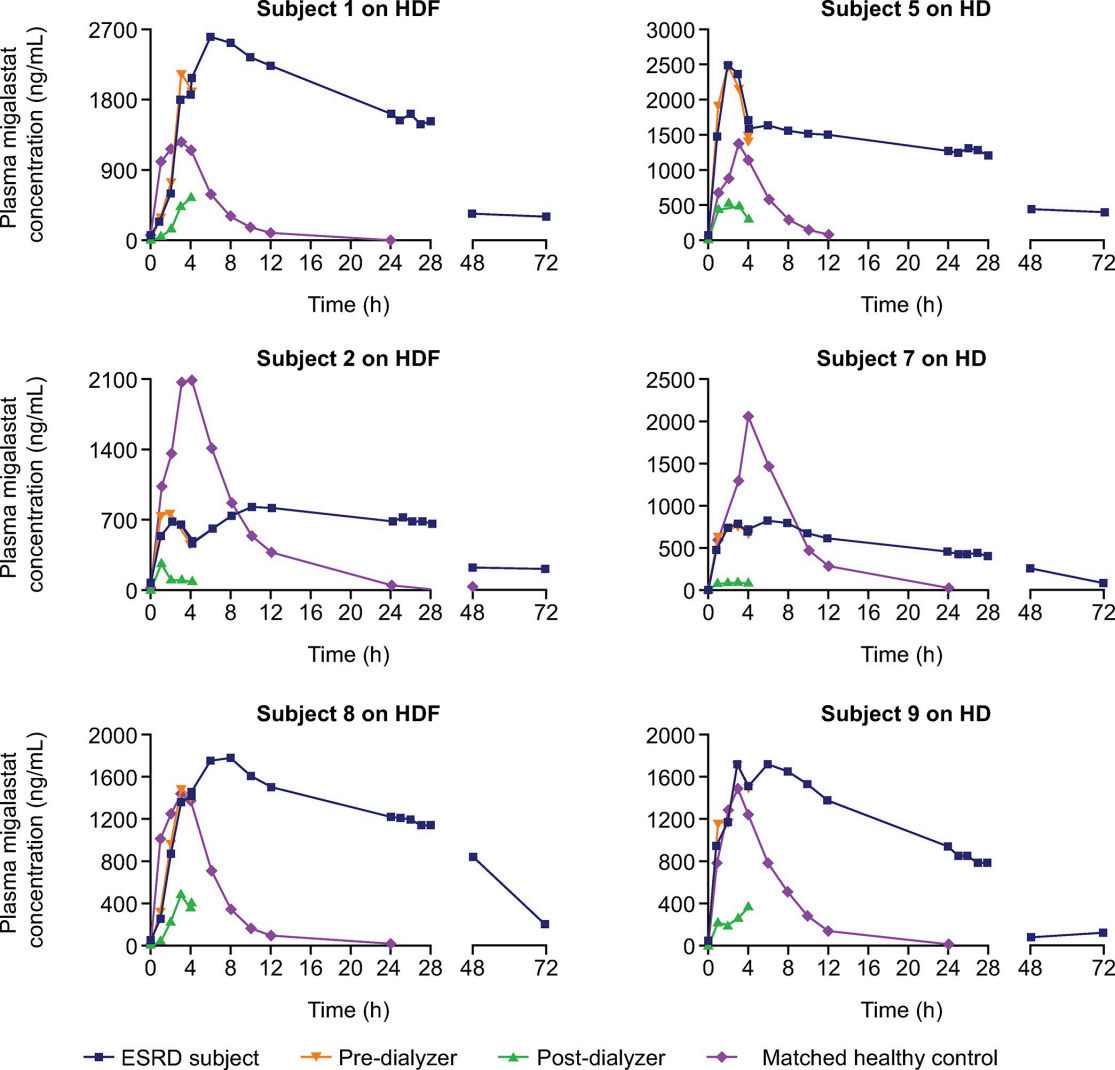
Individual plasma migalastat concentration–time curves for subjects with ESRD and matched controls during period 2 (“on dialysis”). ESRD, end-stage renal disease; HD, hemodialysis; HDF, hemodiafiltration. Period 2: Dose just prior to dialysis.

Individual plasma migalastat concentrations for ESRD subjects during periods 1 (“off dialysis”) and 2 (“on dialysis”) are shown in Figs 3 and 4, respectively, along with the concentration–time profiles of matched healthy controls.

All six subjects with ESRD had low but quantifiable migalastat concentrations above the LLOQ (5.88 ng/mL) at 72 hours in both periods. Quantifiable concentrations were negligible (< 5% of C_max_) pre-dose at period 2 (168 hours post-period 1 dosing) and did not impact period 2 PK assessments.

Generally, plasma migalastat concentration–time profiles appeared similar between subjects with ESRD receiving HD or HDF. Plasma concentrations peaked at approximately 5 to 6 hours post-dose in subjects with ESRD and at approximately 3 to 4 hours post-dose in matched controls with NRF. Plasma migalastat concentrations for each subject with ESRD during the “off-dialysis” period (Fig 3) were consistently higher than those during the “on-dialysis” period (Fig 4). Outside dialysis windows, plasma migalastat concentrations declined at a much slower rate in the subjects with ESRD than in matched controls with NRF. In all ESRD subjects, the concentration of plasma migalastat remained high until 24 to 28 hours post-dose (the 4-hour time interval of dialysis treatment) for subjects in the “off-dialysis” period, then declined precipitously to levels near the LLOQ. Post-dialyzer concentrations were substantially lower than the corresponding pre-dialyzer concentrations. Although both dosing-dialysis regimens removed a substantial amount of migalastat from plasma during the “on-dialysis” period, migalastat concentrations were appreciably greater than those of NRF subjects through 72 hours post-dose.

### Migalastat pharmacokinetics in plasma and dialysate

Overall, plasma migalastat exposure was substantially higher in subjects with ESRD (Table 2) than in matched controls with NRF regardless of timing of dialysis. The difference was more pronounced when comparing subjects with ESRD to matched controls with NRF when administration was during period 1 (“off dialysis”), with median area under the concentration–time curve from time 0 to 24 hours (AUC_0–24h_) approximately 5.9-fold but median C_max_ only 2.1-fold higher than in matched controls with NRF, respectively. During period 2 (“on dialysis”), the difference was somewhat lower, with median AUC_0–24h_ approximately 3.5-fold higher and median C_max_ only 1.2-fold higher than in matched controls with NRF. The median rate of absorption was prolonged by 1 to 5 hours in ESRD subjects relative to subjects with NRF. Median terminal half-life increased by 5- to 9-fold and, correspondingly, median apparent clearance decreased by approximately 5- to 12-fold in ESRD subjects relative to subjects with NRF.

**Table 2:** Summary of PK parameters of migalastat in plasma.

PK parameter	Subjects with ESRD (n = 6)				Subjects with NRF (n = 6)
	Period 1 (“off dialysis”)^a^		Period 2 (“on dialysis”)		
	ESRD-HD	ESRD-HDF	ESRD-HD	ESRD-HDF	
**C**_**max,24h**_ **(ng/mL)**					
**Median** **Range** **N**	3650 1610‒4770 3	2600 1870‒3320 3	1720 816‒2500 3	1770 816‒2620 3	1455 1260‒2070 6
**t**_**max**_ **(h)**					
**Median** **Range** **N**	4.0 3.0‒6.0 3	6.0 4.0‒12.0 3	3.0 2.0‒6.1 3	8.0 6.0‒10.0 3	3.0 3.0‒4.0 6
**AUC**_**0-24h**_ **(ng·h/mL)**					
**Median** **Range** **N**	61349 26959‒85764 3	46160 37909‒55216 3	31105 14602‒36337 3	32515 16594‒46401 3	9168 6915‒15926 6
**AUC**_**0-t**_ **(ng·h/mL)**					
**Median** **Range** **N**	74169 35210‒121553 3	59072 52914‒76048 3	45147 26672‒67965 3	68668 33308‒79321 3	9168 6698‒16568 6
**AUC**_**0-∞**_ **(ng·h/mL)**					
**Median** **Range** **N**	73068 48688‒97448 2	115051 – 1	47544 29118‒84727 3	74400 40661‒87626 3	9237 6919‒16628 6
**t**_**1/2**_ **(h)**					
**Median** **Range** **N**	17.3 15.4‒19.2 2	31.1 – 1	20.9 14.7‒29.2 3	19.6 18.7‒25.1 3	3.5 2.0‒5.9 6
**CL/F (L/h)**					
**Median** **Range** **N**	2.31 1.54‒3.08 2	1.30 – 1	3.15 1.77‒5.15 3	2.02 1.71‒3.69 3	16.3 9.02‒21.7 6
**V**_**z**_ **/F (L)**					
**Median** **Range** **n**	59.7 34.1‒85.3 2	58.4 – 1	74.6 66.9‒155.4 3	56.9 46.2‒133.6 3	73.5 54.9‒97.1 6

AUC_0–∞_, area under the concentration–time curve from time 0 to infinity; AUC_0–24h_, area under the concentration–time curve from time 0 to 24 hours; AUC_0–t_, area under the concentration–time curve from time 0 to the last measurable concentration; CL/F, apparent plasma clearance; C_max,24h_, maximum concentration observed between time 0 to 24 hours; ESRD, end-stage renal disease; HD, standard hemodialysis; HDF, hemodiafiltration; NRF, normal renal function; PK, pharmacokinetic; t_1/2_, apparent terminal elimination half-life; t_max_, time to maximum concentration; V_z_/F, apparent terminal phase volume of distribution.

^a^AUC_0–∞_ and other extrapolated parameters are not reported for three ESRD-HDF subjects because > 50% of area was extrapolated for these subjects.

A summary of migalastat PK parameters in dialysate and extraction from plasma is shown in Table 3. The median fraction of the dose recovered in dialysate was similar between the “off dialysis” (22.2%) and “on dialysis” (22.1%) treatment periods. Comparison of median fraction of the dose recovered in dialysate between dialysis methods, standard HD versus HDF, were generally similar also (standard HD, 21.2%; HDF, 22.6%). Overall, median CL_D_ was similar between treatment periods: 8.81 L/h for “off dialysis” and 11.1 L/h for “on dialysis”. Median CL_D_ was generally similar between dialysis methods (standard HD, 6.13 L/h; HDF, 10.2 L/h). Both methods of dialysis demonstrated significant decreases in plasma migalastat exposures during 4-hour dialysis treatments as measured by the ratio between AUC_inlet_ and AUC_outlet_. The ratio is expressed as percent of the absorbed dose extracted by dialysis. Overall, median percent of the absorbed dose extracted by dialysis was similar between treatment periods: 73.3% extracted during “off dialysis” and 76.3% during “on dialysis”. Median percent of the absorbed dose extracted by dialysis comparisons between dialysis methods were similar (standard HD, 74.1%; HDF, 71.8%).

**Table 3:** Summary of PK parameters of migalastat in dialysate and extraction from plasma by dialysis.

PK parameter	Subjects with ESRD (n = 6)			
	Period 1 (“off dialysis”)		Period 2 (“on dialysis”)	
	ESRD-HD	ESRD-HDF	ESRD-HD	ESRD-HDF
**F**_**e**_ **D (%)**				
** Median** **Range** **N**	21.8 6.37–25.3 3	22.6 12.5–27.9 3	32.8 17.9‒45.4 3	17.1 15.7‒26.3 3
**CL**_**D**_ **(L/h)**				
** Median** **Range** **N**	6.13 6.01‒10.8 3	10.2 7.39‒12.2 3	10.6 9.44‒11.5 3	11.9 6.60‒12.3 3
**AUC**_**inlet**_ **(ng·h/mL)**				
** Median** **Range** **N**	2859 1342‒5255 3	2465 2151‒3422 3	4756 2453‒7354 3	3456 2366‒4062 3
**AUC**_**outlet**_ **(ng·h/mL)**				
** Median** **Range** **N**	784 251‒1363 3	732 607‒890 3	924 376‒1694 3	993 576‒1005 3
**ED (%)**				
** Median** **Range** **N**	74.1 72.6‒81.3 3	71.8 70.3‒74.0 3	80.6 77.0‒84.7 3	75.6 70.9‒75.6 3

AUC_inlet_, area under the concentration–time curve from venous samples entering the dialyzer; AUC_outlet_, area under the concentration–time curve from arterial samples leaving the dialyzer; CL_D_, apparent dialysate clearance; ED, dialysis extraction ratio expressed as [(AUC_inlet_−AUC_outlet_)/AUC_inlet_] x100; ESRD, end-stage renal disease; F_e_D, fraction of the migalastat dose recovered in dialysate; HD, standard hemodialysis; HDF, hemodiafiltration; PK, pharmacokinetic.

A summary of migalastat PK parameters in urine is shown in S3 Table. Median fraction of the migalastat dose excreted in urine (F_e_%) and corresponding amount recovered in urine (A_e_) in healthy subjects with NRF was approximately 39.6% (A_e_, 59.4 mg) compared with 3.1% (A_e_, 4.59 mg) excreted during “off dialysis” and 1.2% (A_e_, 1.85 mg) excreted during “on dialysis” in subjects with ESRD. Median renal clearance (CL_r_) was considerably lower for subjects with ESRD than healthy subjects with NRF (0.068 and 0.034 L/h for “off” and “on dialysis”, respectively, in subjects with ESRD versus 5.49 L/h for healthy subjects with NRF).

### Safety

TEAEs are presented in S4 Table. Single oral doses of 123 mg migalastat were well tolerated in subjects with ESRD and matched controls with NRF. There were eight TEAEs in total, all of which were considered non-serious and mild to moderate in severity, with half of the subjects treated reporting at least one TEAE. The most common TEAE was headache, which was reported by four subjects. All TEAEs were transient and resolved by the end of the study. No subjects discontinued the study due to a TEAE.

No medically relevant effects on safety laboratory parameters, vital signs, or ECG parameters were observed.

### Population pharmacokinetics analysis

The popPK model was modified to incorporate dialysis clearance on apparent clearance during each dialysis session. Dialysis-related changes in volume or residual error were not incorporated because these changes did not improve the goodness-of-fit plots or residual variability. The parameter estimates of the final models are included in S1 Table.

**Fig 5:**
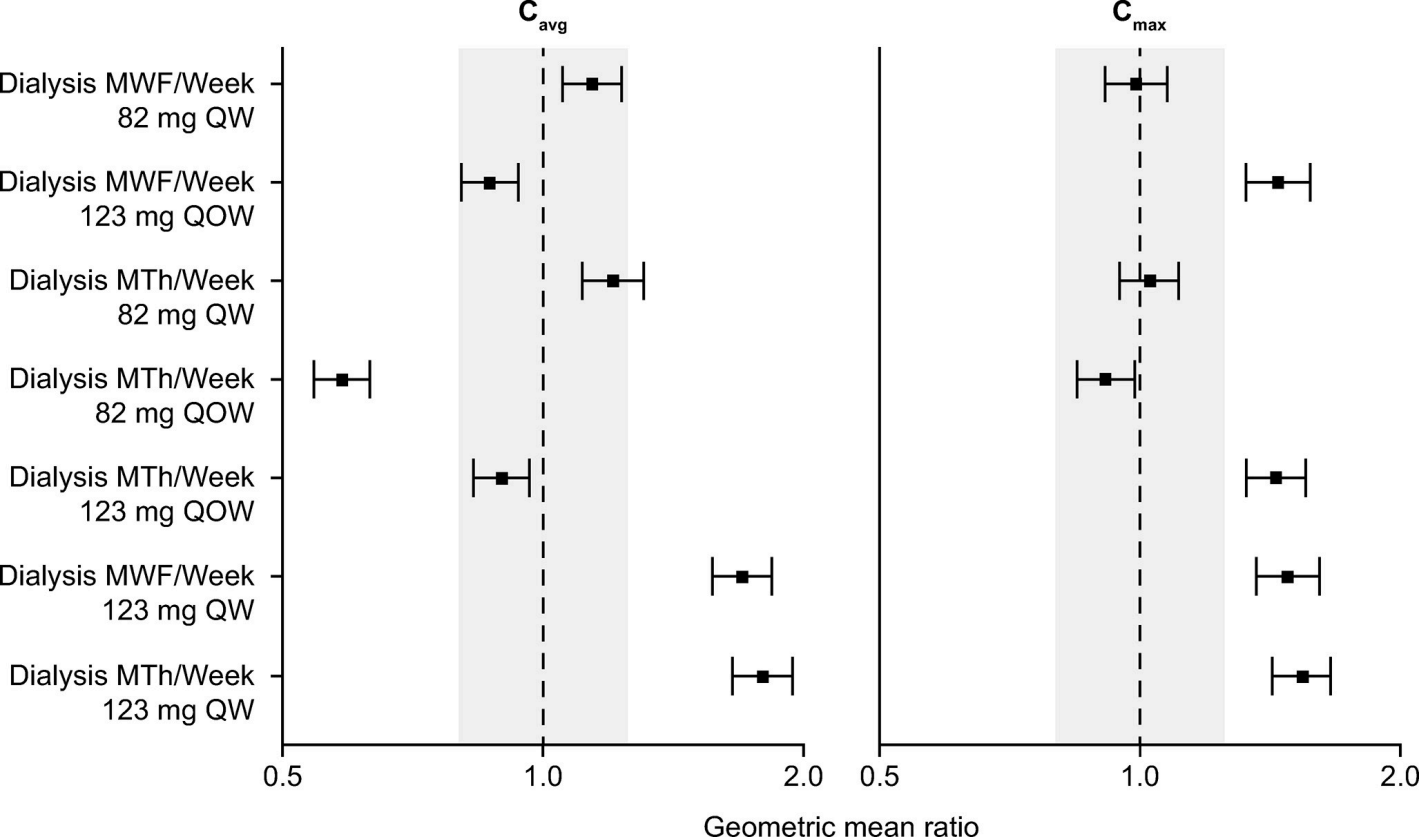
Geometric mean ratios for C_avg_ and C_max_ for week-based dosing and dialysis schedule scenarios. C_avg_, average concentration; C_max_, maximum concentration; MTh, Monday, Thursday; MWF, Monday, Wednesday, Friday; QOW, every other week; QW, every week. Black dashed line is the geometric mean ratio of 1.0; gray-shaded region is the bioequivalence criteria for 0.8 to 1.25 for the 90% confidence interval of the geometric mean ratio; dot and segment are the geometric mean ratio and 90% confidence interval.

**Fig 6:**
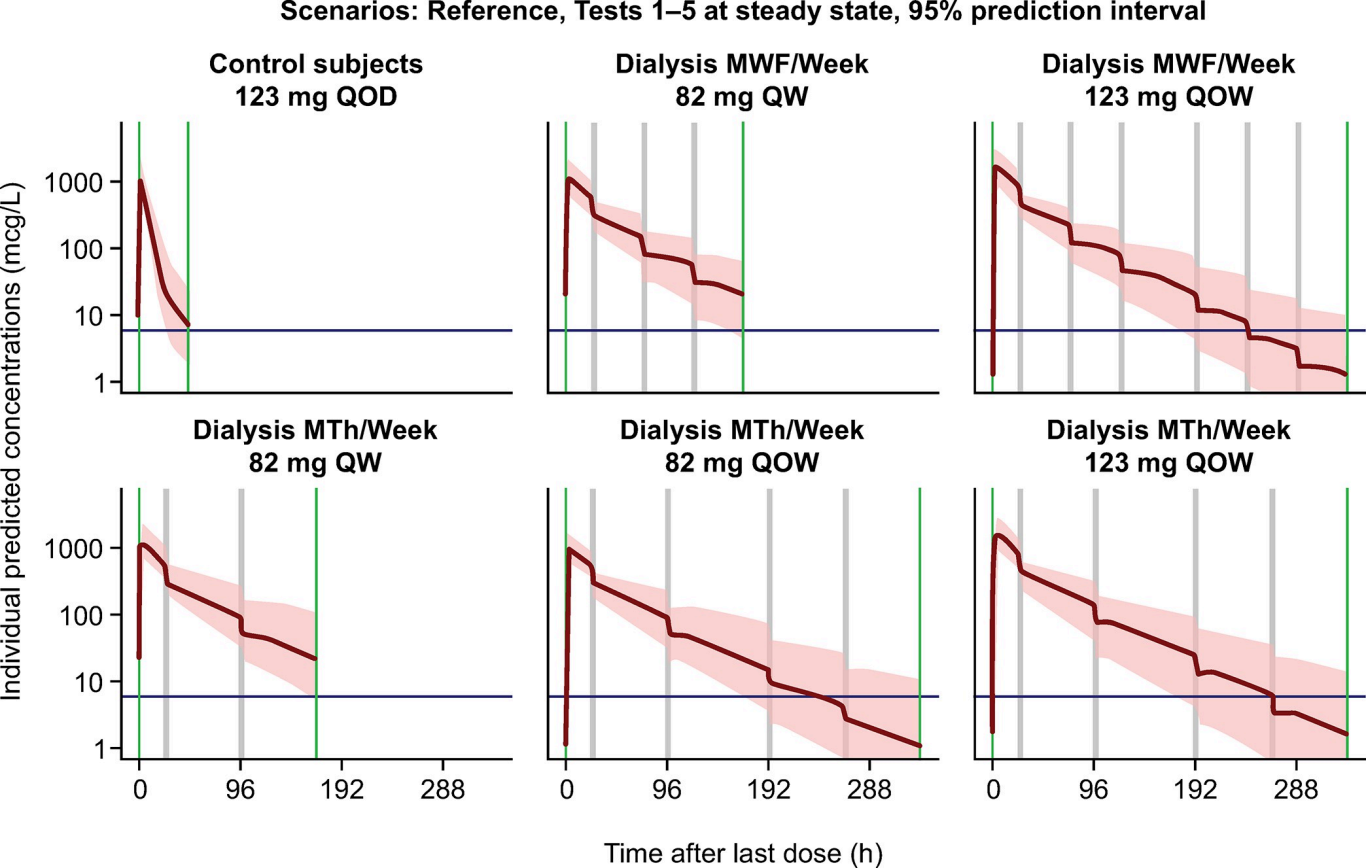
Median (95% prediction interval) steady-state concentration–time profiles for week-based dosing and dialysis schedule scenarios. LLOQ, lower limit of quantification; MTh, Monday, Thursday; MWF, Monday, Wednesday, Friday; QOD, every other day. Red area is the 2.5–97.5th prediction interval; red line is the median; blue line is the LLOQ; green line is the migalastat dose; gray area is the dialysis period.

**Fig 7:**
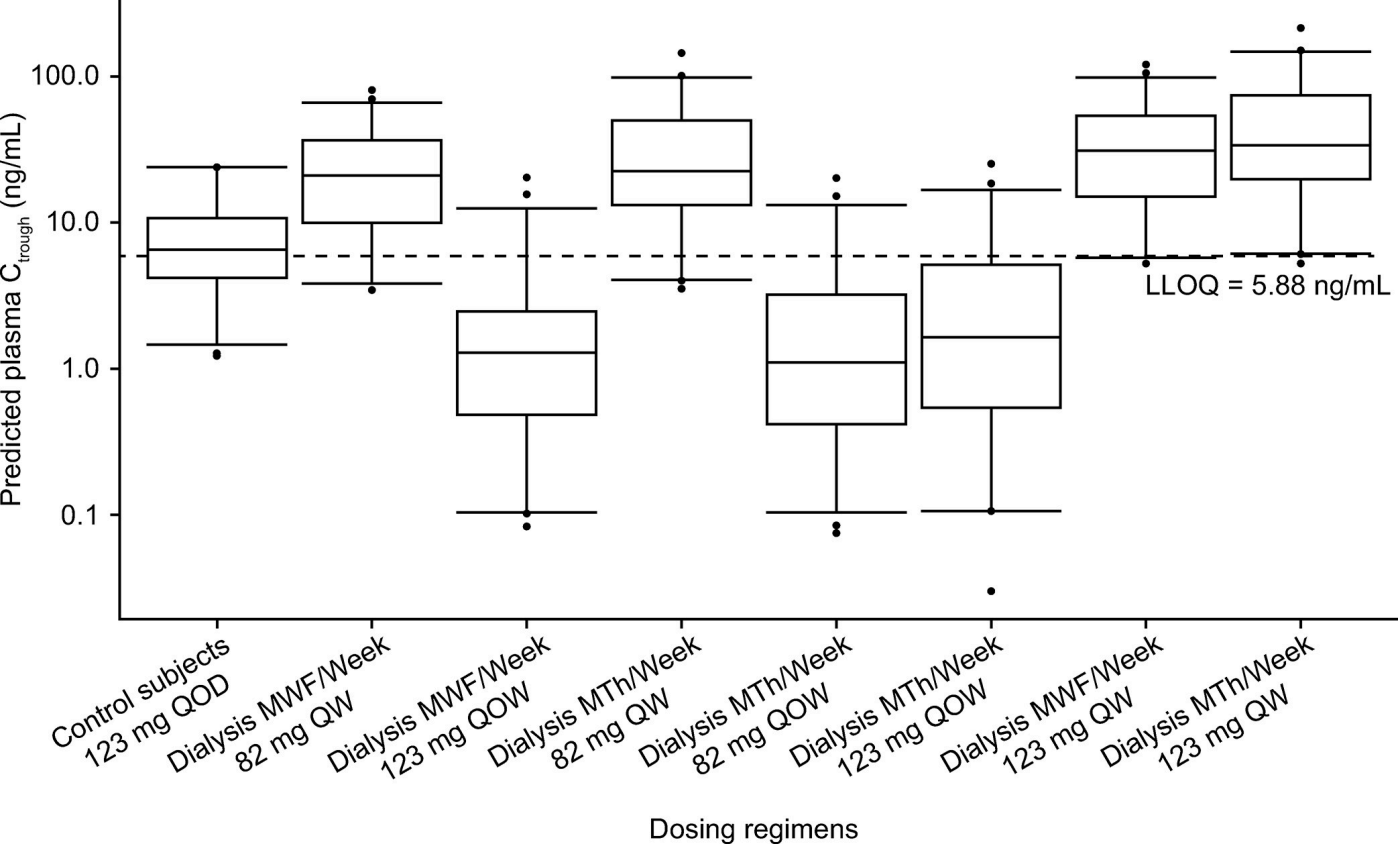
Predicted C_trough_ for week-based dosing and dialysis schedule scenarios. C_trough_, concentration at the end of a dosing interval at steady state; LLOQ, lower level of quantification; MTh, Monday, Thursday; MWF, Monday, Wednesday, Friday; QW, every week; QOW, every other week, QOD, every other day. The box plot represents the median, 25th, and 75th percentile predicted concentrations. The whisker ends represent the 2.5th and 97.5th percentile values. Dashed line is the LLOQ for migalastat detection (5.88 ng/mL).

PopPK simulations showed that:

- Migalastat 82 mg QW dosing along with twice- or thrice-weekly dialysis was predicted to result in migalastat C_avg_ and C_max_ values that are approximately bioequivalent to those of subjects with NRF receiving 123 mg of migalastat QOD (Figs 5 and 6). Few subjects attained C_max_ > 10 μM or achieved concentrations that were below the limit of quantification (BLQ) at the end of a dosing interval at steady state (Table 4), which are hypothesized to facilitate intracellular trafficking of mutant α-Gal A to the lysosome and dissociation of the migalastat and α-Gal A complex.
- Migalastat 123 mg QOW along with twice- or thrice-weekly dialysis was predicted to result in a C_avg_ that was bioequivalent to, but with a higher C_max_ than, subjects with NRF receiving 123 mg of migalastat QOD (Figs 5 and 6). Migalastat 123 mg QOW regimens resulted in a substantial proportion of the subjects who attained C_max_ > 10 μM and/or C_trough_ that was BLQ (Table 4, Fig 7).
- Migalastat 82 mg QOW resulted in a C_max_ and C_avg_ that was too low, while migalastat 123 mg QW resulted in a C_max_ and C_avg_ that were high, relative to subjects with NRF.

**Table 4:** Simulation scenarios for QW and QOW dosing and week-based dialysis schedules. Bioequivalence was determined by comparing with predictions in virtual subjects with NRF.

Scenario	Dialysis frequency	Migalastat regimen	Bioequivalent?		Subjects (%)	
			C_avg_	C_max_	C_max_ > 10 μM	C_trough_ BLQ
**Reference**	None	123 mg QOD	Reference	Scenario	17	44
**Test 1**	MWF QW	82 mg QW	Yes	Yes	15	8
**Test 2**	MWF QW	123 mg QOW	Yes	High	56	92
**Test 3**	MTh QW	82 mg QW	Slightly high	Yes	12	7
**Test 4**	MTh QW	82 mg QOW	Low	Yes	8	90
**Test 5**	MTh QW	123 mg QOW	Yes	High	49	78
**Test 6** ^a^	MWF QW	123 mg QW	High	High	53	2
**Test 7** ^a^	MTh QW	123 mg QW	High	High	58	1

BLQ, below the limit of quantification; C_avg_, average concentration; C_max_, maximum concentration; C_trough_, concentration at the end of a dosing interval at steady state; MWF, Monday, Wednesday, Friday; MTh, Monday, Thursday; NRF, normal renal function; QOW, every other week; QOD, every other day; QW, every week.

^a^These simulations were added after the twice QOW dosing simulations were conducted.

The simulations (along with all the simulation scenarios, which are detailed in S5 Table) suggested that migalastat 82 mg QW or migalastat 123 mg QOW could be acceptable doses in ESRD, but an understanding of tissue concentrations (hence, need for PBPK model described below) was needed to evaluate whether there is an advantage of migalastat 123 mg QOW when compared with migalastat 82 mg QW.

### Physiologically based pharmacokinetic analysis

The full dataset PBPK model and the steady-state PBPK model from the original PBPK model [14] were modified for use in subjects both with and without renal impairment. The extra-renal clearance (a minor elimination pathway) was changed from an overall hepatic clearance process to an enzyme metabolism process in the liver, such that individual clearance can be varied by controlling the expression level of the hepatic enzyme in the virtual subject. Based on popPK analysis, apparent clearance is proportional to renal clearance; therefore, a virtual subject with ESRD was created by decreasing the GFR to the median eGFR of the dialysis subjects in the study and decreasing the hepatic enzyme expression proportionally, fitting to the plasma concentration–time profiles of ESRD subjects before dialysis in the “off-dialysis” period. The dialysis process was modeled by adding a dialysis compartment in the PBPK model that filters migalastat for 4 hours when a dialysis event is triggered.

The PBPK models were used to predict migalastat tissue concentrations for the top two dosing regimens selected using popPK analysis. The predicted fractions of dosing interval with tissue concentration above EC_50_ are presented in Table 5 utilizing a male with NRF weighing 73 kg as a reference subject for comparison. Based on either model, the 123 mg QOW regimen in ESRD subjects produced a more similar fraction of time above EC_50_ to the reference case of subjects without renal impairment receiving 123 mg QOD, while migalastat 82 mg administered QW in ESRD subjects was associated with greater fraction of time above EC_50_ (Table 5). However, the 82 mg QW dose resulted in a longer duration of concentrations above the LLOQ and was consequently more vulnerable to accumulation. The migalastat 123 mg QOW dose predicted an adequate C_max_ with accompanying C_trough_ levels at or near the LLOQ (similar to NRF) after a minimum of four dialysis sessions.

**Table 5:** Predicted fraction of dosing interval above EC_50_ in the tissues for typical male subjects with weight 73 kg.

Dosing	Dialysis	Brain (%)	Heart (%)	Kidney (%)	Liver (%)	Skin (%)	Small intestine (%)
*Week-based dosing and dialysis schedule*							
**123 mg QOD in healthy subject**	None	0	5.00	40.1	14.5	19.9	25.2
**123 mg QOW in subject with ESRD**	2x/week	0	6.77	29.0	12.9	17.3	21.9
	3x/week	0	6.77	27.2	12.9	17.3	21.3
**82 mg QW in subject with ESRD**	2x/week	0	12.8	56.8	19.2	27.4	35.4
	3x/week	0	12.8	47.9	19.2	27.4	35.4
*Steady-state dataset model*							
**123 mg QOD in healthy subject**	None	0	7.71	35.0	11.0	–^a^	22.5
**123 mg QOW in subject with ESRD**	2x/week	0	7.59	41.5	11.5	–^a^	24.6
	3x/week	0	7.59	35.9	11.5	–^a^	21.4
**82 mg QW in subject with ESRD**	2x/week	0	13.8	67.0	15.8	–^a^	38.5
	3x/week	0	13.8	71.1	15.8	–^a^	38.5

EC_50_, concentration producing 50% of maximum effect; ESRD, end-stage renal disease; QOW, every other week; QOD, every other day; QW, every week.

^a^Skin tissue exposure was only observed in the single-dose mouse biodistribution study, so steady-state dataset model was not built to predict skin exposure.

## Discussion

This study investigated the PK, safety, and dialyzability of migalastat in non-FD subjects with ESRD undergoing standard HD and HDF. A popPK model and a PBPK model developed using these data allowed simulations to be conducted to determine the appropriate dose of migalastat in patients with ESRD that would result in exposures (in the plasma and the tissues) that would be appropriate, relative to the dose of migalastat in patients with FD who do not have ESRD. The findings and assumptions are discussed below.

Currently, migalastat is not recommended in patients with an eGFR < 30 mL/min/1.73 m^2^ [5,6]. However, as expected from its physical and chemical characteristics of being a highly soluble small molecule, results showed that migalastat was dialyzable with an extraction ratio of approximately 74 to 81% of the fraction of the dose in circulation after 24 hours post-dose. Additionally, deteriorated renal function resulted in only approximately < 5% of the dose recovered in urine over a 1-week period and about 20% recovered in dialysate, compared with 77% of the dose excreted in the urine from subjects with NRF [5,6]. Although one dialysis treatment contributed substantially to migalastat elimination, a significant fraction of the dose remains in plasma. While overall AUC increased considerably with decreasing renal function, the C_max_ increased to a smaller degree, likely because this PK parameter is related to absorption and volume of distribution and not clearance. The observed increased exposure in subjects with ESRD compared with the matched controls with NRF in this study is in line with results observed in a previous study (AT1001-015), where it was shown that migalastat plasma clearance decreased significantly in severe renal impairment, leading to significant prolongation of plasma terminal elimination half-life [3]. Therefore, dose adjustment, primarily by prolonged dosing intervals, will likely be required for FD patients with severe renal impairment with or without ESRD.

This study also confirmed there was no clinically relevant difference in PK, extraction ratio, and dialysate clearance between standard HD and HDF and that the 123 mg dose of migalastat was well tolerated, demonstrating a similar safety profile to that observed in other trials [16,17] in patients with FD who received 123 mg migalastat QOD (mild TEAEs such as headache were the most common, and no adverse events linked to migalastat led to the discontinuation of treatment). The lack of new safety signals was a further indication that dialysis was able to clear migalastat in subjects with ESRD. Based on similar dialysis extraction ratio values and recovery of the dose in dialysate with standard HD and HDF, subjects with ESRD could receive the same dose of migalastat regardless of the dialysis method.

In order to develop the best dosing regimen in FD patients with ESRD, one must understand the mechanism of action of migalastat. In FD patients with NRF, migalastat is administered QOD. The rationale for QOD dosing is that after migalastat binds to the α-Gal A active site, migalastat improves folding, stability, and lysosomal trafficking of numerous mutant forms, as well as wild-type, of α-Gal A [4,18–22]. If the variant is amenable, the enzyme is trafficked to the lysosome [18–20]. Migalastat quickly dissociates and clears from tissues within 24 to 48 hours post-dose, allowing the enzyme to initiate catalysis of GL-3 [3,23]. To allow for time intervals of intracellular trafficking, it is important to enable a dosing regimen of migalastat in ESRD that will lead a high C_max_ and C_avg_, but a trough level that is near BLQ to enable intracellular trafficking followed by catalysis of substrate. The study was designed to observe the effect of dosing migalastat 24 hours before dialysis relative to dialysis treatment being administered immediately following dosing so that the PK could be compared between timing of dialysis. As expected, plasma migalastat concentrations were considerably higher when dialysis was initiated 24 hours post-dose than when initiated immediately after dosing. Since the mechanism of action of migalastat is a concentration-driven process for intracellular trafficking, the study confirmed the timing of migalastat administration 24 hours before initiating dialysis.

Therefore, simulations were conducted using a popPK model with an adjusted dose of 82 mg QW or 123 mg QOW. The simulations derived from a popPK model showed that 82 mg QW or 123 mg QOW could theoretically be appropriate for patients with FD and ESRD. In order to differentiate these regimens and understand the tissue distribution, these two regimens were simulated based on an updated PBPK model. This PBPK model suggested that a migalastat dose of 123 mg QOW would be more appropriate than 82 mg QW in ESRD patients because this regimen balances the need for a high C_max_ above 10 μM (the concentration hypothesized to facilitate effective binding and trafficking for most α-Gal A mutants to the lysosome) with a C_trough_ that is BLQ (hypothesized to allow for migalastat dissociation from α-Gal A and systemic clearance), which is consistent with migalastat’s mechanism of action. In the ESRD group receiving 123 mg QOW, the time above EC_50_ in FD target tissues was similar to that of subjects with NRF receiving 123 mg QOD, indicating that this was the optimal regimen.

The fact that migalastat 123 mg QOW results in less than two-fold higher C_max_ than in subjects with NRF is not expected to lead to any safety issues given the known safety profile of migalastat. The dose suggested by the results of this PK study, along with popPK and PBPK modeling and simulations, is being studied in patients with FD and ESRD on HD or HDF in an ongoing clinical study (AT1001-025; NCT04020055) to determine potential effects of treatment of patients with FD with ESRD and amenable α-Gal A variants. Since migalastat is not currently recommended in patients with eGFR < 30 mL/min/1.73 m^2^, the results of this study could help inform the expansion of treatment options for patients with FD with severely impaired renal function who are receiving dialysis therapy.

One limitation of the study was the possibility of carry-over into period 2, when subjects initiated dialysis immediately after dosing. However, carry-over of migalastat concentrations did not impact the overall results of the study, particularly since all the data were included in the popPK and PBPK models (which can account for any changes in migalastat PK due to ESRD and dialysis).

## Conclusions

Migalastat was highly extracted by dialysis in non-FD subjects with ESRD, with no new safety signals detected in this study population. PopPK and PBPK predicted that migalastat 123 mg administered QOW would be optimal in FD patients with amenable variants and ESRD. This dose regimen is currently being studied in patients with FD and ESRD on HD or HDF (AT1001-025; NCT04020055). Since migalastat is not currently recommended in patients with eGFR < 30 mL/min/1.73 m^2^, the results of this study could help inform the expansion of treatment options for patients with FD with severely impaired renal function and for those who are receiving dialysis therapy.

## Supporting information

S1 Checklist TREND statement checklist. (PDF)

S1 Table PopPK model structure and parameter estimates. ALAG, absorption lag time; CL/F, apparent plasma clearance following oral administration; eGFR, estimated glomerular filtration rate; F1, nonlinear relative bioavailability; FABRY, Fabry disease status (0, no Fabry disease; 1, Fabry disease); FD, Fabry disease; FIX, parameter was fixed and not estimated; IIV, interindividual variability; Ka, absorption rate constant; popPK, population pharmacokinetics; Q/F, intercompartmental clearance; TADCO, time after dose capped at 24 hours; V_2_/F, volume of the central compartment after oral dosing; V_3_/F, volume of the peripheral compartment after oral dosing; WTCO, weight capped at and normalized to 70 kg.
(PDF)

S2 Table Parameters for the migalastat PBPK models in humans. CLspec, specific clearance; K_d_, equilibrium dissociation constant; K_off_, off rate constant; PBPK, physiologically based pharmacokinetic analysis; pKa, negative base-10 logarithm of the acid dissociation constant; UDPGT, uridine diphosphate glucuronosyltransferase.
(PDF)

S3 Table Summary of PK parameters of migalastat in urine. A_e_, amount of drug recovered in urine; CL_R_, renal clearance; ESRD, end-stage renal disease; F_e_, fraction of the dose recovered in urine; HD, hemodialysis; HDF, hemodiafiltration; PK, pharmacokinetic; NRF, normal renal function.
(PDF)

S4 Table Summary of adverse events. ESRD, end-stage renal disease; NRF, normal renal function; TEAE, treatment-emergent adverse event. ^a^Arteriovenous fistula site complication. ^b^One event each of headache and panic attack. ^c^Two events of dizziness and three events of headache.
(PDF)

S5 Table Results across all simulation scenarios. BLQ, below the limit of quantification; C_avg_, average concentration; C_max_, maximum concentration; C_trough_, concentration at the end of a dosing interval at steady state; MWF, Monday, Wednesday, Friday; MTh, Monday, Thursday; Q3D, every 3 days; Q4D, every 4 days; Q6D, every 6 days; Q12D, every 12 days; QOW, every other week; QOD, every other day; QW, every week; SuTu, Sunday, Tuesday; SuW, Sunday, Wednesday. ^a^These simulations were added after the twice QOW dosing simulations were conducted.
(PDF)

S1 Dataset (PDF)

S2 Dataset (PDF)

S1 Protocol (PDF)
