# Correction: Pharmacokinetic evaluation of single-dose migalastat in non-Fabry disease subjects with ESRD receiving dialysis treatment, and use of modeling to select dose regimens in Fabry disease subjects with ESRD receiving dialysis treatment

**DOI:** 10.1371/journal.pone.0322315

**Published:** 2025-04-08

**Authors:** Franklin K. Johnson, Shirley Wu, Ginny Schmith, Hadis Williams, Jasmine Rutecki, Atef Halabi, Thorsten Feldkamp, Anthony Sileno

In the Materials and methods section, the headings Pharmacokinetic sample collection, Analytical method, Noncompartmental analysis and Population pharmacokinetics analysis sections should have been the subheadings of the Study Treatment.

Tables 2 and 3 are formatted incorrectly. Please see the correct Tables 2 and 3 here.

**Table 2:** Summary of PK parameters of migalastat in plasma.

PK parameter	Subjects with ESRD (n = 6)				Subjects with NRF (n = 6)
	Period 1 (“off dialysis”)^a^		Period 2 (“on dialysis”)		
	ESRD-HD	ESRD-HDF	ESRD-HD	ESRD-HDF	
C_max,24h_ (ng/mL)					
**Median** **Range** **N**	3650 1610‒4770 3	2600 1870‒3320 3	1720 816‒2500 3	1770 816‒2620 3	1455 1260‒2070 6
**t**_max_ **(h)**					
**Median** **Range** **N**	4.0 3.0‒6.0 3	6.0 4.0‒12.0 3	3.0 2.0‒6.1 3	8.0 6.0‒10.0 3	3.0 3.0‒4.0 6
**AUC**_0-24h_ **(ng·h/mL)**					
**Median** **Range** **N**	61349 26959‒85764 3	46160 37909‒55216 3	31105 14602‒36337 3	32515 16594‒46401 3	9168 6915‒15926 6
**AUC**_0-t_ **(ng·h/mL)**					
**Median** **Range** **N**	74169 35210‒121553 3	59072 52914‒76048 3	45147 26672‒67965 3	68668 33308‒79321 3	9168 6698‒16568 6
**AUC**_0-**∞**_ **(ng·h/mL)**					
**Median** **Range** **N**	73068 48688‒97448 2	115051 – 1	47544 29118‒84727 3	74400 40661‒87626 3	9237 6919‒16628 6
**t**_1/2_ **(h)**					
**Median** **Range** **N**	17.3 15.4‒19.2 2	31.1 – 1	20.9 14.7‒29.2 3	19.6 18.7‒25.1 3	3.5 2.0‒5.9 6
**CL/F (L/h)**					
**Median** **Range** **N**	2.31 1.54‒3.08 2	1.30 – 1	3.15 1.77‒5.15 3	2.02 1.71‒3.69 3	16.3 9.02‒21.7 6
**V**_z_ **/F (L)**					
**Median** **Range** **n**	59.7 34.1‒85.3 2	58.4 – 1	74.6 66.9‒155.4 3	56.9 46.2‒133.6 3	73.5 54.9‒97.1 6

**Table 3:** Summary of PK parameters of migalastat in dialysate and extraction from plasma by dialysis.

PK parameter	Subjects with ESRD (n = 6)			
	Period 1 (“off dialysis”)		Period 2 (“on dialysis”)	
	ESRD-HD	ESRD-HDF	ESRD-HD	ESRD-HDF
**F**_e_ **D (%)**				
**Median** **Range** **N**	21.8 6.37–25.3 3	22.6 12.5–27.9 3	32.8 17.9‒45.4 3	17.1 15.7‒26.3 3
**CL**_**D**_ **(L/h)**				
**Median** **Range** **N**	6.13 6.01‒10.8 3	10.2 7.39‒12.2 3	10.6 9.44‒11.5 3	11.9 6.60‒12.3 3
**AUC**_inlet_ **(ng·h/mL)**				
**Median** **Range** **N**	2859 1342‒5255 3	2465 2151‒3422 3	4756 2453‒7354 3	3456 2366‒4062 3
**AUC**_outlet_ **(ng·h/mL)**				
**Median** **Range** **N**	784 251‒1363 3	732 607‒890 3	924 376‒1694 3	993 576‒1005 3
**ED (%)**				
**Median** **Range** **N**	74.1 72.6‒81.3 3	71.8 70.3‒74.0 3	80.6 77.0‒84.7 3	75.6 70.9‒75.6 3

S1 Checklist is uploaded incorrectly. Please view the correct S1 Checklist below.

## Supporting information

S1 Checklist TREND statement checklist. (PDF)
