# Supplementary material for: Pharmacokinetic evaluation of single-dose migalastat in non-Fabry disease subjects with ESRD receiving dialysis treatment, and use of modeling to select dose regimens in Fabry disease subjects with ESRD receiving dialysis treatment
Source: PLoS One. 2024 Dec 5;19(12):e0314030. doi: 10.1371/journal.pone.0314030 (PMC11620666; doi:10.1371/journal.pone.0314030)
Supplement: S1 Table — ALAG, absorption lag time; CL/F, apparent plasma clearance following oral administration; eGFR, estimated glomerular filtration rate; F1, nonlinear relative bioavailability; FABRY, Fabry disease status (0, no Fabry disease; 1, Fabry disease); FD, Fabry disease; FIX, parameter was fixed and not estimated; IIV, interindividual variability; Ka, absorption rate constant; popPK, population pharmacokinetics; Q/F, intercompartmental clearance; TADCO, time after dose capped at 24 hours; V2/F, volume of the central compartment after oral dosing; V3/F, volume of the peripheral compartment after oral dosing; WTCO, weight capped at and normalized to 70 kg. (PDF) [file pone.0314030.s002.pdf]

S1 Table. PopPK model structure and parameter estimates.

Parameter	Equation	Label	Estimate (relative standard error) [shrinkage]	
			Fixed effect	IIV or residual
**Ka (h^{-1})**	$\text{Ka} = max\begin{cases} 0.00001 \\ \theta_1 \times \exp(\eta_1) + (\theta_2 \times \exp\,(\eta_2) \times TADCO \end{cases}$	1. Intercept on Ka	0.256 (9%)	60.4% (11%) [30%]
		2. Slope for time-dependent effect on Ka (with a maximum time of 24 h)	0.284 (9%)	60.7% (9%) [31%]
**ALAG1 (h)**	$ALAG1 = \theta_3$	3. Lag time	0.175 (5%)	–
**F1**	$F1 = \theta_4$	4. Bioavailability	1 FIX	–
**CL/F (L/h)**	$\frac{CL}{F} = if(eGFR\ > 120, \theta_5, \theta_6 \times \frac{eGFR}{90})^{\theta}{}_7 \times (1 +\ \theta_8\ \times (1 - FABRY)) \times WTCO^{\theta_9} \times \exp(\eta_5)$	5. Coefficient for eGFR effect on CL/F for FD subjects with eGFR > 120 mL/min/1.73 m^2 and weight ≥ 70 kg	20.9 (17%)	28.8% (7%) [4%]
		6. Coefficient for eGFR effect on CL/F for FD subjects with	18.6 (16%)	–

		eGFR = 90 mL/min/1.73 m^2 and weight ≥ 70 kg		
		7. Exponent for eGFR effect on CL/F	0.922 (6%)	–
		8. Fractional change in CL/F in subjects without FD	−0.15 (25%)	–
		9. Exponent for weight effect on CL/F and Q/F	0.75 FIX	–
**Q/F (L/h)**	$\frac{Q}{F} = \theta_{10} \times WTCO^{\theta_9}$	10. Q/F for subjects with weight ≥ 70 kg	1 (5%)	-
**V_2/F (L)**	$\frac{V_2}{F} = \theta_{11} \times \left(1 + \theta_{12} \times (1 - FABRY)\right) \times WTCO^{\theta_{13}} \times \exp(\eta_{11})$	11. Typical value for V_2/F for FD subjects with weight ≥ 70 kg	70.1 (5%)	34.5% (6%) [7%]
		12. Fractional change in V_2/F in subjects without FD	−0.306 (13%)	–
		13. Exponent for weight effect on V_2/F and V_3/F	1 FIX	–
**V_3/F (L)**				

	$\frac{V_3}{F} = \theta_{14} \times WTCO^{\theta_{13}}$	14. V_3/F for FD subjects with weight ≥ 70 kg	27.5 (12%)	–
**Residual error**	Combined proportional and additive residual error	Proportional (%)	–	26.2% (5.5%) [7%]
		Additive (ng/mL)	–	2.55 (30%) [7%]

ALAG, absorption lag time; CL/F, apparent plasma clearance following oral administration; eGFR, estimated glomerular filtration rate; F1, nonlinear relative bioavailability; FABRY, Fabry disease status (0, no Fabry disease; 1, Fabry disease); FD, Fabry disease; FIX, parameter was fixed and not estimated; IIV, interindividual variability; Ka, absorption rate constant; popPK, population pharmacokinetics; Q/F, intercompartmental clearance; TADCO, time after dose capped at 24 hours; V_2/F, volume of the central compartment after oral dosing; V_3/F, volume of the peripheral compartment after oral dosing; WTCO, weight capped at and normalized to 70 kg.
