# Supplementary material for: Pharmacokinetic evaluation of single-dose migalastat in non-Fabry disease subjects with ESRD receiving dialysis treatment, and use of modeling to select dose regimens in Fabry disease subjects with ESRD receiving dialysis treatment
Source: PLoS One. 2024 Dec 5;19(12):e0314030. doi: 10.1371/journal.pone.0314030 (PMC11620666; doi:10.1371/journal.pone.0314030)
Supplement: S2 Table — CLspec, specific clearance; Kd, equilibrium dissociation constant; Koff, off rate constant; PBPK, physiologically based pharmacokinetic analysis; pKa, negative base-10 logarithm of the acid dissociation constant; UDPGT, uridine diphosphate glucuronosyltransferase. (PDF) [file pone.0314030.s003.pdf]

S2 Table. Parameters for the migalastat PBPK models in humans.

Parameter	Full dataset model	Steady-state dataset model
Basic physiochemical properties		
Is it a small molecule?	Yes	
Lipophilicity	−1.70	−3.03
Fraction unbound (%)	100	
Molecular weight (g/mol)	163.17	
Has halogens?	No	
Compound type	Monoprotic base	
pKa (basic)	7.47	
Solubility	500 g/L between pH 1.2 and 7.5	
Partition coefficient calculation method	Schmitt	
Cellular permeabilities calculation method	PK-Sim® standard	
Biological properties		
Specific intestinal permeability (cm/min)	1×10^{-4}	2×10^{-5}
Specific binding – lysosome: K_d (M)	0.01	0.01
Specific binding – lysosome: K_{off} (min^{-1})	1.6×10^{-3}	2×10^{-3}
Hepatic clearance – specific clearance (min^{-1})	0.05	0.04
Extrahepatic clearance – UDPGT in brain CLspec/[enzyme] (L/µmol/min)	2.10×10^{-3}	2.10×10^{-3}
Tissue partition coefficient (intracellular: plasma)		
Heart	0.01	5×10^{-3}
Kidney	4.00	0.76
Liver	0.10	5×10^{-3}

Muscle	0.50	0.79
Skin	0.54	0.54
Brain	0.81	0.80

CLspec, specific clearance; K_d, equilibrium dissociation constant; K_{off}, off rate constant; PBPK, physiologically based pharmacokinetic analysis; pKa, negative base-10 logarithm of the acid dissociation constant; UDPGT, uridine diphosphate glucuronosyltransferase.
