# Supplementary material for: Pharmacokinetic evaluation of single-dose migalastat in non-Fabry disease subjects with ESRD receiving dialysis treatment, and use of modeling to select dose regimens in Fabry disease subjects with ESRD receiving dialysis treatment
Source: PLoS One. 2024 Dec 5;19(12):e0314030. doi: 10.1371/journal.pone.0314030 (PMC11620666; doi:10.1371/journal.pone.0314030)
Supplement: S3 Table — Ae, amount of drug recovered in urine; CLR, renal clearance; ESRD, end-stage renal disease; Fe, fraction of the dose recovered in urine; HD, hemodialysis; HDF, hemodiafiltration; PK, pharmacokinetic; NRF, normal renal function. (PDF) [file pone.0314030.s004.pdf]

S3 Table. Summary of PK parameters of migalastat in urine.

PK parameter	Subjects with ESRD (n = 6)				Subjects with NRF (n = 6)
	Period 1 (“off dialysis”)		Period 2 (“on dialysis”)		
	ESRD-HD	ESRD-HDF	ESRD-HD	ESRD-HDF	
**A_e (mg)**					
Median	9.4	3.8	6.0	0.8	59.4
Range	2.1–20.3	1.3–5.4	0.1–8.9	0.7–2.9	35.8–83.0
n	3	3	3	3	6
**F_e (%)**					
Median	6.3	2.6	4.0	0.6	39.6
Range	1.4–13.5	0.9–3.6	0.1–5.9	0.4–1.9	23.9–55.3
n	3	3	3	3	6
**CL_R (L/h)**					
Median	0.266	0.065	0.198	0.025	5.492
Range	0.017–0.273	0.025–0.070	0.002–0.226	0.008–0.042	4.928–7.022
n	3	3	3	3	6

A_e, amount of drug recovered in urine; CL_R, renal clearance; ESRD, end-stage renal disease; F_e, fraction of the dose recovered in urine; HD, hemodialysis; HDF, hemodiafiltration; PK, pharmacokinetic; NRF, normal renal function.
