# Supplementary material for: Pharmacokinetic evaluation of single-dose migalastat in non-Fabry disease subjects with ESRD receiving dialysis treatment, and use of modeling to select dose regimens in Fabry disease subjects with ESRD receiving dialysis treatment
Source: PLoS One. 2024 Dec 5;19(12):e0314030. doi: 10.1371/journal.pone.0314030 (PMC11620666; doi:10.1371/journal.pone.0314030)
Supplement: S4 Table — ESRD, end-stage renal disease; NRF, normal renal function; TEAE, treatment-emergent adverse event. aArteriovenous fistula site complication. bOne event each of headache and panic attack. cTwo events of dizziness and three events of headache. (PDF) [file pone.0314030.s005.pdf]

S4 Table. Summary of adverse events.

	Subjects with ESRD (n = 6)		Subjects with NRF (n = 6)	Total (n = 12)
	Period 1 ("off dialysis")	Period 2 ("on dialysis")		
**Subjects with any TEAE, n (%)**	3 (50.0)	3 (50.0)	2 (33.3)	6 (50.0)
**Number of TEAEs, n**	3	3	2	8
**Number of mild TEAEs, n**	2	3	2	7
**Number of moderate TEAEs, n**	1	0	0	1
**Number of serious TEAEs, n**	0	0	0	0
**Number of treatment-related TEAEs, n**				
**Unrelated**	1	0	0	1[a]
**Unlikely related**	1	1	0	2[b]
**Possibly related**	1	2	2	5[c]
**TEAEs leading to study discontinuation**	0	0	0	0
**TEAEs leading to death**	0	0	0	0

ESRD, end-stage renal disease; NRF, normal renal function; TEAE, treatment-emergent adverse event. [a]Arteriovenous fistula site complication. [b]One event each of headache and panic attack. [c]Two events of dizziness and three events of headache.
