# Supplementary material for: Pharmacokinetic evaluation of single-dose migalastat in non-Fabry disease subjects with ESRD receiving dialysis treatment, and use of modeling to select dose regimens in Fabry disease subjects with ESRD receiving dialysis treatment
Source: PLoS One. 2024 Dec 5;19(12):e0314030. doi: 10.1371/journal.pone.0314030 (PMC11620666; doi:10.1371/journal.pone.0314030)
Supplement: S5 Table — BLQ, below the limit of quantification; Cavg, average concentration; Cmax, maximum concentration; Ctrough, concentration at the end of a dosing interval at steady state; MWF, Monday, Wednesday, Friday; MTh, Monday, Thursday; Q3D, every 3 days; Q4D, every 4 days; Q6D, every 6 days; Q12D, every 12 days; QOW, every other week; QOD, every other day; QW, every week; SuTu, Sunday, Tuesday; SuW, Sunday, Wednesday. aThese simulations were added after the twice QOW dosing simulations were conducted. (PDF) [file pone.0314030.s006.pdf]

**S5 Table. Results across all simulation scenarios.**

| **Scenario** | **Dialysis frequency** | **Migalastat regimen** | **Bioequivalent?** | | **Subjects (%)** | |
|---|---|---|---|---|---|---|
| | | | $C_{avg}$ | $C_{max}$ | $C_{max}$ > 10 µM | $C_{trough}$ BLQ |
| *Prespecified scenarios* | | | | | | |
| **1** | N/A (control) | 123 mg QOD | Reference | Reference | 17 | 44 |
| **2** | Q3D | 123 mg QOD | High | High | 59−77 | 0 |
| **3** | QOD | 123 mg QOD | High | High | 82 | 0 |
| **4** | QOD | 123 mg Q4D | High | High | 65 | 0 |
| **5** | Q3D | 123 mg Q3D | High | High | 71 | 0 |
| *Extended dosing interval scenarios* | | | | | | |
| **6** | QOD | 49 mg Q4D | Slightly high | Low | 1 | 0 |
| **7** | QOD | 65 mg Q6D | Yes | Slightly low | 4 | 4 |
| **8** | QOD | 123 mg Q12D | Yes | High | 56 | 79 |
| **9** | Q3D | 82 mg Q6D | High | Yes | 14 | 0 |
| **10** | Q3D | 123 mg Q12D | Yes | High | 59 | 60 |
| *Week-based dosing and dialysis schedule* | | | | | | |
| **11** | MWF QW | 82 mg QW | Yes | Yes | 15 | 8 |
| **12** | MWF QW | 123 mg QOW | Yes | High | 56 | 92 |
| **13** | MTh QW | 82 mg QW | Slightly high | Yes | 12 | 7 |
| **14** | MTh QW | 82 mg QOW | Low | Yes | 8 | 90 |

| | | | | | | |
|---|---|---|---|---|---|---|
| **15** | MTh QW | 123 mg QOW | Yes | High | 49 | 78 |
| **20**[a] | MWF QW | 123 mg QW | High | High | 53 | 2 |
| **21**[a] | MTh QW | 123 mg QW | High | High | 58 | 1 |
| *Twice QOW dosing and week-based dialysis schedule* | | | | | | |
| **16** | MTh QW | 82 mg SuW QOW | Yes | Yes | 12 | 56 |
| **17** | MWF QW | 82 mg SuW QOW | Yes | Yes | 15 | 62 |
| **18** | MTh QW | 82 mg SuTu QOW | Slightly high | Yes | 17 | 54 |
| **19** | MWF QW | 82 mg SuTu QOW | Yes | Yes | 11 | 75 |

BLQ, below the limit of quantification; $C_{avg}$, average concentration; $C_{max}$, maximum concentration; $C_{trough}$, concentration at the end of a dosing interval at steady state; MWF, Monday, Wednesday, Friday; MTh, Monday, Thursday; Q3D, every 3 days; Q4D, every 4 days; Q6D, every 6 days; Q12D, every 12 days; QOW, every other week; QOD, every other day; QW, every week; SuTu, Sunday, Tuesday; SuW, Sunday, Wednesday.

[a]These simulations were added after the twice QOW dosing simulations were conducted.
