# Supplementary material for: Pharmacokinetic evaluation of single-dose migalastat in non-Fabry disease subjects with ESRD receiving dialysis treatment, and use of modeling to select dose regimens in Fabry disease subjects with ESRD receiving dialysis treatment
Source: PLoS One. 2024 Dec 5;19(12):e0314030. doi: 10.1371/journal.pone.0314030 (PMC11620666; doi:10.1371/journal.pone.0314030)
Supplement: S1 Dataset — (PDF) [file pone.0314030.s007.pdf]

CLINICAL STUDY REPORT

Study Title:	An Open-label Study to Evaluate the Pharmacokinetics and Safety of Migalastat in Non-Fabry End-Stage Renal Disease Subjects Receiving Dialysis and Matched Healthy Subjects
Investigational Product:	Migalastat hydrochloride
Indication Studied	Not applicable
Description of Study:	A Phase 1, open-label, non-randomized study in non-Fabry subjects with end-stage renal disease on hemodialysis (standard hemodialysis or hemodiafiltration) and matched control subjects with normal renal function
Name of Sponsor:	Amicus Therapeutics
Protocol Number:	AT1001-035
Development Phase:	1
First Subject Enrolled:	05 June 2019
Last Subject Completed:	20 December 2019

[redacted]

GCP Compliance:	This study was performed in compliance with Good Clinical Practice, including archiving of essential documents
Date of Report:	03 September 2020

Table 14.2.1.1: Individual migalastat plasma concentrations [ng/mL] by time point

Pharmacokinetic Analysis Set

Group ESRD-STA, Period OFF (dialysis 24:05h to 28:05h)

Subject No.	Predose	1h	2h	3h	4h	6h	8h	10h	12h
5	BLLQ	2200	4330	4770	4760	4170	4340	3670	3660
7	BLLQ	703	1030	1420	1610	1540	1410	1300	1170
10	BLLQ	1030	2140	3130	3610	3650	3290	3040	2840
n	0	3	3	3	3	3	3	3	3
Mean	n.c.	1311.0	2500.0	3106.7	3326.7	3120.0	3013.3	2670.0	2556.7
SD	n.c.	787.1	1679.2	1675.1	1594.0	1392.8	1484.5	1227.6	1268.9
Geo. Mean	n.c.	1167.9	2121.2	2767.7	3024.5	2861.9	2720.4	2438.7	2299.7

ESRD: End stage renal disease, ESRD-STA: standard hemodialysis, ESRD-HDF: hemodiafiltration,
NRF: Normal renal function, NRF-mSTA = subjects with NRF matched to ESRD-STA, NRF-mHDF = subjects with NRF matched to ESRD-HDF, OFF = off-dialysis (period 1), ON = on-dialysis (period 2),
n.c. = not computed, BLLQ = below lower limit of quantification, Geo. = Geometric,
n = number of values >= lower limit of quantification.

Program: T14_2_1_1_plasmaC.sas (Page 1 of 20)

Table 14.2.1.1: Individual migalastat plasma concentrations [ng/mL] by time point

Pharmacokinetic Analysis Set

Group ESRD-STA, Period OFF (dialysis 24:05h to 28:05h)

Subject No.	Predose	1h	2h	3h	4h	6h	8h	10h	12h
Geo. SD	n.c.	1.8	2.1	1.8	1.8	1.7	1.8	1.7	1.8

ESRD: End stage renal disease, ESRD-STA: standard hemodialysis, ESRD-HDF: hemodiafiltration,
NRF: Normal renal function, NRF-mSTA = subjects with NRF matched to ESRD-STA, NRF-mHDF = subjects with NRF matched to ESRD-HDF, OFF = off-dialysis (period 1), ON = on-dialysis (period 2),
n.c. = not computed, BLLQ = below lower limit of quantification, Geo. = Geometric,
n = number of values >= lower limit of quantification.

Program: T14_2_1_1_plasmaC.sas (Page 2 of 20)

Table 14.2.1.1: Individual migalastat plasma concentrations [ng/mL] by time point

Pharmacokinetic Analysis Set

Group ESRD-STA, Period OFF (dialysis 24:05h to 28:05h)

Subject No.	24h	24:05h	25h	26h	27h	28h	28:05h	48h	72h
5	2890	2820	2130	1480	1260	881	806	811	287
7	788	754	526	340	230	170	165	187	72.9
10	1630	1540	1040	756	497	339	276	285	46.1
n	3	3	3	3	3	3	3	3	3
Mean	1769.3	1704.7	1232.0	858.7	662.3	463.3	415.7	427.7	135.3
SD	1057.9	1042.8	819.1	576.9	534.5	371.4	342.6	335.6	132.0
Geo. Mean	1548.4	1485.0	1052.3	724.6	524.2	370.3	332.3	350.9	98.8

ESRD: End stage renal disease, ESRD-STA: standard hemodialysis, ESRD-HDF: hemodiafiltration,
NRF: Normal renal function, NRF-mSTA = subjects with NRF matched to ESRD-STA, NRF-mHDF = subjects with NRF matched to ESRD-HDF, OFF = off-dialysis (period 1), ON = on-dialysis (period 2),
n.c. = not computed, BLLQ = below lower limit of quantification, Geo. = Geometric,
n = number of values >= lower limit of quantification.

Program: T14_2_1_1_plasmaC.sas (Page 2 of 20)

Table 14.2.1.1: Individual migalastat plasma concentrations [ng/mL] by time point

Pharmacokinetic Analysis Set

Group ESRD-STA, Period OFF (dialysis 24:05h to 28:05h)

Subject No.	24h	24:05h	25h	26h	27h	28h	28:05h	48h	72h
Geo. SD	1.9	1.9	2.0	2.1	2.3	2.3	2.2	2.1	2.6

ESRD: End stage renal disease, ESRD-STA: standard hemodialysis, ESRD-HDF: hemodiafiltration,
NRF: Normal renal function, NRF-mSTA = subjects with NRF matched to ESRD-STA, NRF-mHDF = subjects with NRF matched to ESRD-HDF, OFF = off-dialysis (period 1), ON = on-dialysis (period 2),
n.c. = not computed, BLLQ = below lower limit of quantification, Geo. = Geometric,
n = number of values >= lower limit of quantification.

Program: T14_2_1_1_plasmaC.sas (Page 3 of 20)

Table 14.2.1.1: Individual migalastat plasma concentrations [ng/mL] by time point

Pharmacokinetic Analysis Set

Group ESRD-HDF, Period OFF (dialysis 24:05h to 28:05h)

Subject No.	Predose	1h	2h	3h	4h	6h	8h	10h	12h
1	BLLQ	830	1380	2290	2600	2500	2460	2190	2020
2	BLLQ	883	1080	1330	1590	1750	1810	1770	1870
8	BLLQ	134	598	1430	2520	3320	2860	2710	2650
n	0	3	3	3	3	3	3	3	3
Mean	n.c.	615.7	1019.3	1683.3	2236.7	2523.3	2376.7	2223.3	2180.0
SD	n.c.	418.0	394.5	527.8	561.5	785.3	529.9	470.9	413.9
Geo. Mean	n.c.	461.4	962.4	1633.1	2184.0	2439.9	2335.2	2190.1	2155.2

ESRD: End stage renal disease, ESRD-STA: standard hemodialysis, ESRD-HDF: hemodiafiltration,
NRF: Normal renal function, NRF-mSTA = subjects with NRF matched to ESRD-STA, NRF-mHDF = subjects with NRF matched to ESRD-HDF, OFF = off-dialysis (period 1), ON = on-dialysis (period 2),
n.c. = not computed, BLLQ = below lower limit of quantification, Geo. = Geometric,
n = number of values >= lower limit of quantification.

Program: T14_2_1_1_plasmaC.sas (Page 3 of 20)

Table 14.2.1.1: Individual migalastat plasma concentrations [ng/mL] by time point

Pharmacokinetic Analysis Set

Group ESRD-HDF, Period OFF (dialysis 24:05h to 28:05h)

Subject No.	Predose	1h	2h	3h	4h	6h	8h	10h	12h
Geo. SD	n.c.	2.9	1.5	1.3	1.3	1.4	1.3	1.2	1.2

ESRD: End stage renal disease, ESRD-STA: standard hemodialysis, ESRD-HDF: hemodiafiltration,
NRF: Normal renal function, NRF-mSTA = subjects with NRF matched to ESRD-STA, NRF-mHDF = subjects with NRF matched to ESRD-HDF, OFF = off-dialysis (period 1), ON = on-dialysis (period 2),
n.c. = not computed, BLLQ = below lower limit of quantification, Geo. = Geometric,
n = number of values >= lower limit of quantification.

Program: T14_2_1_1_plasmaC.sas (Page 4 of 20)

Table 14.2.1.1: Individual migalastat plasma concentrations [ng/mL] by time point

Pharmacokinetic Analysis Set

Group ESRD-HDF, Period OFF (dialysis 24:05h to 28:05h)

Subject No.	24h	24:05h	25h	26h	27h	28h	28:05h	48h	72h
1	1560	1530	943	575	443	273	196	308	99.3
2	1420	1410	938	611	398	282	266	362	129
8	2160	2200	1400	829	567	363	360	415	357
n	3	3	3	3	3	3	3	3	3
Mean	1713.3	1713.3	1093.7	671.7	469.3	306.0	274.0	361.7	195.1
SD	393.1	425.7	265.3	137.4	87.5	49.6	82.3	53.5	141.0
Geo. Mean	1685.1	1680.5	1073.9	662.9	464.1	303.5	265.8	359.0	166.0

ESRD: End stage renal disease, ESRD-STA: standard hemodialysis, ESRD-HDF: hemodiafiltration,
NRF: Normal renal function, NRF-mSTA = subjects with NRF matched to ESRD-STA, NRF-mHDF = subjects with NRF matched to ESRD-HDF, OFF = off-dialysis (period 1), ON = on-dialysis (period 2),
n.c. = not computed, BLLQ = below lower limit of quantification, Geo. = Geometric,
n = number of values >= lower limit of quantification.

Program: T14_2_1_1_plasmaC.sas (Page 4 of 20)

Table 14.2.1.1: Individual migalastat plasma concentrations [ng/mL] by time point

Pharmacokinetic Analysis Set

Group ESRD-HDF, Period OFF (dialysis 24:05h to 28:05h)

Subject No.	24h	24:05h	25h	26h	27h	28h	28:05h	48h	72h
Geo. SD	1.2	1.3	1.3	1.2	1.2	1.2	1.4	1.2	2.0

ESRD: End stage renal disease, ESRD-STA: standard hemodialysis, ESRD-HDF: hemodiafiltration,
NRF: Normal renal function, NRF-mSTA = subjects with NRF matched to ESRD-STA, NRF-mHDF = subjects with NRF matched to ESRD-HDF, OFF = off-dialysis (period 1), ON = on-dialysis (period 2),
n.c. = not computed, BLLQ = below lower limit of quantification, Geo. = Geometric,
n = number of values >= lower limit of quantification.

Program: T14_2_1_1_plasmaC.sas (Page 5 of 20)

Table 14.2.1.1: Individual migalastat plasma concentrations [ng/mL] by time point

Pharmacokinetic Analysis Set

Group ESRD-pooled, Period OFF (dialysis 24:05h to 28:05h)

Subject No.	Predose	1h	2h	3h	4h	6h	8h	10h	12h
1	BLLQ	830	1380	2290	2600	2500	2460	2190	2020
2	BLLQ	883	1080	1330	1590	1750	1810	1770	1870
5	BLLQ	2200	4330	4770	4760	4170	4340	3670	3660
7	BLLQ	703	1030	1420	1610	1540	1410	1300	1170
8	BLLQ	134	598	1430	2520	3320	2860	2710	2650
10	BLLQ	1030	2140	3130	3610	3650	3290	3040	2840
n	0	6	6	6	6	6	6	6	6

ESRD: End stage renal disease, ESRD-STA: standard hemodialysis, ESRD-HDF: hemodiafiltration, NRF: Normal renal function, NRF-mSTA = subjects with NRF matched to ESRD-STA, NRF-mHDF = subjects with NRF matched to ESRD-HDF, OFF = off-dialysis (period 1), ON = on-dialysis (period 2), n.c. = not computed, BLLQ = below lower limit of quantification, Geo. = Geometric, n = number of values >= lower limit of quantification.

Program: T14_2_1_1_plasmaC.sas (Page 5 of 20)

Table 14.2.1.1: Individual migalastat plasma concentrations [ng/mL] by time point

Pharmacokinetic Analysis Set

Group ESRD-pooled, Period OFF (dialysis 24:05h to 28:05h)

Subject No.	Predose	1h	2h	3h	4h	6h	8h	10h	12h
Mean	n.c.	963.3	1759.7	2395.0	2781.7	2821.7	2695.0	2446.7	2368.3
SD	n.c.	680.2	1359.4	1357.1	1224.3	1062.7	1056.1	866.8	869.0
Geo. Mean	n.c.	734.1	1428.8	2126.0	2570.1	2642.5	2520.5	2311.1	2226.2
Geo. SD	n.c.	2.5	2.0	1.7	1.5	1.5	1.5	1.5	1.5

ESRD: End stage renal disease, ESRD-STA: standard hemodialysis, ESRD-HDF: hemodiafiltration,
NRF: Normal renal function, NRF-mSTA = subjects with NRF matched to ESRD-STA, NRF-mHDF = subjects with NRF matched to ESRD-HDF, OFF = off-dialysis (period 1), ON = on-dialysis (period 2),
n.c. = not computed, BLLQ = below lower limit of quantification, Geo. = Geometric,
n = number of values >= lower limit of quantification.

Program: T14_2_1_1_plasmaC.sas (Page 6 of 20)

Table 14.2.1.1: Individual migalastat plasma concentrations [ng/mL] by time point

Pharmacokinetic Analysis Set

Group ESRD-pooled, Period OFF (dialysis 24:05h to 28:05h)

Subject No.	24h	24:05h	25h	26h	27h	28h	28:05h	48h	72h
1	1560	1530	943	575	443	273	196	308	99.3
2	1420	1410	938	611	398	282	266	362	129
5	2890	2820	2130	1480	1260	881	806	811	287
7	788	754	526	340	230	170	165	187	72.9
8	2160	2200	1400	829	567	363	360	415	357
10	1630	1540	1040	756	497	339	276	285	46.1
n	6	6	6	6	6	6	6	6	6

ESRD: End stage renal disease, ESRD-STA: standard hemodialysis, ESRD-HDF: hemodiafiltration, NRF: Normal renal function, NRF-mSTA = subjects with NRF matched to ESRD-STA, NRF-mHDF = subjects with NRF matched to ESRD-HDF, OFF = off-dialysis (period 1), ON = on-dialysis (period 2), n.c. = not computed, BLLQ = below lower limit of quantification, Geo. = Geometric, n = number of values >= lower limit of quantification.

Program: T14_2_1_1_plasmaC.sas (Page 7 of 20)

Table 14.2.1.1: Individual migalastat plasma concentrations [ng/mL] by time point

Pharmacokinetic Analysis Set

Group ESRD-pooled, Period OFF (dialysis 24:05h to 28:05h)

Subject No.	24h	24:05h	25h	26h	27h	28h	28:05h	48h	72h
Mean	1741.3	1709.0	1162.8	765.2	565.8	384.7	344.8	394.7	165.2
SD	714.4	712.4	549.8	388.8	358.5	252.2	235.9	217.9	126.5
Geo. Mean	1615.3	1579.7	1063.0	693.0	493.2	335.2	297.2	355.0	128.1
Geo. SD	1.5	1.6	1.6	1.6	1.7	1.7	1.8	1.6	2.2

ESRD: End stage renal disease, ESRD-STA: standard hemodialysis, ESRD-HDF: hemodiafiltration, NRF: Normal renal function, NRF-mSTA = subjects with NRF matched to ESRD-STA, NRF-mHDF = subjects with NRF matched to ESRD-HDF, OFF = off-dialysis (period 1), ON = on-dialysis (period 2), n.c. = not computed, BLLQ = below lower limit of quantification, Geo. = Geometric, n = number of values >= lower limit of quantification.

Program: T14_2_1_1_plasmaC.sas (Page 8 of 20)

Table 14.2.1.1: Individual migalastat plasma concentrations [ng/mL] by time point

Pharmacokinetic Analysis Set

Group ESRD-STA, Period ON (dialysis 0:05h to 4:05h)

Subject No.	Predose	0:05h	1h	2h	3h	4h	4:05h	6h	8h
5	66.2	66.2	1480	2500	2370	1690	1600	1630	1560
7	7.90	6.84	469	733	782	681	717	816	784
10	39.1	36.0	937	1170	1720	1520	1510	1720	1650
n	3	3	3	3	3	3	3	3	3
Mean	37.7	36.3	962.0	1467.7	1624.0	1297.0	1275.7	1388.7	1331.3
SD	29.2	29.7	506.0	920.3	798.3	540.2	485.9	498.0	476.1
Geo. Mean	27.3	25.4	866.4	1289.5	1471.7	1204.9	1201.0	1317.7	1263.7

ESRD: End stage renal disease, ESRD-STA: standard hemodialysis, ESRD-HDF: hemodiafiltration, NRF: Normal renal function, NRF-mSTA = subjects with NRF matched to ESRD-STA, NRF-mHDF = subjects with NRF matched to ESRD-HDF, OFF = off-dialysis (period 1), ON = on-dialysis (period 2), n.c. = not computed, BLLQ = below lower limit of quantification, Geo. = Geometric, n = number of values >= lower limit of quantification.

Program: T14_2_1_1_plasmaC.sas (Page 9 of 20)

Table 14.2.1.1: Individual migalastat plasma concentrations [ng/mL] by time point

Pharmacokinetic Analysis Set

Group ESRD-STA, Period ON (dialysis 0:05h to 4:05h)

Subject No.	Predose	0:05h	1h	2h	3h	4h	4:05h	6h	8h
Geo. SD	3.0	3.2	1.8	1.9	1.8	1.6	1.6	1.5	1.5

ESRD: End stage renal disease, ESRD-STA: standard hemodialysis, ESRD-HDF: hemodiafiltration,
NRF: Normal renal function, NRF-mSTA = subjects with NRF matched to ESRD-STA, NRF-mHDF = subjects with NRF matched to ESRD-HDF, OFF = off-dialysis (period 1), ON = on-dialysis (period 2),
n.c. = not computed, BLLQ = below lower limit of quantification, Geo. = Geometric,
n = number of values >= lower limit of quantification.

Program: T14_2_1_1_plasmaC.sas (Page 10 of 20)

Table 14.2.1.1: Individual migalastat plasma concentrations [ng/mL] by time point

Pharmacokinetic Analysis Set

Group ESRD-STA, Period ON (dialysis 0:05h to 4:05h)

Subject No.	10h	12h	24h	25h	26h	27h	28h	48h	72h
5	1510	1500	1270	1260	1310	1290	1210	442	398
7	676	613	453	424	425	437	398	255	81.1
10	1530	1370	940	854	847	787	784	79.7	113
n	3	3	3	3	3	3	3	3	3
Mean	1238.7	1161.0	887.7	846.0	860.7	838.0	797.3	258.9	197.4
SD	487.4	479.0	411.0	418.1	442.7	428.8	406.2	181.2	174.5
Geo. Mean	1160.2	1080.0	814.7	769.8	778.4	762.7	722.8	207.9	153.9

ESRD: End stage renal disease, ESRD-STA: standard hemodialysis, ESRD-HDF: hemodiafiltration,
NRF: Normal renal function, NRF-mSTA = subjects with NRF matched to ESRD-STA, NRF-mHDF = subjects
with NRF matched to ESRD-HDF, OFF = off-dialysis (period 1), ON = on-dialysis (period 2),
n.c. = not computed, BLLQ = below lower limit of quantification, Geo. = Geometric,
n = number of values >= lower limit of quantification.

Program: T14_2_1_1_plasmaC.sas (Page 10 of 20)

Table 14.2.1.1: Individual migalastat plasma concentrations [ng/mL] by time point

Pharmacokinetic Analysis Set

Group ESRD-STA, Period ON (dialysis 0:05h to 4:05h)

Subject No.	10h	12h	24h	25h	26h	27h	28h	48h	72h
Geo. SD	1.6	1.6	1.7	1.7	1.8	1.7	1.8	2.4	2.3

ESRD: End stage renal disease, ESRD-STA: standard hemodialysis, ESRD-HDF: hemodiafiltration,
NRF: Normal renal function, NRF-mSTA = subjects with NRF matched to ESRD-STA, NRF-mHDF = subjects with NRF matched to ESRD-HDF, OFF = off-dialysis (period 1), ON = on-dialysis (period 2),
n.c. = not computed, BLLQ = below lower limit of quantification, Geo. = Geometric,
n = number of values >= lower limit of quantification.

Program: T14_2_1_1_plasmaC.sas (Page 11 of 20)

Table 14.2.1.1: Individual migalastat plasma concentrations [ng/mL] by time point

Pharmacokinetic Analysis Set

Group ESRD-HDF, Period ON (dialysis 0:05h to 4:05h)

Subject No.	Predose	0:05h	1h	2h	3h	4h	4:05h	6h	8h
1	59.9	55.4	243	604	1820	1870	2090	2620	2560
2	71.6	65.4	540	678	640	496	456	607	731
8	43.8	45.3	249	866	1350	1450	1410	1740	1770
n	3	3	3	3	3	3	3	3	3
Mean	58.4	55.4	344.0	716.0	1270.0	1272.0	1318.7	1655.7	1687.0
SD	14.0	10.1	169.8	135.1	594.1	704.1	820.8	1009.1	917.3
Geo. Mean	57.3	54.8	319.7	707.8	1162.9	1103.8	1103.5	1403.9	1490.7

ESRD: End stage renal disease, ESRD-STA: standard hemodialysis, ESRD-HDF: hemodiafiltration,
NRF: Normal renal function, NRF-mSTA = subjects with NRF matched to ESRD-STA, NRF-mHDF = subjects with NRF matched to ESRD-HDF, OFF = off-dialysis (period 1), ON = on-dialysis (period 2),
n.c. = not computed, BLLQ = below lower limit of quantification, Geo. = Geometric,
n = number of values >= lower limit of quantification.

Program: T14_2_1_1_plasmaC.sas (Page 11 of 20)

Table 14.2.1.1: Individual migalastat plasma concentrations [ng/mL] by time point

Pharmacokinetic Analysis Set

Group ESRD-HDF, Period ON (dialysis 0:05h to 4:05h)

Subject No.	Predose	0:05h	1h	2h	3h	4h	4:05h	6h	8h
Geo. SD	1.3	1.2	1.6	1.2	1.7	2.0	2.2	2.1	1.9

ESRD: End stage renal disease, ESRD-STA: standard hemodialysis, ESRD-HDF: hemodiafiltration,
NRF: Normal renal function, NRF-mSTA = subjects with NRF matched to ESRD-STA, NRF-mHDF = subjects
with NRF matched to ESRD-HDF, OFF = off-dialysis (period 1), ON = on-dialysis (period 2),
n.c. = not computed, BLLQ = below lower limit of quantification, Geo. = Geometric,
n = number of values >= lower limit of quantification.

Program: T14_2_1_1_plasmaC.sas (Page 12 of 20)

Table 14.2.1.1: Individual migalastat plasma concentrations [ng/mL] by time point

Pharmacokinetic Analysis Set

Group ESRD-HDF, Period ON (dialysis 0:05h to 4:05h)

Subject No.	10h	12h	24h	25h	26h	27h	28h	48h	72h
1	2370	2250	1630	1560	1630	1500	1530	336	308
2	816	805	676	713	678	678	654	221	203
8	1600	1490	1210	1200	1190	1130	1130	835	203
n	3	3	3	3	3	3	3	3	3
Mean	1595.3	1515.0	1172.0	1157.7	1166.0	1102.7	1104.7	464.0	238.0
SD	777.0	722.8	478.1	425.1	476.5	411.7	438.5	326.4	60.6
Geo. Mean	1457.2	1392.3	1100.6	1101.0	1095.6	1047.4	1041.8	395.8	233.3

ESRD: End stage renal disease, ESRD-STA: standard hemodialysis, ESRD-HDF: hemodiafiltration,
NRF: Normal renal function, NRF-mSTA = subjects with NRF matched to ESRD-STA, NRF-mHDF = subjects with NRF matched to ESRD-HDF, OFF = off-dialysis (period 1), ON = on-dialysis (period 2),
n.c. = not computed, BLLQ = below lower limit of quantification, Geo. = Geometric,
n = number of values >= lower limit of quantification.

Program: T14_2_1_1_plasmaC.sas (Page 12 of 20)

Table 14.2.1.1: Individual migalastat plasma concentrations [ng/mL] by time point

Pharmacokinetic Analysis Set

Group ESRD-HDF, Period ON (dialysis 0:05h to 4:05h)

Subject No.	10h	12h	24h	25h	26h	27h	28h	48h	72h
Geo. SD	1.7	1.7	1.6	1.5	1.6	1.5	1.5	2.0	1.3

ESRD: End stage renal disease, ESRD-STA: standard hemodialysis, ESRD-HDF: hemodiafiltration,
NRF: Normal renal function, NRF-mSTA = subjects with NRF matched to ESRD-STA, NRF-mHDF = subjects with NRF matched to ESRD-HDF, OFF = off-dialysis (period 1), ON = on-dialysis (period 2),
n.c. = not computed, BLLQ = below lower limit of quantification, Geo. = Geometric,
n = number of values >= lower limit of quantification.

Program: T14_2_1_1_plasmaC.sas (Page 13 of 20)

Table 14.2.1.1: Individual migalastat plasma concentrations [ng/mL] by time point

Pharmacokinetic Analysis Set

Group ESRD-pooled, Period ON (dialysis 0:05h to 4:05h)

Subject No.	Predose	0:05h	1h	2h	3h	4h	4:05h	6h	8h
1	59.9	55.4	243	604	1820	1870	2090	2620	2560
2	71.6	65.4	540	678	640	496	456	607	731
5	66.2	66.2	1480	2500	2370	1690	1600	1630	1560
7	7.90	6.84	469	733	782	681	717	816	784
8	43.8	45.3	249	866	1350	1450	1410	1740	1770
10	39.1	36.0	937	1170	1720	1520	1510	1720	1650
n	6	6	6	6	6	6	6	6	6

ESRD: End stage renal disease, ESRD-STA: standard hemodialysis, ESRD-HDF: hemodiafiltration,
NRF: Normal renal function, NRF-mSTA = subjects with NRF matched to ESRD-STA, NRF-mHDF = subjects with NRF matched to ESRD-HDF, OFF = off-dialysis (period 1), ON = on-dialysis (period 2),
n.c. = not computed, BLLQ = below lower limit of quantification, Geo. = Geometric,
n = number of values >= lower limit of quantification.

Program: T14_2_1_1_plasmaC.sas (Page 13 of 20)

Table 14.2.1.1: Individual migalastat plasma concentrations [ng/mL] by time point

Pharmacokinetic Analysis Set

Group ESRD-pooled, Period ON (dialysis 0:05h to 4:05h)

Subject No.	Predose	0:05h	1h	2h	3h	4h	4:05h	6h	8h
Mean	48.1	45.9	653.0	1091.8	1447.0	1284.5	1297.2	1522.2	1509.2
SD	23.4	22.4	478.0	718.1	658.6	561.4	603.7	726.6	682.1
Geo. Mean	39.6	37.3	526.3	955.4	1308.2	1153.3	1151.2	1360.1	1372.5
Geo. SD	2.3	2.4	2.0	1.7	1.7	1.7	1.8	1.7	1.6

ESRD: End stage renal disease, ESRD-STA: standard hemodialysis, ESRD-HDF: hemodiafiltration, NRF: Normal renal function, NRF-mSTA = subjects with NRF matched to ESRD-STA, NRF-mHDF = subjects with NRF matched to ESRD-HDF, OFF = off-dialysis (period 1), ON = on-dialysis (period 2), n.c. = not computed, BLLQ = below lower limit of quantification, Geo. = Geometric, n = number of values >= lower limit of quantification.

Program: T14_2_1_1_plasmaC.sas (Page 14 of 20)

Table 14.2.1.1: Individual migalastat plasma concentrations [ng/mL] by time point

Pharmacokinetic Analysis Set

Group ESRD-pooled, Period ON (dialysis 0:05h to 4:05h)

Subject No.	10h	12h	24h	25h	26h	27h	28h	48h	72h
1	2370	2250	1630	1560	1630	1500	1530	336	308
2	816	805	676	713	678	678	654	221	203
5	1510	1500	1270	1260	1310	1290	1210	442	398
7	676	613	453	424	425	437	398	255	81.1
8	1600	1490	1210	1200	1190	1130	1130	835	203
10	1530	1370	940	854	847	787	784	79.7	113
n	6	6	6	6	6	6	6	6	6

ESRD: End stage renal disease, ESRD-STA: standard hemodialysis, ESRD-HDF: hemodiafiltration, NRF: Normal renal function, NRF-mSTA = subjects with NRF matched to ESRD-STA, NRF-mHDF = subjects with NRF matched to ESRD-HDF, OFF = off-dialysis (period 1), ON = on-dialysis (period 2), n.c. = not computed, BLLQ = below lower limit of quantification, Geo. = Geometric, n = number of values >= lower limit of quantification.

Program: T14_2_1_1_plasmaC.sas (Page 15 of 20)

Table 14.2.1.1: Individual migalastat plasma concentrations [ng/mL] by time point

Pharmacokinetic Analysis Set

Group ESRD-pooled, Period ON (dialysis 0:05h to 4:05h)

Subject No.	10h	12h	24h	25h	26h	27h	28h	48h	72h
Mean	1417.0	1338.0	1029.8	1001.8	1013.3	970.3	951.0	361.5	217.7
SD	612.1	581.7	428.1	413.9	444.0	402.9	413.8	261.5	118.9
Geo. Mean	1300.3	1226.2	946.9	920.7	923.5	893.8	867.7	286.8	189.5
Geo. SD	1.6	1.6	1.6	1.6	1.6	1.6	1.6	2.2	1.8

ESRD: End stage renal disease, ESRD-STA: standard hemodialysis, ESRD-HDF: hemodiafiltration,
NRF: Normal renal function, NRF-mSTA = subjects with NRF matched to ESRD-STA, NRF-mHDF = subjects with NRF matched to ESRD-HDF, OFF = off-dialysis (period 1), ON = on-dialysis (period 2),
n.c. = not computed, BLLQ = below lower limit of quantification, Geo. = Geometric,
n = number of values >= lower limit of quantification.

Program: T14_2_1_1_plasmaC.sas (Page 16 of 20)

Table 14.2.1.1: Individual migalastat plasma concentrations [ng/mL] by time point

Pharmacokinetic Analysis Set

Group NRF-mSTA

Subject No.	Predose	0:05h	1h	2h	3h	4h	6h	8h	10h	12h	24h	48h
6	BLLQ	BLLQ	672	879	1370	1130	582	289	146	75.2	BLLQ	BLLQ
11	BLLQ	BLLQ	583	765	1290	2040	1460	802	470	283	26.0	BLLQ
12	BLLQ	BLLQ	786	1280	1480	1240	778	502	275	141	15.1	BLLQ
n	0	0	3	3	3	3	3	3	3	3	2	0
Mean	n.c.	n.c.	680.3	974.7	1380.0	1470.0	940.0	531.0	297.0	166.4	20.6	n.c.
SD	n.c.	n.c.	101.8	270.5	95.4	496.7	460.9	257.7	163.1	106.2	7.7	n.c.
Geo. Mean	n.c.	n.c.	675.3	951.2	1377.8	1419.2	871.1	488.2	266.2	144.2	19.8	n.c.

ESRD: End stage renal disease, ESRD-STA: standard hemodialysis, ESRD-HDF: hemodiafiltration, NRF: Normal renal function, NRF-mSTA = subjects with NRF matched to ESRD-STA, NRF-mHDF = subjects with NRF matched to ESRD-HDF, OFF = off-dialysis (period 1), ON = on-dialysis (period 2), n.c. = not computed, BLLQ = below lower limit of quantification, Geo. = Geometric, n = number of values >= lower limit of quantification.

Program: T14_2_1_1_plasmaC.sas (Page 17 of 20)

Table 14.2.1.1: Individual migalastat plasma concentrations [ng/mL] by time point

Pharmacokinetic Analysis Set

Group NRF-mSTA

Subject No.	Predose	0:05h	1h	2h	3h	4h	6h	8h	10h	12h	24h	48h
Geo. SD	n.c.	n.c.	1.2	1.3	1.1	1.4	1.6	1.7	1.8	1.9	1.5	n.c.

ESRD: End stage renal disease, ESRD-STA: standard hemodialysis, ESRD-HDF: hemodiafiltration,
NRF: Normal renal function, NRF-mSTA = subjects with NRF matched to ESRD-STA, NRF-mHDF = subjects with NRF matched to ESRD-HDF, OFF = off-dialysis (period 1), ON = on-dialysis (period 2),
n.c. = not computed, BLLQ = below lower limit of quantification, Geo. = Geometric,
n = number of values >= lower limit of quantification.

Program: T14_2_1_1_plasmaC.sas (Page 18 of 20)

Table 14.2.1.1: Individual migalastat plasma concentrations [ng/mL] by time point

Pharmacokinetic Analysis Set

Group NRF-mHDF

Subject No.	Predose	0:05h	1h	2h	3h	4h	6h	8h	10h	12h	24h	48h
3	BLLQ	BLLQ	1010	1180	1260	1160	586	304	165	84.8	9.28	BLLQ
4	BLLQ	BLLQ	1020	1350	2050	2070	1400	859	529	368	46.3	7.16
13	BLLQ	BLLQ	1000	1240	1430	1360	695	337	156	88.5	12.3	BLLQ
n	0	0	3	3	3	3	3	3	3	3	3	1
Mean	n.c.	n.c.	1010.0	1256.7	1580.0	1530.0	893.7	500.0	283.3	180.4	22.6	n.c.
SD	n.c.	n.c.	10.0	86.2	415.8	478.2	441.9	311.3	212.8	162.4	20.6	n.c.
Geo. Mean	n.c.	n.c.	1010.0	1254.7	1545.8	1483.6	829.2	444.8	238.8	140.3	17.4	n.c.

ESRD: End stage renal disease, ESRD-STA: standard hemodialysis, ESRD-HDF: hemodiafiltration,
NRF: Normal renal function, NRF-mSTA = subjects with NRF matched to ESRD-STA, NRF-mHDF = subjects
with NRF matched to ESRD-HDF, OFF = off-dialysis (period 1), ON = on-dialysis (period 2),
n.c. = not computed, BLLQ = below lower limit of quantification, Geo. = Geometric,
n = number of values >= lower limit of quantification.

Program: T14_2_1_1_plasmaC.sas (Page 18 of 20)

Table 14.2.1.1: Individual migalastat plasma concentrations [ng/mL] by time point

Pharmacokinetic Analysis Set

Group NRF-mHDF

Subject No.	Predose	0:05h	1h	2h	3h	4h	6h	8h	10h	12h	24h	48h
Geo. SD	n.c.	n.c.	1.0	1.1	1.3	1.3	1.6	1.8	2.0	2.3	2.4	n.c.

ESRD: End stage renal disease, ESRD-STA: standard hemodialysis, ESRD-HDF: hemodiafiltration,
NRF: Normal renal function, NRF-mSTA = subjects with NRF matched to ESRD-STA, NRF-mHDF = subjects with NRF matched to ESRD-HDF, OFF = off-dialysis (period 1), ON = on-dialysis (period 2),
n.c. = not computed, BLLQ = below lower limit of quantification, Geo. = Geometric,
n = number of values >= lower limit of quantification.

Program: T14_2_1_1_plasmaC.sas (Page 19 of 20)

Table 14.2.1.1: Individual migalastat plasma concentrations [ng/mL] by time point

Pharmacokinetic Analysis Set

Group NRF-pooled

Subject No.	Predose	0:05h	1h	2h	3h	4h	6h	8h	10h	12h	24h	48h
3	BLLQ	BLLQ	1010	1180	1260	1160	586	304	165	84.8	9.28	BLLQ
4	BLLQ	BLLQ	1020	1350	2050	2070	1400	859	529	368	46.3	7.16
6	BLLQ	BLLQ	672	879	1370	1130	582	289	146	75.2	BLLQ	BLLQ
11	BLLQ	BLLQ	583	765	1290	2040	1460	802	470	283	26.0	BLLQ
12	BLLQ	BLLQ	786	1280	1480	1240	778	502	275	141	15.1	BLLQ
13	BLLQ	BLLQ	1000	1240	1430	1360	695	337	156	88.5	12.3	BLLQ
n	0	0	6	6	6	6	6	6	6	6	5	1

ESRD: End stage renal disease, ESRD-STA: standard hemodialysis, ESRD-HDF: hemodiafiltration,
NRF: Normal renal function, NRF-mSTA = subjects with NRF matched to ESRD-STA, NRF-mHDF = subjects with NRF matched to ESRD-HDF, OFF = off-dialysis (period 1), ON = on-dialysis (period 2),
n.c. = not computed, BLLQ = below lower limit of quantification, Geo. = Geometric,
n = number of values >= lower limit of quantification.

Program: T14_2_1_1_plasmaC.sas (Page 19 of 20)

Table 14.2.1.1: Individual migalastat plasma concentrations [ng/mL] by time point

Pharmacokinetic Analysis Set

Group NRF-pooled

Subject No.	Predose	0:05h	1h	2h	3h	4h	6h	8h	10h	12h	24h	48h
Mean	n.c.	n.c.	845.2	1115.7	1480.0	1500.0	916.8	515.5	290.2	173.4	21.8	n.c.
SD	n.c.	n.c.	191.8	236.9	291.2	437.3	404.6	256.2	169.7	123.0	15.1	n.c.
Geo. Mean	n.c.	n.c.	825.8	1092.5	1459.4	1451.1	849.9	466.0	252.1	142.3	18.3	n.c.
Geo. SD	n.c.	n.c.	1.3	1.3	1.2	1.3	1.5	1.6	1.8	2.0	1.9	n.c.

ESRD: End stage renal disease, ESRD-STA: standard hemodialysis, ESRD-HDF: hemodiafiltration,
NRF: Normal renal function, NRF-mSTA = subjects with NRF matched to ESRD-STA, NRF-mHDF = subjects with NRF matched to ESRD-HDF, OFF = off-dialysis (period 1), ON = on-dialysis (period 2),
n.c. = not computed, BLLQ = below lower limit of quantification, Geo. = Geometric,
n = number of values >= lower limit of quantification.

Program: T14_2_1_1_plasmaC.sas (Page 20 of 20)

Table 14.2.1.8: Individual migalastat plasma PK characteristics

Pharmacokinetic Analysis Set

ESRD Groups, Period OFF (dialysis 24:05h to 28:05h)

Group	Subject No.	Cmax(0-24) [ng/mL]	tmax [h]	t1/2 [h]	AUC(0-t) [ng*h/mL]	AUC(0-inf) [ng*h/mL]	AUC(0-24) [ng*h/mL]	CL/F [L/h]	Vz/F [L]
ESRD-STA	5	4770	3.00		121553		85764		
	7	1610	4.00	19.20	35210	48688	26959	3.08	85.3
	10	3650	6.00	15.35	74169	97448	61349	1.54	34.1
ESRD-HDF	1	2600	4.00	31.06	59072	115051	46160	1.30	58.4
	2	1870	12.00		52914		37909		
	8	3320	6.00		76048		55216		
	n	6	6	3	6	3	6	3	3
	Mean	2970.0	5.833	21.870	69827.6	87062.2	52226.2	1.975	59.28

ESRD: End stage renal disease, NRF: Normal renal function, OFF = off-dialysis (period 1), ON = on-dialysis (period 2), n.c. = not computed, Geo. = Geometric. If >50% of the area is extrapolated, AUC(0-inf) as well as other extrapolated parameters are not reported for that profile. Cmax(0-24): Maximal observed concentration in 0-24h.

Program: T14_2_1_8_plasmaPK.sas (Page 1 of 6)

Table 14.2.1.8: Individual migalastat plasma PK characteristics

Pharmacokinetic Analysis Set

ESRD Groups, Period OFF (dialysis 24:05h to 28:05h)

Group	Subject No.	Cmax(0-24) [ng/mL]	tmax [h]	t1/2 [h]	AUC(0-t) [ng*h/mL]	AUC(0-inf) [ng*h/mL]	AUC(0-24) [ng*h/mL]	CL/F [L/h]	Vz/F [L]
	SD	1185.0	3.251	8.186	29438.7	34378.9	20480.2	0.965	25.64
	Geo. Mean	2770.8	5.241	20.919	65005.3	81726.1	48919.4	1.835	55.39
	Geo. SD	1.5	1.626	1.433	1.5	1.6	1.5	1.578	1.59

ESRD: End stage renal disease, NRF: Normal renal function, OFF = off-dialysis (period 1), ON = on-dialysis (period 2), n.c. = not computed, Geo. = Geometric. If >50% of the area is extrapolated, AUC(0-inf) as well as other extrapolated parameters are not reported for that profile. Cmax(0-24): Maximal observed concentration in 0-24h.

Program: T14_2_1_8_plasmaPK.sas (Page 2 of 6)

Table 14.2.1.8: Individual migalastat plasma PK characteristics

Pharmacokinetic Analysis Set

ESRD Groups, Period ON (dialysis 0:05h to 4:05h)

Group	Subject No.	Cmax(0-24) [ng/mL]	tmax [h]	t1/2 [h]	AUC(0-t) [ng*h/mL]	AUC(0-inf) [ng*h/mL]	AUC(0-24) [ng*h/mL]	CL/F [L/h]	Vz/F [L]
ESRD-STA	5	2500	2.00	29.19	67965	84727	36337	1.77	74.6
	7	816	6.10	20.91	26672	29118	14602	5.15	155.4
	10	1720	3.00	14.70	45147	47544	31105	3.15	66.9
ESRD-HDF	1	2620	6.02	18.69	79321	87626	46401	1.71	46.2
	2	816	10.00	25.11	33308	40661	16594	3.69	133.6
	8	1770	7.95	19.57	68668	74400	32515	2.02	56.9
	n	6	6	6	6	6	6	6	6
	Mean	1707.0	5.845	21.361	53513.6	60679.2	29592.5	2.916	88.93

ESRD: End stage renal disease, NRF: Normal renal function, OFF = off-dialysis (period 1), ON = on-dialysis (period 2), n.c. = not computed, Geo. = Geometric. If >50% of the area is extrapolated, AUC(0-inf) as well as other extrapolated parameters are not reported for that profile. Cmax(0-24): Maximal observed concentration in 0-24h.

Program: T14_2_1_8_plasmaPK.sas (Page 3 of 6)

Table 14.2.1.8: Individual migalastat plasma PK characteristics

Pharmacokinetic Analysis Set

ESRD Groups, Period ON (dialysis 0:05h to 4:05h)

Group	Subject No.	Cmax(0-24) [ng/mL]	tmax [h]	t1/2 [h]	AUC(0-t) [ng*h/mL]	AUC(0-inf) [ng*h/mL]	AUC(0-24) [ng*h/mL]	CL/F [L/h]	Vz/F [L]
	SD	781.6	2.989	5.103	21462.8	24746.8	12103.9	1.358	44.62
	Geo. Mean	1538.8	5.096	20.861	49576.6	56076.0	27291.5	2.675	80.50
	Geo. SD	1.7	1.843	1.270	1.6	1.6	1.6	1.567	1.62

ESRD: End stage renal disease, NRF: Normal renal function, OFF = off-dialysis (period 1), ON = on-dialysis (period 2), n.c. = not computed, Geo. = Geometric. If >50% of the area is extrapolated, AUC(0-inf) as well as other extrapolated parameters are not reported for that profile. Cmax(0-24): Maximal observed concentration in 0-24h.

Program: T14_2_1_8_plasmaPK.sas (Page 4 of 6)

Table 14.2.1.8: Individual migalastat plasma PK characteristics

Pharmacokinetic Analysis Set

NRF Groups

Group	Subject No.	Cmax(0-24) [ng/mL]	tmax [h]	t1/2 [h]	AUC(0-t) [ng*h/mL]	AUC(0-inf) [ng*h/mL]	AUC(0-24) [ng*h/mL]	CL/F [L/h]	Vz/F [L]
NRF-mSTA	6	1370	3.00	2.04	6698	6919	6915	21.68	63.7
	11	2040	4.00	3.40	13276	13403	13276	11.19	54.9
	12	1480	3.00	3.14	9580	9649	9580	15.55	70.4
NRF-mHDF	3	1260	3.00	3.51	7911	7958	7911	18.85	95.5
	4	2070	4.00	5.88	16568	16628	15926	9.02	76.5
	13	1430	3.00	3.96	8756	8826	8755	17.00	97.1
	n	6	6	6	6	6	6	6	6
	Mean	1608.3	3.333	3.654	10464.8	10564.0	10393.8	15.547	76.34

ESRD: End stage renal disease, NRF: Normal renal function, OFF = off-dialysis (period 1), ON = on-dialysis (period 2), n.c. = not computed, Geo. = Geometric. If >50% of the area is extrapolated, AUC(0-inf) as well as other extrapolated parameters are not reported for that profile. Cmax(0-24): Maximal observed concentration in 0-24h.

Program: T14_2_1_8_plasmaPK.sas (Page 5 of 6)

Table 14.2.1.8: Individual migalastat plasma PK characteristics

Pharmacokinetic Analysis Set

NRF Groups

Group	Subject No.	Cmax(0-24) [ng/mL]	tmax [h]	t1/2 [h]	AUC(0-t) [ng*h/mL]	AUC(0-inf) [ng*h/mL]	AUC(0-24) [ng*h/mL]	CL/F [L/h]	Vz/F [L]
	SD	353.8	0.516	1.267	3729.8	3708.6	3479.2	4.736	17.04
	Geo. Mean	1577.8	3.302	3.480	9962.5	10073.9	9949.6	14.890	74.75
	Geo. SD	1.2	1.160	1.410	1.4	1.4	1.4	1.393	1.25

ESRD: End stage renal disease, NRF: Normal renal function, OFF = off-dialysis (period 1), ON = on-dialysis (period 2), n.c. = not computed, Geo. = Geometric. If >50% of the area is extrapolated, AUC(0-inf) as well as other extrapolated parameters are not reported for that profile. Cmax(0-24): Maximal observed concentration in 0-24h.

Program: T14_2_1_8_plasmaPK.sas (Page 6 of 6)

Table 14.2.2.5: Individual migalastat PK characteristics in dialysate

Pharmacokinetic Analysis Set

ESRD Groups, Period OFF (dialysis 24:05h to 28:05h)

Group	Subject No.	VD [mL]	AeD [ug]	FeD [%]	CD [ug/mL]	P [ng/mL]	CLD [L/h]	AUC(inlet) [ng*h/mL]	AUC(outlet) [ng*h/mL]	ED [%]	QD [L/h]
ESRD-STA	5	145000	37964	25.31	0.262	1578.2	6.01	5255	1363	74.1	8.12
	7	121000	9555	6.37	0.079	384.7	6.13	1342	251	81.3	7.54
	10	124000	32673	21.78	0.263	738.9	10.83	2859	784	72.6	14.92
ESRD-HDF	1	138500	18790	12.53	0.136	635.7	7.39	2151	607	71.8	10.30
	2	146000	33891	22.59	0.232	686.3	12.19	2465	732	70.3	17.34
	8	152000	41911	27.94	0.276	1011.3	10.23	3422	890	74.0	13.83

ESRD: End stage renal disease, ESRD-STA: standard hemodialysis, ESRD-HDF: hemodiafiltration,
OFF = off-dialysis (period 1), ON = on-dialysis (period 2), n.c. = not computed, Geo. = Geometric.

Program: T14_2_2_5_diaPK.sas (Page 1 of 4)

Table 14.2.2.5: Individual migalastat PK characteristics in dialysate

Pharmacokinetic Analysis Set

ESRD Groups, Period OFF (dialysis 24:05h to 28:05h)

Group	Subject No.	VD [mL]	AeD [ug]	FeD [%]	CD [ug/mL]	P [ng/mL]	CLD [L/h]	AUC(inlet) [ng*h/mL]	AUC(outlet) [ng*h/mL]	ED [%]	QD [L/h]
	n	6	6	6	6	6	6	6	6	6	6
	Mean	137750.0	29130.7	19.420	0.2080	839.18	8.798	2915.7	771.1	74.00	12.008
	SD	12600.6	12383.5	8.256	0.0813	414.02	2.628	1341.4	364.2	3.85	3.956
	Geo. Mean	137257.8	26102.7	17.402	0.1902	763.40	8.466	2674.3	687.9	73.92	11.453
	Geo. SD	1.1	1.8	1.761	1.6563	1.61	1.358	1.6	1.8	1.05	1.406

ESRD: End stage renal disease, ESRD-STA: standard hemodialysis, ESRD-HDF: hemodiafiltration,
OFF = off-dialysis (period 1), ON = on-dialysis (period 2), n.c. = not computed, Geo. = Geometric.

Program: T14_2_2_5_diaPK.sas (Page 2 of 4)

Table 14.2.2.5: Individual migalastat PK characteristics in dialysate

Pharmacokinetic Analysis Set

ESRD Groups, Period ON (dialysis 0:05h to 4:05h)

Group	Subject No.	VD [mL]	AeD [ug]	FeD [%]	CD [ug/mL]	P [ng/mL]	CLD [L/h]	AUC(inlet) [ng*h/mL]	AUC(outlet) [ng*h/mL]	ED [%]	QD [L/h]
ESRD-STA	5	127000	68118	45.41	0.536	1803.5	9.44	7354	1694	77.0	12.27
	7	122000	26846	17.90	0.220	582.0	11.53	2453	376	84.7	13.62
	10	127000	49198	32.80	0.387	1144.5	10.61	4756	924	80.6	13.17
ESRD-HDF	1	123000	23591	15.73	0.192	886.2	6.60	4062	993	75.6	8.73
	2	155000	25613	17.08	0.165	536.8	11.93	2366	576	75.6	15.77
	8	147500	39502	26.33	0.268	802.1	12.31	3456	1005	70.9	17.36

ESRD: End stage renal disease, ESRD-STA: standard hemodialysis, ESRD-HDF: hemodiafiltration,
OFF = off-dialysis (period 1), ON = on-dialysis (period 2), n.c. = not computed, Geo. = Geometric.

Program: T14_2_2_5_diaPK.sas (Page 3 of 4)

Table 14.2.2.5: Individual migalastat PK characteristics in dialysate

Pharmacokinetic Analysis Set

ESRD Groups, Period ON (dialysis 0:05h to 4:05h)

Group	Subject No.	VD [mL]	AeD [ug]	FeD [%]	CD [ug/mL]	P [ng/mL]	CLD [L/h]	AUC(inlet) [ng*h/mL]	AUC(outlet) [ng*h/mL]	ED [%]	QD [L/h]
	n	6	6	6	6	6	6	6	6	6	6
	Mean	133583.3	38811.3	25.874	0.2948	959.18	10.405	4074.5	928.1	77.38	13.489
	SD	14037.2	17414.0	11.609	0.1419	468.59	2.130	1852.2	452.6	4.72	2.981
	Geo. Mean	132994.2	35918.6	23.946	0.2701	878.09	10.191	3765.2	835.0	77.26	13.190
	Geo. SD	1.1	1.5	1.526	1.5644	1.57	1.263	1.5	1.7	1.06	1.269

ESRD: End stage renal disease, ESRD-STA: standard hemodialysis, ESRD-HDF: hemodiafiltration,
OFF = off-dialysis (period 1), ON = on-dialysis (period 2), n.c. = not computed, Geo. = Geometric.

Program: T14_2_2_5_diaPK.sas (Page 4 of 4)

Listing 16.2.4.1: Demographic data

Safety Analysis Set

Group	Subject No.	Sex	Age [years]	Race	Weight [kg]	Height [cm]	BMI [kg/m**2]
ESRD-STA	5	F	50	White	75.6	155	31.5
	7	M	55	White	82.5	179	25.7
	10	M	39	White	70.6	175	23.1
ESRD-HDF	1	F	40	White	74.5	154	31.4
	2	F	64	White	76.7	170	26.5
	8	F	37	White	56.0	166	20.3
NRF-mSTA	6	F	46	White	82.0	155	34.1
	11	M	57	White	79.1	172	26.7
	12	M	41	White	66.0	173	22.1
NRF-mHDF	3	F	42	White	67.8	160	26.5

F = Female M = male. Measurements refer to the screening visit.
ESRD: End stage renal disease, ESRD-STA: standard hemodialysis, ESRD-HDF: hemodiafiltration
NRF: Normal renal function, NRF-mSTA = subjects with NRF matched to ESRD-STA, NRF-mHDF = subjects with NRF matched to ESRD-HDF

Program: L16_2_4_1.sas (Page 1 of 2)

Listing 16.2.4.1: Demographic data

Safety Analysis Set

Group	Subject No.	Sex	Age [years]	Race	Weight [kg]	Height [cm]	BMI [kg/m**2]
NRF-mHDF	4	F	69	White	67.2	161	25.9
	13	F	33	White	59.6	167	21.4

F = Female M = male. Measurements refer to the screening visit.
ESRD: End stage renal disease, ESRD-STA: standard hemodialysis, ESRD-HDF: hemodiafiltration
NRF: Normal renal function, NRF-mSTA = subjects with NRF matched to ESRD-STA, NRF-mHDF = subjects with NRF matched to ESRD-HDF

Program: L16_2_4_1.sas (Page 2 of 2)

Listing 16.2.4.2: Estimated glomerular filtration rate at screening

Safety Analysis Set

Group	Subject No.	Serum Creatinine [mg/dL]	Age [years]	Sex	Race	eGFR [mL/min/1.73m2]	Date/Time of Specimen Collection [YYYY-MM-DDTHH:MM]
ESRD-STA	5	6.84	50	F	White	6.4	2019-07-18T10:09
	7	4.52	55	M	White	13.6	2019-10-10T10:15
	10	2.76	39	M	White	25.8	2019-11-14T11:20
		5.26	39	M	White	12.2	2019-11-20T08:20
ESRD-HDF	1	10.69	40	F	White	4.0	2019-06-05T10:48
	2	7.66	64	F	White	5.3	2019-06-19T10:50

F = Female, M = Male.
ESRD: End stage renal disease, ESRD-STA: standard hemodialysis, ESRD-HDF: hemodiafiltration
NRF: Normal renal function, NRF-mSTA = subjects with NRF matched to ESRD-STA, NRF-mHDF = subjects with NRF matched to ESRD-HDF
eGFR = estimated Glomerular Filtration Rate, calculated based on the Modification of Diet in Renal Disease (MDRD) equation

Program: L16_2_4_2.sas (Page 1 of 3)

Listing 16.2.4.2: Estimated glomerular filtration rate at screening

Safety Analysis Set

Group	Subject No.	Serum Creatinine [mg/dL]	Age [years]	Sex	Race	eGFR [mL/min/1.73m2]	Date/Time of Specimen Collection [YYYY-MM-DDTHH:MM]
ESRD-HDF	8	5.07	37	F	White	9.6	2019-11-05T09:58
NRF-mSTA	6	0.40	46	F	White	171.8	2019-08-16T09:27
	11	0.75	57	M	White	107.3	2019-11-18T09:40
	12	0.63	41	M	White	140.3	2019-12-06T10:05
NRF-mHDF	3	0.71	42	F	White	90.3	2019-06-21T09:33
		0.68	42	F	White	94.9	2019-06-25T08:48

F = Female, M = Male.
ESRD: End stage renal disease, ESRD-STA: standard hemodialysis, ESRD-HDF: hemodiafiltration
NRF: Normal renal function, NRF-mSTA = subjects with NRF matched to ESRD-STA, NRF-mHDF = subjects with NRF matched to ESRD-HDF
eGFR = estimated Glomerular Filtration Rate, calculated based on the Modification of Diet in Renal Disease (MDRD) equation

Program: L16_2_4_2.sas (Page 2 of 3)

Listing 16.2.4.2: Estimated glomerular filtration rate at screening

Safety Analysis Set

Group	Subject No.	Serum Creatinine [mg/dL]	Age [years]	Sex	Race	eGFR [mL/min/1.73m2]	Date/Time of Specimen Collection [YYYY-MM-DDTHH:MM]
NRF-mHDF	4	0.66	69	F	White	88.8	2019-07-18T10:35
	13	0.74	33	F	White	90.4	2019-12-09T09:10

F = Female, M = Male.
ESRD: End stage renal disease, ESRD-STA: standard hemodialysis, ESRD-HDF: hemodiafiltration
NRF: Normal renal function, NRF-mSTA = subjects with NRF matched to ESRD-STA, NRF-mHDF = subjects with NRF matched to ESRD-HDF
eGFR = estimated Glomerular Filtration Rate, calculated based on the Modification of Diet in Renal Disease (MDRD) equation

Program: L16_2_4_2.sas (Page 3 of 3)

Listing 16.2.5.5: PK characteristics of migalastat in urine

Pharmacokinetic Analysis Set

Group	Subj. No.	Period	Ae [ng]	Fe [%]	CLr [L/h]
ESRD-STA	5	OFF	2098510	1.399	0.017
		ON	123721	0.0825	0.002
	7	OFF	9382676	6.2551	0.266
		ON	6027136	4.0181	0.226
	10	OFF	20269600	13.5131	0.273
		ON	8917974	5.9453	0.198

ESRD: End stage renal disease, ESRD-STA: standard hemodialysis, ESRD-HDF: hemodiafiltration
NRF: Normal renal function, NRF-mSTA = subjects with normal renal function matched to ESRD-STA,
NRF-mHDF = subjects with normal renal function matched to ESRD-HDF; OFF = off-dialysis (period 1),
ON = on-dialysis (period 2)
Ae = total amount excreted, Fe = fraction of dose recovered in urine, CLr = renal clearance.

Program: L16_2_5_5.sas (Page 1 of 3)

Listing 16.2.5.5: PK characteristics of migalastat in urine

Pharmacokinetic Analysis Set

Group	Subj. No.	Period	Ae [ng]	Fe [%]	CLr [L/h]
ESRD-HDF	1	OFF	3834430	2.5563	0.065
		ON	655860	0.4372	0.008
	2	OFF	1347670	0.8984	0.025
		ON	845458	0.5636	0.025
	8	OFF	5350156	3.5668	0.070
		ON	2857110	1.9047	0.042

ESRD: End stage renal disease, ESRD-STA: standard hemodialysis, ESRD-HDF: hemodiafiltration
NRF: Normal renal function, NRF-mSTA = subjects with normal renal function matched to ESRD-STA,
NRF-mHDF = subjects with normal renal function matched to ESRD-HDF; OFF = off-dialysis (period 1),
ON = on-dialysis (period 2)
Ae = total amount excreted, Fe = fraction of dose recovered in urine, CLr = renal clearance.

Program: L16_2_5_5.sas (Page 2 of 3)

Listing 16.2.5.5: PK characteristics of migalastat in urine

Pharmacokinetic Analysis Set

Group	Subj. No.	Period	Ae [ng]	Fe [%]	CLr [L/h]
NRF-mSTA	6		35801355	23.8676	5.345
	11		65421528	43.6144	4.928
	12		67276778	44.8512	7.022
NRF-mHDF	3		44618810	29.7459	5.640
	4		83014520	55.343	5.011
	13		53422710	35.6151	6.101

ESRD: End stage renal disease, ESRD-STA: standard hemodialysis, ESRD-HDF: hemodiafiltration
NRF: Normal renal function, NRF-mSTA = subjects with normal renal function matched to ESRD-STA,
NRF-mHDF = subjects with normal renal function matched to ESRD-HDF; OFF = off-dialysis (period 1),
ON = on-dialysis (period 2)
Ae = total amount excreted, Fe = fraction of dose recovered in urine, CLr = renal clearance.

Program: L16_2_5_5.sas (Page 3 of 3)

Listing 16.2.7.2: Treatment emergent adverse events

Safety Analysis Set

Group	Subj. No.	Pe-riod	AE No.	Reported term	Preferred term	System organ class	Start Date/Time [YYYY-MM-DDT HH:MM]	Time since IMP admin. [DDTHH:MM]	On-going	End date/time [YYYY-MM-DDT HH:MM]
ESRD-STA	5	OFF	1	Dizziness	Dizziness	Nervous system disorders	2019-07-24T08:35	01T00:35:00	N	2019-07-24T13:40
		ON	2	Dizziness	Dizziness	Nervous system disorders	2019-08-01T08:40	01T00:40:00	N	2019-08-01T14:00
ESRD-HDF	1	ON	1	Headache	Headache	Nervous system disorders	2019-06-20T17:30	01T09:29:00	N	2019-06-20T23:00

ESRD: End stage renal disease, ESRD-STA: standard hemodialysis, ESRD-HDF: hemodiafiltration, NRF: Normal renal function, NRF-mSTA = subjects with NRF matched to ESRD-STA, NRF-mHDF = subjects with NRF matched to ESRD-HDF, OFF = off-dialysis (period 1), ON = on-dialysis (period 2), admin. = administration, SAE = Serious Adverse Event, Y = Yes, N = No, Causality = Reasonable causal relationship to IMP.

Program: L16_2_7_2.sas (Page 1 of 6)

Listing 16.2.7.2: Treatment emergent adverse events

Safety Analysis Set

Group	Subj. No.	Pe-riod	AE No.	Reported term	Preferred term	System organ class	Start Date/Time [YYYY-MM-DDT HH:MM]	Time since IMP admin. [DDTHH:MM]	On-going	End date/time [YYYY-MM-DDT HH:MM]
ESRD-HDF	2		1	Pain at shunt punction site, left	Arteriovenous fistula site complication	Injury, poisoning and procedural complications	2019-07-04T08:14	09T00:14:00	N	2019-07-04T10:00
		ON	2	Panic attack	Panic attack	Psychiatric disorders	2019-07-04T08:46	01T00:31:00	N	2019-07-04T09:00
	8	OFF	1	Headache	Headache	Nervous system disorders	2019-11-14T14:58	02T06:28:00	N	2019-11-14T20:00

ESRD: End stage renal disease, ESRD-STA: standard hemodialysis, ESRD-HDF: hemodiafiltration, NRF: Normal renal function, NRF-mSTA = subjects with NRF matched to ESRD-STA, NRF-mHDF = subjects with NRF matched to ESRD-HDF, OFF = off-dialysis (period 1), ON = on-dialysis (period 2), admin. = administration, SAE = Serious Adverse Event, Y = Yes, N = No, Causality = Reasonable causal relationship to IMP.

Program: L16_2_7_2.sas (Page 2 of 6)

Listing 16.2.7.2: Treatment emergent adverse events

Safety Analysis Set

Group	Subj. No.	Pe-riod	AE No.	Reported term	Preferred term	System organ class	Start Date/Time [YYYY-MM-DDT HH:MM]	Time since IMP admin. [DDTHH:MM]	On-going	End date/time [YYYY-MM-DDT HH:MM]
NRF-mHDF	3		1	Headache	Headache	Nervous system disorders	2019-07-02T21:00	01T13:00:00	N	2019-07-03T06:00
	4		1	Headache	Headache	Nervous system disorders	2019-07-23T19:05	01T11:05:00	N	2019-07-24T15:00

ESRD: End stage renal disease, ESRD-STA: standard hemodialysis, ESRD-HDF: hemodiafiltration, NRF: Normal renal function, NRF-mSTA = subjects with NRF matched to ESRD-STA, NRF-mHDF = subjects with NRF matched to ESRD-HDF, OFF = off-dialysis (period 1), ON = on-dialysis (period 2), admin. = administration, SAE = Serious Adverse Event, Y = Yes, N = No, Causality = Reasonable causal relationship to IMP.

Program: L16_2_7_2.sas (Page 3 of 6)

Listing 16.2.7.2: Treatment emergent adverse events

Safety Analysis Set

Group	Subj. No.	Pe-riod	AE No.	Reported term	SAE	SAE Rea-son	Se-verity	Causal Relationship to Study Drug	Action taken with study treatment	Remedial drug therapy	Other specific treatment (s)	Outcome of AE	Comment
ESRD-STA	5	OFF	1	Dizziness	N		MILD	POSSIBLE	NOT APPLICABLE	N	N	RECOVERED / RESOLVED	
		ON	2	Dizziness	N		MILD	POSSIBLE	NOT APPLICABLE	N	N	RECOVERED / RESOLVED	
ESRD-HDF	1	ON	1	Headache	N		MILD	POSSIBLE	NOT APPLICABLE	Y	N	RECOVERED / RESOLVED	

ESRD: End stage renal disease, ESRD-STA: standard hemodialysis, ESRD-HDF: hemodiafiltration, NRF: Normal renal function, NRF-mSTA = subjects with NRF matched to ESRD-STA, NRF-mHDF = subjects with NRF matched to ESRD-HDF, OFF = off-dialysis (period 1), ON = on-dialysis (period 2), admin. = administration, SAE = Serious Adverse Event, Y = Yes, N = No, Causality = Reasonable causal relationship to IMP.

Program: L16_2_7_2.sas (Page 4 of 6)

Listing 16.2.7.2: Treatment emergent adverse events

Safety Analysis Set

Group	Subj. No.	Pe-riod	AE No.	Reported term	SAE	SAE Rea-son	Se-verity	Causal Relationship to Study Drug	Action taken with study treatment	Remedial drug therapy	Other specific treatment (s)	Outcome of AE	Comment
ESRD-HDF	2		1	Pain at shunt punction site, left	N		MODERATE	UNRELATED	DOSE NOT CHANGED	N	N	RECOVERED / RESOLVED	due to pain during shunt punction
		ON	2	Panic attack	N		MILD	UNLIKELY	NOT APPLICABLE	Y	N	RECOVERED / RESOLVED	
	8	OFF	1	Headache	N		MILD	UNLIKELY	DOSE NOT CHANGED	Y	N	RECOVERED / RESOLVED	

ESRD: End stage renal disease, ESRD-STA: standard hemodialysis, ESRD-HDF: hemodiafiltration, NRF: Normal renal function, NRF-mSTA = subjects with NRF matched to ESRD-STA, NRF-mHDF = subjects with NRF matched to ESRD-HDF, OFF = off-dialysis (period 1), ON = on-dialysis (period 2), admin. = administration, SAE = Serious Adverse Event, Y = Yes, N = No, Causality = Reasonable causal relationship to IMP.

Program: L16_2_7_2.sas (Page 5 of 6)

Listing 16.2.7.2: Treatment emergent adverse events

Safety Analysis Set

Group	Subj. No.	Pe- riod	AE No.	Reported term	SAE	SAE Rea- son	Se- verity	Causal Relationship to Study Drug	Action taken with study treatment	Remedial drug therapy	Other specific treatment (s)	Outcome of AE	Comment
NRF-mHDF	3		1	Headache	N		MILD	POSSIBLE	NOT APPLICABLE	Y	N	RECOVERED / RESOLVED	
	4		1	Headache	N		MILD	POSSIBLE	NOT APPLICABLE	N	N	RECOVERED / RESOLVED	

ESRD: End stage renal disease, ESRD-STA: standard hemodialysis, ESRD-HDF: hemodiafiltration, NRF: Normal renal function, NRF-mSTA = subjects with NRF matched to ESRD-STA, NRF-mHDF = subjects with NRF matched to ESRD-HDF, OFF = off-dialysis (period 1), ON = on-dialysis (period 2), admin. = administration, SAE = Serious Adverse Event, Y = Yes, N = No, Causality = Reasonable causal relationship to IMP.

Program: L16_2_7_2.sas (Page 6 of 6)
