# Supplementary material for: Pharmacokinetic evaluation of single-dose migalastat in non-Fabry disease subjects with ESRD receiving dialysis treatment, and use of modeling to select dose regimens in Fabry disease subjects with ESRD receiving dialysis treatment
Source: PLoS One. 2024 Dec 5;19(12):e0314030. doi: 10.1371/journal.pone.0314030 (PMC11620666; doi:10.1371/journal.pone.0314030)
Supplement: S2 Dataset — (PDF) [file pone.0314030.s008.pdf]

Simulation Summary Report

Supplemental Report on the Evaluation of the Optimal Dose of Migalastat in Fabry Patients on Dialysis Based on Data from an Open-Label Study in Non-Fabry Subjects with End-Stage Renal Disease Receiving Hemodialysis or Hemodiafiltration Matched to Healthy Subjects

**Name of Investigational Drug:**	Migalastat
**Study Number:**	AT1001-035
**Sponsor:**	Amicus Therapeutics, Inc.<br>1 Cedar Brook Drive<br>Cranbury, NJ 08512<br>Phone: 609-662-2000
**Pharmacokinetic Analysis Site:**	Nuventra, Inc.<br>2525 Meridian Parkway, Suite 200<br>Durham, NC 27713
**Report Number:**	2158-RPT002
**Phase:**	1
**Version:**	Final
**Date:**	07 DEC 2021
**Description**	This supplemental report for Study AT1001-035 includes additional analysis conducted that were not reported in the CSR

Scenario	Dialysis Frequency	Migalastat Regimen	Bioequivalent?		% of Subjects	
			C_{avg}	C_{max}	C_{max} >10 µM	C_{trough} BLQ
Pre-specified Scenarios						
1	N/A (control)	123 mg QOD	Reference	Reference	17%	44%
2	Q3D	123 mg QOD	High	High	59-77%	0%
3	QOD	123 mg QOD	High	High	82%	0%
4	QOD	123 mg Q4D	High	High	65%	0%
5	Q3D	123 mg Q3D	High	High	71%	0%
Extended Dosing Interval Scenarios						
6	QOD	49 mg Q4D	Slightly high	Low	1%	0%
7	QOD	65 mg Q6D	**Yes**	Slightly low	4%	4%
8	QOD	123 mg Q12D	**Yes**	High	56%	79%
9	Q3D	82 mg Q6D	High	**Yes**	14%	0%
10	Q3D	123 mg Q12D	**Yes**	High	59%	60%
Week-Based Dosing and Dialysis Schedule						
11	MWF QW	82 mg QW	**Yes**	**Yes**	15%	8%
12	MWF QW	123 mg Q2W	**Yes**	High	56%	92%
13	MTh QW	82 mg QW	Slightly high	**Yes**	12%	7%
14	MTh QW	82 mg Q2W	Low	**Yes**	8%	90%
15	MTh QW	123 mg Q2W	**Yes**	High	49%	78%
20[a]	MWF QW	123 mg QW	High	High	53%	2%
21[a]	MTh QW	123 mg QW	High	High	58%	1%
Twice Every Other Week Dosing and Week-Based Dialysis Schedule						
16	MTh QW	82 mg SuW Q2W	**Yes**	**Yes**	12%	56%
17	MWF QW	82 mg SuW Q2W	**Yes**	**Yes**	15%	62%
18	MTh QW	82 mg SuTu Q2W	Slightly high	**Yes**	17%	54%
19	MWF QW	82 mg SuTu Q2W	**Yes**	**Yes**	11%	75%

ESRD = end-stage renal disease; MWF = Monday, Wednesday, Friday; MTh = Monday, Thursday; Q3D = every 3 days; Q4D = every 4 days; Q6D = every 6 days; Q12D = every 12 days; QOD = every other day; Q2W = every other week; QW = every week; SuTu = Sunday, Tuesday; SuW = Sunday, Wednesday.

[a] These simulations were added after the twice every other week dosing simulations were conducted.

Table 1. Summary of Previous PopPK Model of Migalastat

Parameter	Equation	Label	Estimate (RSE) [Shrinkage]	
			Fixed Effect	IIV or Residual
Ka (h^{-1})	$Ka = max\begin{cases} 0.00001 \\ \theta_1 \times \exp(\eta_1) + (\theta_2 \times \exp(\eta_2) \times TADCO) \end{cases}$	1. Intercept on Ka	0.256 (9%)	60.4% (11%) [30%]
		2. Slope for time-dependent effect on Ka (with a maximum time of 24 h)	0.284 (9%)	60.7% (9%) [31%]
ALAG1 (h)	$ALAG1 = \theta_3$	3. Lag time	0.175 (5%)	–
F1	$F1 = \theta_4$	4. Bioavailability	1 FIX	–
CL/F (L/h)	$\frac{CL}{F} = if\left(eGFR > 120, \theta_5, \theta_6 \times \frac{eGFR}{90}\right)^{\theta_7} \times (1 + \theta_8 \times (1 - FABRY)) \times WTCO^{\theta_9} \times \exp(\eta_5)$	5. Coefficient for eGFR effect on CL/F for Fabry subjects with eGFR >120 mL/min/1.73 m^2 and weight ≥70 kg	20.9 (17%)	28.8% (7%) [4%]
		6. Coefficient for eGFR effect on CL/F for Fabry subjects with eGFR =90 mL/min/1.73 m^2 and weight ≥70 kg	18.6 (16%)	–
		7. Exponent for eGFR effect on CL/F	0.922 (6%)	–
		8. Fractional change in CL/F in subjects without Fabry disease	-0.15 (25%)	–
		9. Exponent for weight effect on CL/F and Q/F	0.75 FIX	–
Q/F (L/h)	$\frac{Q}{F} = \theta_{10} \times WTCO^{\theta_9}$	10. Q/F for subjects with weight ≥70 kg	1 (5%)	–
V_2/F (L)	$\frac{V_2}{F} = \theta_{11} \times (1 + \theta_{12} \times (1 - FABRY)) \times WTCO^{\theta_{13}} \times \exp(\eta_{11})$	11. Typical value for V_2/F for Fabry subjects with weight ≥70 kg	70.1 (5%)	34.5% (6%) [7%]
		12. Fractional change in V_2/F in subjects without Fabry disease	-0.306 (13%)	–
		13. Exponent for weight effect on V_2/F and V_3/F	1 FIX	–
V_3/F (L)	$\frac{V_3}{F} = \theta_{14} \times WTCO^{\theta_{13}}$	14. V_3/F for Fabry subjects with weight ≥70 kg	27.5 (12%)	–
Residual Error	Combined proportional and additive residual error	Proportional (%)	–	26.2% (5.5%) [7%]
		Additive (ng/mL)	–	2.55 (30%) [7%]

FABRY = Fabry disease status, 0 = no Fabry disease, 1 = Fabry disease; FIX = parameter was fixed and not estimated; TADCO = time after dose capped at 24 h; WTCO = weight capped at and normalized to 70 kg.

Table 4. Parameters for the Migalastat PBPK Models in Humans

Parameter	Full Dataset Model	Steady-State Dataset Model
Basic Physicochemical Properties		
Is it a small molecule?	Yes	
Lipophilicity	-1.70	-3.03
Fraction unbound	100%	
Molecular weight (g/mol)	163.17	
Has halogens	No	
Compound type	Monoprotic base	
pKa (Basic)	7.47	
Solubility	500 g/L between pH 1.2 to 7.5	
Partition coefficient calculation method	Schmitt	
Cellular permeabilities calculation method	PK-Sim Standard	
Biological Properties		
Specific intestinal permeability (cm/min)	1×10^{-4}	2×10^{-5}
Specific binding – Lysosome: K_d (M)	0.01	0.01
Specific binding – Lysosome: K_{off} (min^{-1})	1.6×10^{-3}	2×10^{-3}
Hepatic clearance – specific clearance (min^{-1})	0.05	0.04
Extrahepatic clearance – UDPGT in brain CLspec/[enzyme] (L/µmol/min)	2.10×10^{-3}	2.10×10^{-3}
Tissue Partition Coefficient (Intracellular:Plasma)		
Heart	0.01	5×10^{-3}
Kidney	4.00	0.76
Liver	0.10	5×10^{-3}
Muscle	0.50	0.79
Skin	0.54	0.54
Brain	0.81	0.80

4.4.2. Adaptation of Models for Use in Subjects with Impaired Clearance

Migalastat PBPK models were modified to be usable for predicting exposures in healthy subjects and ESRD patients. Originally, extra-renal clearance (a minor elimination pathway) was mostly accounted for by an overall hepatic clearance process, which was fitted using plasma concentration data from healthy subjects and was not dependent on characteristics of the individual. Characterization of hepatic clearance was changed from an overall clearance process to a theoretical enzyme metabolism process located in the liver, such that the degree of extra-renal clearance impairment may be controlled by changing the theoretical expression level of hepatic enzymes in the individual virtual subject, while the predictions in the individual healthy subject remained unaltered. Hepatic metabolism was assumed to be mediated by the same enzyme as in the brain from the original PBPK model, uridine diphosphate glucuronosyltransferase (UDPGT). Thus, the same drug model was used for healthy subjects and ESRD patients, but healthy subjects and ESRD patients may differ in their expression of the hepatic UDPGT enzyme and have different capacity for metabolism.

4.4.3. Creation of an ESRD Virtual Subject

A virtual ESRD subject was created from the established virtual healthy subject by fitting glomerular filtration rate (GFR) and theoretical hepatic enzyme expression level to the observed plasma concentration-time data of ESRD patients in Study AT1001-035. The

The QW and Q2W regimens with typical dialysis schedules were equally adequate with different advantages and disadvantages:

- 82 mg QW dosing: Scenarios 11 (dialysis 3 times a week / 82 mg QW) and 13 (dialysis 2 times a week / 82 mg QW) result in bioequivalent or nearly bioequivalent C_{avg} and C_{max}. However, few subjects have a BLQ C_{trough} (which is a desired characteristic to enable migalastat dissociation from α-Gal A).
- 123 mg Q2W dosing: Scenarios 12 (dialysis 3 times a week / 123 mg Q2W) and 15 (dialysis 2 times a week / 123 mg Q2W) both result in bioequivalent C_{avg}, elevated C_{max} that is within 2-fold above the bioequivalent range, and a large proportion of subjects with C_{max} above 10 µM and/or BLQ C_{trough}.
- 82 mg Q2W dosing: Scenario 14 (dialysis 2 times a week / 82 mg Q2W) resulted in suboptimal C_{avg} and bioequivalent C_{max}.

Figure 6. Geometric Mean Ratios for Migalastat C_{avg} and C_{max} for Week-Based Dosing and Dialysis Schedule

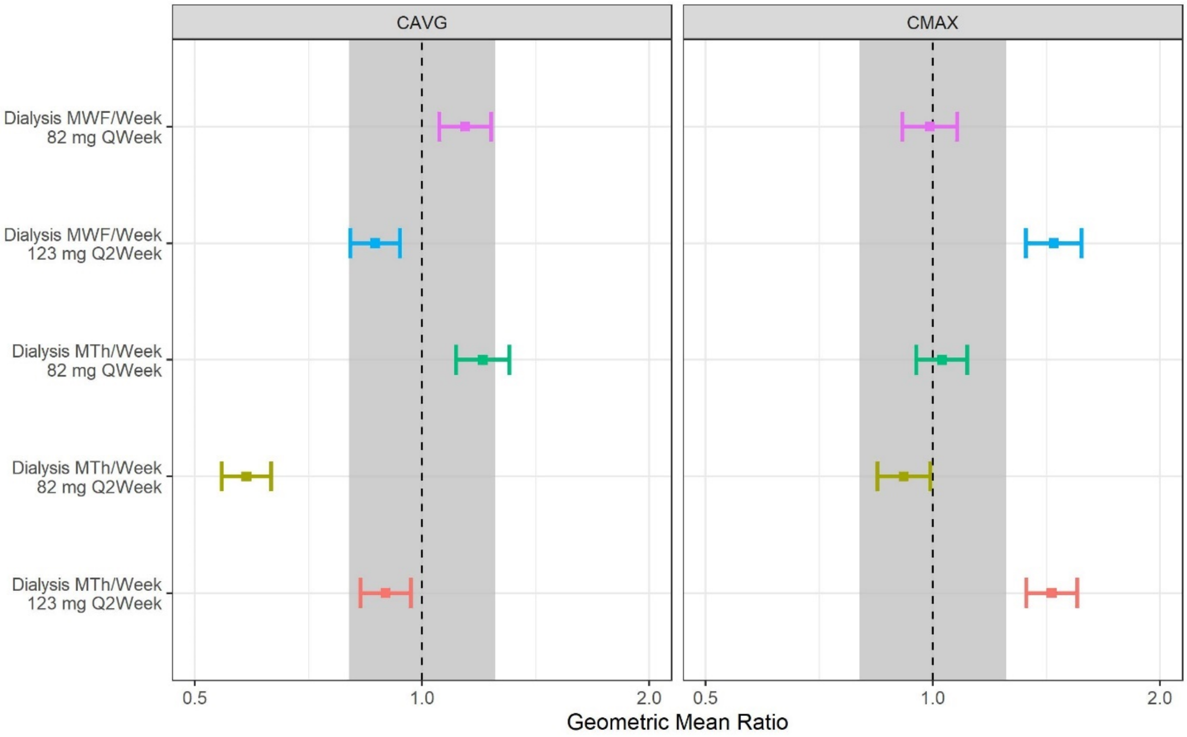

Black dashed line = Geometric Mean Ratio of 1.0; Gray shaded region = Bioequivalence criteria for 0.8 to 1.25 for the 90% CI of the geometric mean ratio. Colored dot and segment = Geometric mean ratio and 90% CI.

Scenarios 11 (dialysis 3 times a week / 82 mg QW) and 13 (dialysis 2 times a week / 82 mg QW) produce nearly bioequivalent exposures, but the proportion of virtual subjects attaining C_{max} >10 µM and BLQ C_{trough} is low. Therefore, more complicated dosing regimens to administer 82 mg of migalastat twice 48 or 72 hours apart on alternating weeks were explored with the goals of raising C_{max} without exceeding the bioequivalent range and decreasing C_{trough} to BLQ levels.

After completion of these simulations, predictions for a higher dose weekly regimen (migalastat 123 mg QW) were conducted by scaling from Scenarios 11 and 13 assuming dose

Table 7. Predicted Fraction of Dosing Interval Above EC50 in the Tissues for Typical Male Subject with Weight of 73 kg

Dosing[a]	Dialysis	Brain	Heart	Kidney	Liver	Skin	Small Intestine
Full Dataset Model							
123 mg QOD in healthy subject	None	0%	5.00%	40.1%	14.5%	19.9%	25.2%
123 mg Q2W	2x/Week	0%	6.77%	29.0%	12.9%	17.3%	21.9%
	3x/Week	0%	6.77%	27.2%	12.9%	17.3%	21.3%
82 mg QW	2x/Week	0%	12.8%	56.8%	19.2%	27.4%	35.4%
	3x/Week	0%	12.8%	47.9%	19.2%	27.4%	35.4%
123 mg QW	2x/Week	0%	13.5%	58.0%	25.7%	34.7%	43.8%
	3x/Week	0%	13.5%	54.5%	25.7%	34.5%	42.6%
82 mg twice 48 h apart Q2W[b]	2x/Week	0%	13.4%	46.2%	23.4%	30.5%	33.1%
	3x/Week	0%	12.8%	38.5%	19.8%	28.1%	33.1%
82 mg twice 72 h apart Q2W[b]	2x/Week	0%	12.8%	52.8%	19.2%	27.4%	35.9%
	3x/Week	0%	6.05%	46.8%	20.4%	27.4%	32.3%
Steady-State Dataset Model							
123 mg QOD in healthy subject	None	0%	7.71%	35.0%	11.0%	–[c]	22.5%
123 mg Q2W	2x/Week	0%	7.59%	41.5%	11.5%	–[c]	24.6%
	3x/Week	0%	7.59%	35.9%	11.5%	–[c]	21.4%
82 mg QW	2x/Week	0%	13.8%	67.0%	15.8%	–[c]	38.5%
	3x/Week	0%	13.8%	71.1%	15.8%	–[c]	38.5%
123 mg QW	2x/Week	0%	15.2%	84.2%	23.5%	–[c]	49.9%
	3x/Week	0%	15.2%	72.3%	23.5%	–[c]	43.3%
82 mg twice 48 h apart Q2W[b]	2x/Week	0%	15.7%	57.0%	21.1%	–[c]	36.8%
	3x/Week	0%	13.9%	54.7%	17.0%	–[c]	35.6%
82 mg twice 72 h apart Q2W[b]	2x/Week	0%	13.9%	57.0%	16.4%	–[c]	40.3%
	3x/Week	0%	11.5%	55.3%	15.2%	–[c]	33.1%

QOD = every other day; Q2W = every other week.
[a] Unless otherwise noted, subjects were assumed to be an ESRD patient.
[b] Dosing interval for twice Q2W dosing regimens was assessed as 2 weeks.
[c] Skin tissue exposure was only observed in the single-dose mouse biodistribution study, so Steady-State Dataset Model was not built to predict skin exposure.

Appendix Figure 7. Median (95% Prediction Interval) Steady-State Concentration-Time Profiles for Week-Based Dosing and Dialysis Schedule Scenarios

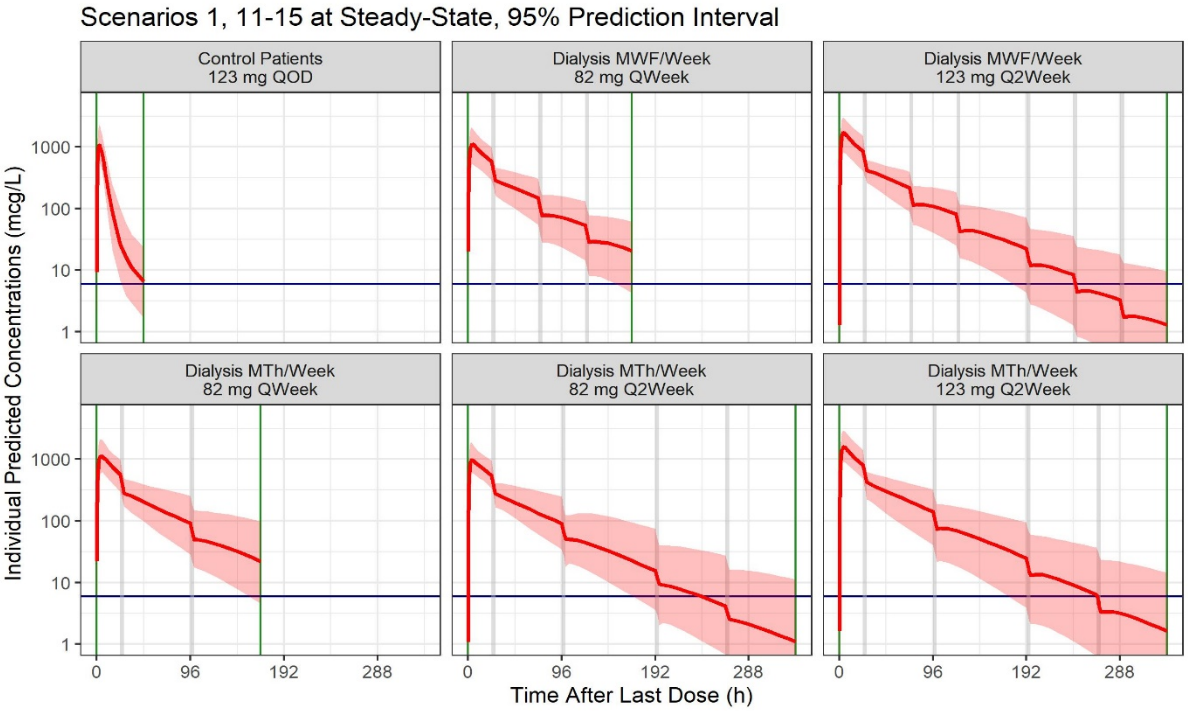

Red area = 2.5th to 97.5th prediction interval; red line = median; blue line = LLOQ; green line = migalastat dose; gray area = dialysis period.

Appendix Table 7. Summary of Predicted PK Parameters for Week-Based Dosing and Dialysis Schedule Scenarios

Scenario	Dialysis	Dose	C_{max} (ng/mL)*	C_{avg} (ng/mL)*	%C_{max} > 10 µM	%C_{trough} ≤LLOQ**
11	MWF QW	82 mg QW	1120 (1050, 1190)	208 (196, 219)	15%	8%
12	MWF QW	123 mg Q2W	1630 (1540, 1740)	158 (150, 166)	56%	92%
13	MTh QW	82 mg QW	1160 (1100, 1220)	219 (207, 232)	12%	7%
14	MTh QW	82 mg Q2W	1030 (979, 1090)	106 (101, 112)	8%	90%
15	MTh QW	123 mg Q2W	1620 (1540, 1710)	163 (155, 171)	49%	78%

*Presented numbers are geometric mean (90% CI)
**LLOQ = lower limit of quantification or 5.88 ng/mL
