# Supplementary material for: Pharmacokinetic evaluation of single-dose migalastat in non-Fabry disease subjects with ESRD receiving dialysis treatment, and use of modeling to select dose regimens in Fabry disease subjects with ESRD receiving dialysis treatment
Source: PLoS One. 2024 Dec 5;19(12):e0314030. doi: 10.1371/journal.pone.0314030 (PMC11620666; doi:10.1371/journal.pone.0314030)
Supplement: S1 Protocol — (PDF) [file pone.0314030.s009.pdf]

Integrated Clinical Study Protocol

An open-label study to evaluate the pharmacokinetics and safety of migalastat in non-Fabry end-stage renal disease subjects receiving dialysis and matched healthy subjects

**Protocol Version:**	Final 3, 29 Aug 2019<br>This protocol version is an integration of protocol version Final 2 dated 10 Apr 2019 and Amendment 2 dated 29 Aug 2019.
**Previous Version:**	Final 2, 10 Apr 2019<br>This protocol version is an integration of the original protocol (Final dated 21 Dec 2018) and Amendment 1 dated 10 Apr 2019.<br>Final, 21 Dec 2018
**Study Numbers:**	CRS Study No.: 090/18-03.MT<br>Sponsor Study No.: AT1001-035<br>EudraCT No.: 2018-003684-57<br>US IND No.: 68,456
**Test drug:**	AT1001 (migalastat hydrochloride)
**Clinical study phase:**	1
**Sponsor:**	Amicus Therapeutics, Inc.<br>1 Cedar Brook Drive<br>Cranbury, NJ 08512<br>Phone: +1 609-662-2000
**Legal representative in the European Union**	Amicus Therapeutics Europe Limited<br>Block 1[1]<br>Corporate Park, Ballycoolen Road<br>Blanchardstown, Dublin, D15 AKK1 Ireland
**GCP-compliance:**	The investigation will be carried out in accordance with the study protocol, the ethical principles that have their origin in the current accepted version of the Declaration of Helsinki and in accordance with Good Clinical Practice Guideline and applicable local laws and regulations. Essential documents will be archived in accordance with Good Clinical Practice Guideline.

[redacted]

[1] Address of legal representative in the European Union changed via Amendment 1

1 Protocol approval

Signature sponsor

The signatory agrees to the content of the final clinical study protocol as presented.

[redacted]

Sr. Director, Clinical Research

Signature investigator

The signatory agrees to the content of the final clinical study protocol as presented.

[redacted]

Investigator

2 Synopsis - amended[2]

**Study title:**	An open-label study to evaluate the pharmacokinetics and safety of migalastat in non-Fabry end-stage renal disease subjects receiving dialysis and matched healthy subjects
**Clinical study phase:**	1
**Study objectives:**	Primary objective:<br>• To characterize the pharmacokinetics (PK) of migalastat in non-Fabry end-stage renal disease (ESRD) subjects who are receiving hemodialysis treatment (standard hemodialysis or hemodiafiltration)<br>Secondary objective:<br>• To assess the safety and tolerability of migalastat in non-Fabry ESRD subjects who are receiving hemodialysis treatment (standard hemodialysis or hemodiafiltration)
**Test drug:**	Name: migalastat hydrochloride (HCl)<br>Active ingredient: migalastat<br>Formulation: capsule<br>Dose: 150 mg migalastat HCl (equivalent to 123 mg migalastat free base)<br>Route of administration: oral (subjects will be fasted overnight and until 2 h after administration of migalastat)<br>Duration of treatment: individual ESRD subjects will receive 2 single doses, administered at least 8 days apart; individual healthy subjects will receive 1 dose
**Indication:**	Migalastat is for the treatment of Fabry disease. Subjects in this study are non-Fabry subjects; for each, their personal and familial medical history will be reviewed as not indicative of Fabry disease.
**Diagnosis and main criteria for inclusion:**	• Male and female Non-Fabry subjects aged 18 to 79 years<br>• Body mass index (BMI) within 18.0 to 35.0 kg/m^2<br>• Subjects with ESRD: estimated glomerular filtration rate (eGFR) according to the Modification of Diet in Renal Disease (MDRD) equation ($eGFR_{MDRD}$) of < 15 mL/min/1.73 m^2 at the screening visit<br>-OR-<br>Subjects with normal renal function: $eGFR_{MDRD}$ of ≥ 80 mL/min/1.73 m^2 at the screening visit

[2] Synopsis updated via Amendment 2 to clarify the enrollment of subjects on standard hemodialysis and hemodiafiltration

	Additionally, for subjects with ESRD:<br>• Subject receives hemodialysis (standard hemodialysis or hemodiafiltration) (at least 4 h every 72 h)<br>• Subject has been stable on their dialysis regimen for at least 2 months
**Study design:**	This study will be conducted as a single center, Phase 1, open-label, and non-randomized design in non-Fabry subjects with ESRD on hemodialysis (standard hemodialysis or hemodiafiltration) and matched control subjects with normal renal function.<br>The following treatments will be administered:<br>ESRD subjects on hemodialysis<br>• Period 1: Single oral dose of migalastat 24 h before start of dialysis (i.e., in a dialysis-free interval)<br>• Period 2: Single oral dose of migalastat immediately before start of dialysis<br>Periods 1 and 2, for which PK assessments will be done separately, will be carried out in a fixed sequence with a washout phase of at least 8 days in between.<br>Subjects with normal renal function<br>• Single oral dose of migalastat<br>Recruitment will start with the ESRD subjects. For safety reasons, one ESRD subject will be treated first and the safety data will be discussed between sponsor and investigator. If the safety data for this individual subject is considered acceptable, the other subjects of the group will be treated in parallel.<br>Subjects with ESRD and matched subjects with normal renal function will be recruited in parallel but not dosed at the same time. A matched subject with normal renal function will be enrolled after the follow-up visit of his/her matched ESRD subject.[3] These subjects will be matched 1:1 to subjects in the ESRD group based on their age (±10 years), body weight (±10 kg), and sex. In each group at least 2 subjects of each sex will be enrolled.
**Duration**	The overall duration of the study starts with the first subject signing informed consent (FSI) and ends with the last subject undergoing last visit (LSO).<br>The duration of individual subject participation is estimated to be:<br>• For subjects with normal renal function: approximately 4 weeks (up to 3 weeks screening period followed by 8 days for treatment phase and follow-up)<br>• For ESRD subjects: approximately 6 weeks (up to 3 weeks

[3] Changed via Amendment 2

	screening period followed by at least 16 days for treatment phase and follow-up)
**Methodology:**	Activities during the study are outlined in the schedule of event tables following this synopsis.<br><br>For each ESRD subject on hemodialysis, the study consists of:<br><br>• An ambulatory screening period before the first treatment period (Day -21 to Day -2), during which eligibility of the subjects will be assessed.<br>• Two periods (each from Day -1 to Day 4) with a single oral dose on Day 1 of each period. In each period, in the morning of Day -1, subjects will come to the study center for an ambulatory visit. Subjects will be hospitalized from the evening of Day -1 (~8 pm) until discharge on the morning of Day 3 (after all examinations and assessments are performed). During the hospitalization period, subjects will leave the study center for dialysis. On Day 4 subjects will return to the study center for an ambulatory visit.<br>• An ambulatory follow-up visit 7 days after the dose for Period 2.<br><br>For each subject with normal renal function, the study consists of:<br><br>• An ambulatory screening period (Day -21 to Day -2), during which eligibility of the subjects will be assessed.<br>• One period (from Day -1 to Day 3) with a single oral dose on Day 1. In the morning of Day -1, subjects will come to the study center for an ambulatory visit. Subjects will be hospitalized from the evening of Day -1 (~8 pm) until discharge in the morning of Day 3 (after all examinations and assessments are performed).<br>• An ambulatory follow-up visit 7 days after dosing.
**Type of control:**	Controlled (intra-individual comparison between subjects on hemodialysis and subjects with normal renal function)
**Number of subjects:**	12 subjects are planned (6 ESRD subjects [3 subjects on standard hemodialysis, 3 subjects on hemodiafiltration] and 6 subjects with normal renal function)
**Primary endpoints:**	Maximum observed concentration between time zero to 24h ($C_{max,0\text{-}24}$) and AUC of migalastat in plasma (area under the concentration-time curve from time zero to the last measurable concentration [$AUC_{0\text{-}t}$] and from time zero to 24h [$AUC_{0\text{-}24}$])
**Other PK endpoints:**	Plasma migalastat: migalastat concentrations and further PK parameters of migalastat in plasma (area under the concentration-time curve from time zero and extrapolated to infinity [$AUC_{0\text{-}\infty}$, subjects with normal renal function, Period 2 of ESRD subjects], time to

	maximum concentration [t_{max}], apparent terminal elimination half-life [$t_{1/2}$], apparent plasma clearance [CL/F], and apparent terminal phase volume of distribution [V_z/F])<br><br>Migalastat in dialysate: dialysis clearance (C_LD), volume of dialysate collected during the interval (VD), migalastat concentration in dialysate (CD), amount recovered in dialysate (A_eD), fraction of the dose recovered in dialysate (F_eD); and extraction coefficient or ratio of dialysis clearance and blood flow<br><br>Urine migalastat (when applicable, dependent on subjects’ ability to produce urine): migalastat concentrations and urine, PK parameters of migalastat (total amount excreted [A_e], fraction of the dose recovered in urine [F_e], and renal clearance [CLr])
**Time of measurement of primary endpoints:**	The PK blood samples will be collected as per the following:<br>• ESRD group: from pre-dose until 72 h after dosing in both periods<br>• Normal renal function group: from pre-dose until 48 h after dosing
**Secondary endpoints:**	Treatment-emergent adverse events (TEAEs), clinical safety laboratory test results, electrocardiogram (ECG) results, vital signs
**Plan for statistical analyses:**	All measured variables and derived PK parameters will be listed individually and, as appropriate, tabulated by descriptive statistics for ESRD subjects on standard hemodialysis, ESRD subjects on hemodiafiltration, subjects with normal renal function matched to ESRD subjects on standard hemodialysis, and subjects with normal renal function matched to ESRD subjects on hemodiafiltration. Figures will be provided as appropriate.<br><br>Descriptive statistics will provide the number of observations and the absolute and relative frequency of categorical variables. For the continuous variables, number of observations, arithmetic mean, standard deviation, coefficient of variation (if appropriate), median, minimum, and maximum will be given.<br><br>For the PK Analysis Set (all subjects for whom the primary PK parameters of migalastat can be derived from at least one treatment period will be included in the PK population):<br>• Descriptive statistics of PK parameters and concentrations will additionally include the geometric mean and the geometric standard deviation.<br><br>For the Safety Analysis Set (all enrolled subjects who received at least one dose of migalastat):<br>• Results of all safety measurements will be listed individually and, as appropriate, summarized.<br><br>Descriptive statistics will be calculated for laboratory parameters,

	vital signs (including body temperature), and ECG parameters. Physical examination findings will be listed.

2.1 Study flow chart

Table 2-1: Schedule of events – screening and period 1 – ESRD subjects on hemodialysis

Study phase	Screening	Period 1					
**Study Day**	**-21 to -2**	**Day -1 (morning)**	**Day -1 (~8 pm, check-in)**	**Day 1**	**Day 2**	**Day 3**	**Day 4**
Review informed consent	X						
Review inclusion / exclusion criteria	X		X[1]				
Medical history	X						
Physical examination	X			X[2]		X	
Concomitant medications	X	X	X	X	X	X	X
Admission to clinic			X				
Ambulatory visits	X	X					X
Adverse events	X	X	X	X	X	X	X
Vital signs (blood pressure, pulse rate)	X	X		X[3]	X	X	
Vital signs (respiratory rate, body temperature)	X						
Weight	X	X		X[2]			
Height	X						
12-Lead ECG	X	X		X[4]		X	
Blood sample collection for chemistry and hematology	X	X			X	X	
Urine sample collection for urinalysis (if able to produce urine)	X			X[2]		X	
Blood sample collection for virology	X						
Urine drug screening (if able to produce urine) and alcohol breath test	X		X				
eGFR calculation according to MDRD equation	X						

**Study phase**	**Screening**	**Period 1**					
**Study Day**	**-21 to -2**	**Day -1 (morning)**	**Day -1 (~8 pm, check-in)**	**Day 1**	**Day 2**	**Day 3**	**Day 4**
Pregnancy test in serum (females of childbearing potential only)	X	X					
**Migalastat administration**				**X**[5]			
**Dialysis**					**X**[5]		
PK blood sampling[6]				X	X	X	X
PK blood sampling from inlet and outlet line of dialysis machine[6]					X		
PK dialysate collection[6]					X		
PK urine sampling (if able to produce urine)[6]				X	X	X	X
Discharge from clinic						X[7]	

ECG = electrocardiogram, eGFR = estimated glomerular filtration rate, ESRD = end-stage renal disease, MDRD = Modification of Diet in Renal Disease, PK = pharmacokinetics

1: update/confirmation of eligibility criteria previously assessed at Screening
2: indicated assessments are to be performed pre-dose
3: blood pressure and pulse rate on Day 1 will be assessed at pre-dose, 1 h, 2 h, 4 h, 6 h, and 12 h post-dose
4: ECG on Day 1 will be assessed at pre-dose and 2 h post-dose
5: subjects will receive the study drug 24 h before start of dialysis
6: for sampling time points see Table 2-4
7: discharge is planned in the morning of Day 3, following completion of all assessments

Table 2-2: Schedule of events – period 2, including end of study – ESRD subjects on hemodialysis

**Study phase**	**Period 2***						**End of study/early termination**
**Study Day**	**Day -1 (morning)**	**Day -1 (~8 pm, check-in)**	**Day 1**	**Day 2**	**Day 3**	**Day 4**	**7 days after last dosing**
Physical examination			X[1]		X		X
Concomitant medications	X	X	X	X	X	X	X
Admission to clinic		X					
Ambulatory visits	X					X	X
Adverse events	X	X	X	X	X	X	X
Vital signs (blood pressure, pulse rate)	X		X[2]	X	X		X
Vital signs (respiratory rate, body temperature)							X
Weight	X		X[1]				X
12-Lead ECG	X		X[3]		X		X
Blood sample collection for chemistry and hematology	X			X	X		X
Urine sample collection for urinalysis (if able to produce urine)			X[1]		X		X
Urine drug screening (if able to produce urine) and alcohol breath test		X					
Pregnancy test in serum (females of childbearing potential only)	X						X
**Migalastat administration**			**X[4]**				
**Dialysis**			**X[4]**				

Study phase	Period 2*						End of study/early termination
Study Day	Day -1 (morning)	Day -1 (~8 pm, check-in)	Day 1	Day 2	Day 3	Day 4	7 days after last dosing
PK blood sampling[5]			X	X	X	X	
PK blood sampling from inlet and outlet line of dialysis machine[5]			X				
PK dialysate collection[5]			X				
PK urine sampling (if able to produce urine) [5]			X	X	X	X	
Discharge from clinic					X[6]		

ECG = electrocardiogram, ESRD = end-stage renal disease, PK = pharmacokinetics

* There will be a washout phase of at least 8 days between dosing in Period 1 and 2
1: indicated assessments are to be performed pre-dose
2: blood pressure and pulse rate on Day 1 will be assessed at pre-dose, 1 h, 2 h, 4 h, 6 h, and 12 h post-dose
3: ECG on Day 1 will be assessed at pre-dose and 2 h post-dose
4: subjects will receive the study drug immediately before start of dialysis
5: for sampling time points see Table 2-5
6: discharge is planned in the morning of Day 3, following completion of all assessments

Table 2-3: Schedule of events – screening to end of study – subjects with normal renal function

**Study Day**	**-21 to -2**	**Day -1 (morning)**	**Day -1 (~8 pm, check-in)**	**Day 1**	**Day 2**	**Day 3**	**End of study/early termination**<br>**7 days after dosing**
Review informed consent	X						
Review inclusion / exclusion criteria	X		X[1]				
Medical history	X						
Physical examination	X			X[2]		X	X
Concomitant medications	X	X	X	X	X	X	X
Admission to clinic			X				
Ambulatory visits	X	X					X
Adverse events	X	X	X	X	X	X	X
Vital signs (blood pressure, pulse rate)	X	X		X[3]	X	X	X
Vital signs (respiratory rate, body temperature)	X						X
Weight	X	X		X[2]			X
Height	X						
12-Lead ECG	X	X		X[4]		X	X
Blood sample collection for chemistry and hematology	X	X			X	X	X
Urine sample collection for urinalysis	X			X[2]		X	X
Blood sample collection for virology	X						
Urine drug screening and alcohol breath test	X		X				
eGFR calculation according to MDRD equation	X						

**Study Day**	**-21 to -2**	**Day -1 (morning)**	**Day -1 (~8 pm, check-in)**	**Day 1**	**Day 2**	**Day 3**	**End of study/early termination**<br>**7 days after dosing**
Pregnancy test in serum (females of childbearing potential only)	X	X					X
Migalastat administration				**X**			
PK blood sampling[5]				X	X	X	
PK urine sampling[5]				X	X	X	
Discharge from clinic						X[6]	

ECG = electrocardiogram, eGFR = estimated glomerular filtration rate, MDRD = Modification of Diet in Renal Disease, PK = pharmacokinetics

1: update/confirmation of eligibility criteria previously assessed at Screening
2: indicated assessments are to be performed pre-dose
3: blood pressure and pulse rate on Day 1 will be assessed at pre-dose, 1 h, 2 h, 4 h, 6 h, and 12 h post-dose
4: ECG on Day 1 will be assessed at pre-dose and 2 h post-dose
5: for sampling time points see Table 2-6
6: discharge is planned in the morning of Day 3, following completion of all assessments

2.2 Sampling time points for pharmacokinetics

Table 2-4: ESRD subjects on hemodialysis - PK sampling time points (period 1)

Period 1 Study Day	1										2							3	4
**hh:mm post dose**	**Pre-dose**	**00:00**	**01:00**	**02:00**	**03:00**	**04:00**	**06:00**	**08:00**	**10:00**	**12:00**	**24:00**	**24:05**	**25:00**	**26:00**	**27:00**	**28:00**	**28:05**	**48:00[1]**	**72:00**
Migalastat administration		x																	
Blood draw[2]	x		x	x	x	x	x	x	x	x	x	x[3]	x	x	x	x	x	x	x
Blood (inlet line of dialysis machine)[2]												x	x	x	x	x	x		
Blood (outlet line of dialysis machine)[2]												x	x	x	x	x	x		
Urine[4]	x	→	→	→	→	→	→	→	→	→	→|→	→	→	→	→	→	→	→|→	→
Dialysis												→	→	→	→	→	→		
Dialysate sampling[5]												x	x	x	x	x	x[6]		

ESRD = end-stage renal disease, PK = pharmacokinetics

→: sampling to be done continuously, starting from the time point indicated; →|→: end of previous continuous sampling period/start of new continuous sampling period

1: discharge

2: blood draws are to be taken within ± 10 minutes of schedule time until discharge from the study ward and within ± 120 minutes after discharge; the exceptions are the draws at 00:05 past the hour, which must be taken by 00:15 past the hour

3: blood draw to occur immediately before start of dialysis

4: total urine volume MUST be recorded for each collection interval, even if 0 (collection intervals: pre-dose / 0 to 24h, 24 to 48h, 48 to 72h). After discharge on Day 3 subjects will collect the urine at home. The subjects will be instructed to store the urine container in the fridge.

5: the volume of waste dialysate will be recorded at the end of dialysis treatment.

6: just after end of dialysis

Table 2-5: ESRD subjects on hemodialysis - PK sampling time points (period 2)

**Period 2 Study Day**	**1**												**2**					**3**	**4**
**hh:mm post dose**	**Pre-dose**	**00:00**	**00:05**	**01:00**	**02:00**	**03:00**	**04:00**	**04:05**	**06:00**	**08:00**	**10:00**	**12:00**	**24:00**	**25:00**	**26:00**	**27:00**	**28:00**	**48:00[1]**	**72:00**
Migalastat administration		x																	
Blood draw[2]	x		x[3]	x	x	x	x	x	x	x	x	x	x	x	x	x	x	x	x
Blood (inlet line of dialysis machine)[2]			x	x	x	x	x	x											
Blood (outlet line of dialysis machine)[2]			x	x	x	x	x	x											
Urine[4]	x	→	→	→	→	→	→	→	→	→	→	→	→|→	→	→	→	→	→|→	→
Dialysis			→	→	→	→	→	→											
Dialysate sampling[5]			x	x	x	x	x	x[6]											

ESRD = end-stage renal disease, PK = pharmacokinetics

→: sampling to be done continuously, starting from the time point indicated; →|→: end of previous continuous sampling period/start of new continuous sampling period

1: discharge

2: blood draws are to be taken within ± 10 minutes of schedule time until discharge from the study ward and within ± 120 minutes after discharge, the exceptions are the draws at 00:05 past the hour, which must be taken by 00:15 past the hour

3: immediately before start of dialysis

4: total urine volume MUST be recorded for each collection interval, even if 0 (collection intervals: pre-dose / 0 to 24h, 24 to 48h, 48 to 72h). After discharge on Day 3 subjects will collect the urine at home. The subjects will be instructed to store the urine container in the fridge.

5: the volume of waste dialysate will be recorded at the end of dialysis treatment

6: just after end of dialysis

Table 2-6: Subjects with normal renal function - PK sampling time points

**Study Day**	**1**											**2**	**3**
**hh:mm post dose**	**Predose**	**00:00**	**00:05**	**01:00**	**02:00**	**03:00**	**04:00**	**06:00**	**08:00**	**10:00**	**12:00**	**24:00**	**48:00[1]**
Migalastat administration		x											
Blood draw[2]	x		x	x	x	x	x	x	x	x	x	x	x
Urine[3]	x	→	→	→	→	→	→	→	→	→	→	→|→	→

PK = pharmacokinetics

→: sampling to be done continuously, starting from the time point indicated; →|→: end of previous continuous sampling period/start of new continuous sampling period
1: discharge
2: blood draws are to be taken within ± 10 minutes of schedule time, with the exception of the draw at 00:05, which must be taken by 00:15
3: total urine volume MUST be recorded for each collection interval, even if 0 (collection intervals: pre-dose / 0 to 24h and 24 to 48h)

3 Table of contents

1 PROTOCOL APPROVAL **2**
Signature sponsor 2
Signature investigator 3

2 SYNOPSIS - AMENDED **4**
2.1 Study flow chart 9
2.2 Sampling time points for pharmacokinetics 15

3 TABLE OF CONTENTS **18**

4 LIST OF ABBREVIATIONS AND DEFINITIONS OF TERMS **21**

5 ETHICS AND LEGAL ASPECTS **24**
5.1 Independent ethics committee and regulatory authority 24
5.2 Ethical and legal conduct of the study 24
5.3 Subject information and consent 24

6 INVESTIGATORS AND STUDY ADMINISTRATIVE STRUCTURE - AMENDED25

7 INTRODUCTION **26**
7.1 Background 26
7.2 Description of the investigational medicinal product 27
7.3 Rationale of the study 28
7.4 Risk/benefit analysis 29
7.5 Relevant guidelines 31

8 STUDY OBJECTIVES AND ENDPOINTS - AMENDED **32**

9 INVESTIGATIONAL PLAN **33**
9.1 Overall study design and plan description - amended 33
9.2 Discussion of study design, including the choice of control groups - amended 35
9.3 Selection of study population - amended 36
9.3.1 Inclusion criteria 36
9.3.2 Exclusion criteria 38
9.3.3 Discontinuation of subjects from treatment or assessment 43
9.3.4 Subject replacement - amended 44
9.3.5 Premature discontinuation of the study 44
9.3.6 Subject identification 44
9.4 Treatments 44
9.4.1 Treatments administered 44
9.4.2 Identity of investigational medicinal products 45
9.4.3 Method of assigning subjects to treatment groups 46

9.4.4 Selection of doses on the study 46
9.4.5 Selection and timing of dose for each subject 46
9.4.6 Blinding 47
9.4.7 Prior and concomitant therapy 47
9.4.8 Treatment compliance 47
9.4.9 Treatment after end of study participation 48
9.4.10 Restrictions and precautions 48
9.5 Population characteristics, pharmacokinetic and safety endpoints 48
9.5.1 Populations characteristics 48
9.5.2 Pharmacokinetics 49
9.5.3 Safety pharmacology 51
9.6 Appropriateness of measurements 53
9.7 Statistical methods and determination of sample size 53
9.7.1 Statistical and analytical plans 53
9.7.2 Determination of sample size - amended 58

10 DATA HANDLING AND RECORD KEEPING 58
10.1 Case report forms and recording of data 58
10.1.1 Electronic records 58
10.2 Data collection (eCRF) 59
10.3 Data quality assurance 59
10.3.1 Archiving of data and data retention 59
10.3.2 Data access 60

11 QUALITY ASSURANCE AND QUALITY CONTROL, AUDITS, AND INSPECTIONS 60
11.1 Quality control and quality assurance 60
11.2 Audit 61
11.3 Inspection 61

12 CONFIDENTIALITY OF SUBJECT DATA 61

13 REPORTING 62

14 PUBLICATION POLICY 62

15 INSURANCE 62

16 RESPONSIBILITIES AND FINANCES 62

17 REFERENCES 63

18 AMENDMENTS 64

18.1 Amendment 1 64
18.1.1 Overview of changes to the study 64
18.1.2 Changes to the protocol text 64
18.2 Amendment 2 66
18.2.1 Overview of changes to the study 66
18.2.2 Changes to the protocol text 67

19 ADVERSE EVENTS AND SERIOUS ADVERSE EVENTS 76
19.1 Definitions 76
19.1.1 Adverse event 76
19.1.2 Serious adverse events 77
19.2 Relationship to study drug 79
19.3 Severity assessment 80
19.4 Reporting events 80
19.4.1 Reporting adverse events 80
19.4.2 Reporting Serious Adverse Events - amended 80
19.4.3 Additional Reporting Requirements for Suspected Unexpected Serious Adverse Reactions 81
19.5 Other reporting situations 82
19.5.1 Pregnancy 82
19.5.2 Medication errors, including overdose 82
19.5.3 Reporting of possible study drug product quality defects 82

Table of Tables

Table 2-1: Schedule of events – screening and period 1 – ESRD subjects on hemodialysis 9
Table 2-2: Schedule of events – period 2, including end of study – ESRD subjects on hemodialysis 11
Table 2-3: Schedule of events – screening to end of study – subjects with normal renal function 13
Table 2-4: ESRD subjects on hemodialysis - PK sampling time points (period 1) 15
Table 2-5: ESRD subjects on hemodialysis - PK sampling time points (period 2) 16
Table 2-6: Subjects with normal renal function - PK sampling time points 17
Table 9-1: Overview of renal function groups - amended 33
Table 9-2: Treatments administered 44
Table 9-3: Identity of test drug 45
Table 9-4: Study restrictions 48

4 List of abbreviations and definitions of terms

AE	adverse event
A_e	total amount excreted
A_eD	amount recovered in dialysate
ALT	alanine aminotransferase
AMG	*Arzneimittelgesetz* (German Drug Law)
Anti-HCV	anti-hepatitis C-virus antibodies
AP	alkaline phosphatase
AST	aspartate aminotransferase
AUC	area under the concentration-time curve
AUC_{0-24}	area under the concentration-time curve from time zero to 24h
$AUC_{0-\infty}$	area under the concentration-time curve from time zero and extrapolated to infinity
AUC_{0-t}	area under the concentration-time curve from time zero to the last measurable concentration
BMI	body mass index
CD	migalastat concentration in dialysate
CHMP	Committee for Medicinal Products for Human use
CK	creatine phosphokinase
C_L/D	dialysis clearance
CL/F	apparent plasma clearance
CL_r	renal clearance
$C_{max,0-24}$	maximum observed concentration between time zero to 24h
CRF	case report form
CRS-Kiel	CRS Clinical Research Services Kiel GmbH
CRS-Mannheim	CRS Clinical Research Services Mannheim GmbH
CYP	cytochrome P450
DAkkS	Deutsche Akkreditierungstelle GmbH
DBP	diastolic blood pressure

e.g.	*exempli gratia* (for example)
ECG	electrocardiogram
eCRF	electronic case report form
eGFR	estimated glomerular filtration rate
EMA	European Medicines Agency
ESRD	end-stage renal disease
EU	European Union
F_e	fraction of the dose recovered in urine
F_eD	fraction of the dose recovered in dialysate
FSH	follicle stimulating hormone
FSI	first subject signing informed consent
gamma-GT	gamma glutamyl transpeptidase
GCP	Good Clinical Practice
GL-3	globotriaosylceramide
GLA	gene encoding α-galactosidase A
GMP	Good Manufacturing Practice
HBsAg	hepatitis B-virus surface antigen
HCl	hydrochloride
HCV	Hepatitis C virus
HDL	high-density lipoprotein
HIV	human immunodeficiency virus
i.e.	*id est* (that is)
IB	Investigator’s Brochure
ICH	International Conference on Harmonization
IEC	Independent Ethics Committee
IMP	investigational medicinal product
INN	international non-propriety names
ISF	Investigator Site File
IV	intravenous

LC/MS-MS	Liquid Chromatography Tandem Mass Spectrometry
LDH	lactate dehydrogenase
LDL	low-density lipoprotein
LLOQ	lower limit of quantification
LSO	last subject undergoing last visit
lyso-Gb3	globotriaosylsphingosine
MCH	mean corpuscular hemoglobin
MCHC	mean corpuscular hemoglobin concentration
MCV	mean corpuscular volume
MDRD	Modification of Diet in Renal Disease
MedDRA	Medical Dictionary for Regulatory Activities
PC	pharmacological chaperone
P-gp	P-glycoprotein
pH	*Pondus hydrogenii* (log of the ion concentration)
PHI	Personal Health Information
PID	subject identification number
PK	pharmacokinetics
pm	post meridiem (hours between 12:00 o'clock and 24:00 o'clock)
QOD	every-other-day
SAE	serious adverse event
SAP	statistical analysis plan
SAS	Statistical Analysis System
SBP	systolic blood pressure
SmPC	Summary of Product Characteristics
SOP	Standard Operating Procedures
SUSAR	suspected unexpected serious adverse reaction
$t_{1/2}$	apparent terminal elimination half-life
TEAE	treatment-emergent adverse event

t_{max}	time to maximum concentration
US	Unites States
VD	volume of dialysate collected during the interval
V_{ss}	steady-state volume of distribution
V_z/F	apparent terminal phase volume of distribution
α-Gal A	α-galactosidase

5 Ethics and legal aspects

5.1 Independent ethics committee and regulatory authority

The clinical study protocol and any substantial amendments will be presented to an Independent Ethics Committee (IEC) and the responsible regulatory authority for review.

Information provided to the subjects and recruitment advertisements (if applicable) will also be reviewed by the IEC.

The positive vote of the IEC and the approval of the regulatory authority will be obtained prior to the start of the study.

A list of IECs consulted will be provided in the clinical study report / Trial Master File.

5.2 Ethical and legal conduct of the study

The study will be performed in accordance with the study protocol, the ethical principles that have their origin in the current accepted version of the Declaration of Helsinki and in accordance with ICH GCP and applicable local laws and regulations.

5.3 Subject information and consent

Written informed consent must be obtained from subjects prior to participation in the study and prior to completion of any study-related procedure.

The subjects will voluntarily confirm their willingness to participate in the study, after having been informed by a physician in writing and verbally of all aspects of the study that are relevant to the subject's decision to participate. They will be informed about legal requirements concerning data protection and have to agree to the direct access, recording and processing of their personal data which includes special categories of data such as health data. Moreover, they will be informed that withdrawal of their informed consent does not impact data collected up to the point of withdrawal, which will be retained and analyzed. The subject can at any time object in full to the processing of their data outside the protocol for research purposes only.

The informed consent form for study participation including the data protection declaration of consent has to be signed and personally dated by the subject and by the investigator prior to any study -specific procedures. A copy of the signed informed consent form will be given to the subject.

Before informed consent is obtained, the investigator has to provide the subject sufficient time and opportunity to inquire about details of the study and to decide whether or not to participate in the study. All questions about the study have to be answered to the satisfaction of the subject.

The subjects will be informed by a physician in a timely manner if new information becomes available that may be relevant to their willingness to continue participation in the study. The communication of this information will be documented. The subjects will receive a copy of any amendments to the written information and a copy of the signed and dated consent form updates.

Subjects will be informed that they are free to withdraw from the study at any time at their own discretion without having to provide reasons for this decision.

6 Investigators and study administrative structure - amended

Sponsor
Amicus Therapeutics, Inc.
1 Cedar Brook Drive
Cranbury, NJ 08512
Phone: +1 609-662-2000
Fax: +1 609-662-2805

Legal representative in the European Union
Amicus Therapeutics Europe Limited
Block 1[4]
Corporate Park
Ballycoolen Road
Blanchardstown
Dublin
D15 AKK1 Ireland

[4] Address of legal representative in the European Union changed via Amendment 1

Sponsor's medical expert

[redacted]

Investigator

[redacted]

Deputy investigators

[redacted]

A list of study personnel, laboratories and relevant contacts for the study will be maintained by the Sponsor and filed in the Trial Master File.

A list of study personnel at the study site and a list of all relevant contacts for the center will be maintained by the study center and filed in the Investigator Site File.

Sponsor and investigator will sign the protocol, protocol amendments, and integrating protocols.

The investigator will sign the protocol, protocol amendments, and integrating protocols before they come into effect at the site.

7 Introduction

7.1 Background

<u>Fabry disease</u>

Fabry disease is a progressive X-linked lysosomal storage disorder with an estimated prevalence of 1:117,000 up to 1:40,000 which affects males and females. Fabry disease-causing gene encoding α-galactosidase A (*GLA)* variants result in a

deficiency of the lysosomal enzyme α-galactosidase A (α-Gal A) that is required for glycosphingolipid substrate (e.g., globotriaosylceramide [GL-3], lyso-Gb3) metabolism. Reduced α-Gal A activity is, therefore, associated with the progressive accumulation of substrate in vulnerable organs and tissues, which leads to the morbidity and mortality associated with Fabry disease; however, initiation of therapy in the early stages of the disease with life-long treatment may provide an opportunity to slow disease progression.

The clinical manifestations of Fabry disease span a broad spectrum of severity and seem to be associated with residual α-galactosidase (α-Gal A) activity levels that vary widely according to the specific Fabry disease-causing genotype and the specific genetic background of a given patient.

Individuals with classic Fabry disease have little or no detectable α-Gal A activity levels and are the most severely affected. If these patients are not treated, their life expectancy is reduced and death usually occurs in the third to fifth decade of life from renal failure, cardiac dysfunction, or stroke.

Enzyme replacement therapy and Galafold® are commercially available treatments for Fabry disease. Details on Galafold® are provided in Section 7.2.

7.2 Description of the investigational medicinal product

Migalastat hydrochloride (HCl) (Galafold®) is a potent, competitive, and reversible inhibitor of α-Gal A.

Galafold® is currently licensed in Europe, Switzerland, Israel, Australia, Canada, Japan, South Korea, and the United States for the treatment of adults with a confirmed diagnosis of Fabry disease. Additional Marketing Applications are currently under review.

Preclinical *in vitro* and *in vivo* pharmacology studies have demonstrated that migalastat is a pharmacological chaperone for α-Gal A that selectively and reversibly binds to the active site of both Wild Type and specific mutant forms of α-Gal A.

The recommended dosage regimen is 150 mg migalastat HCl (equivalent to 123 mg migalastat free base) once every other day at the same time of day.

Safety and tolerability

The most common adverse reaction in clinical studies was headache, which was experienced by approximately 10% of patients who received migalastat.

Very common adverse reactions (≥ 1/10) were: abdominal pain, diarrhoea, nausea, fatigue, pain, dizziness and headache.
Common adverse reactions (≥ 1/100, < 1/10) were: rash and pruritus.[5]

[5] Updated in consistency with update of Investigator's Brochure (Version 17)

In clinical studies, to date, 3 serious adverse events (SAEs) considered possibly related to migalastat have been reported: fatigue and paresthesia in one subject and proteinuria in one subject.

<u>Pharmacokinetics</u>

The absolute bioavailability (AUC) for a single oral 150 mg migalastat HCl dose was approximately 75%. Following a single oral dose of 150 mg migalastat HCl solution, the time to peak plasma concentration was approximately 3 h. Plasma migalastat exposure ($AUC_{0-\infty}$) and C_{max} demonstrated dose-proportional increases at migalastat HCl oral doses from 50 mg to 1250 mg.

In healthy volunteers, the volume of distribution (V_z/F) of migalastat following ascending single oral doses (25-675 mg migalastat HCl) ranged from 77 to 133 L, indicating it is well distributed into tissues and greater than total body water (42 L). There was no detectable plasma protein binding following administration of [^{14}C]-migalastat in the concentration range between 1 and 100 µM.

Three minor (hydrolyzed O-glucuronide) metabolites have been elucidated with no one metabolite comprising more than 6% of the dose. Migalastat has neither been shown to induce or inhibit cytochrome P450 (CYP) isoenzymes, nor is it a substrate for P-glycoprotein (P-gp).

A pharmacokinetic (PK) study in healthy volunteers with 150 mg [^{14}C]-migalastat HCl revealed that approximately 77% and 20% of the radiolabeled dose were recovered in urine and feces, respectively.

Migalastat is rapidly cleared from plasma (apparent total plasma clearance of drug after extravascular administration [CL/F] approximately 4 to 6 L/h) with a mean half-life of approximately 4 h.

Further details can be found in the latest available version of the Investigator's Brochure (IB) of AT1001 (migalastat HCl), which contains comprehensive information on the study drug[1] and in the Summary of Product Characteristics (SmPC) of Galafold® [2].

7.3 Rationale of the study

Galafold® currently is not recommended for use in patients with Fabry disease who have estimated glomerular filtration rate (eGFR) less than 30 mL/min/1.73 m^2.

Migalastat is primarily excreted unchanged in urine. Following a single oral dose of 150 mg migalastat HCl in subjects with mild (GFR ≥60 and <90 mL/min/1.73 m^2), moderate (GFR ≥30 and <60 mL/min/1.73 m^2), or severe (GFR ≥15 and <30 mL/min/1.73 m^2) renal impairment, mean plasma migalastat $AUC_{0-\infty}$ increased with degree of renal impairment (up to 4.3-fold). C_{max} was unchanged across groups. Plasma migalastat $t_{1/2}$ increased significantly and plasma clearance decreased significantly with worsening renal function (AT1001-015). Insufficient data are available in subjects with substantial renal impairment to make recommendations

regarding dosing adjustments in these subjects. Migalastat has not been studied in dialysis subjects.

The rationale of this study is to verify the evaluation of PK and physical characteristics of migalastat that suggests recovery in dialysate is feasible following hemodialysis, and to provide PK and safety data to allow expansion of the current Galafold® label to include end-stage renal disease (ESRD) Fabry patients with amenable *GLA* variants. Depending on characterization of PK and recovery in dialysate following hemodialysis, dose adjustment may be necessary for Fabry patients with ESRD.

<u>Prediction of dialyzability for migalastat</u>

The primary factors determining the ability of a drug to be removed by dialysis are molecular weight, protein binding, volume of distribution and red blood cell partitioning. Water solubility and ionization state at physiological pH can play a role but to a lesser extent. Migalastat is a relatively small molecule with a molecular weight of only 163.17 g/mol, well below the molecular weight (>1000 g/mol) considered to limit dialysis of small molecules[3]. Migalastat was found to have very low to negligible binding to human plasma proteins. High plasma protein binding is associated with limiting dialyzability. High volume of distribution (>2 L/kg or ~140 L for a 70 kg subject) has been associated with poor ESRD dialyzability. In the clinical study AT1001-018, steady-state volume of distribution (V_{ss}) for migalastat following intravenous (IV) administration ranged from 17.9 to 30.2 L (0.2 to 0.6 L/kg)[4][5]. Migalastat does not exhibit extensive partitioning into red blood cells, a property associated with poor dialyzability[6]. In summary, migalastat does not exhibit any of the drug properties generally associated with poor dialyzability and, therefore, is predicted to be dialyzable.

7.4 Risk/benefit analysis

<u>Risks resulting from the IMP</u>

Adverse reactions observed in clinical studies are provided in Section 7.2.

Phase 2 clinical studies established that a dose of 150 mg administered every-other-day (QOD) provides a C_{max} of approximately 10 µM (1631 ng/mL), the mean concentration necessary for binding and trafficking of most mutant forms of α-Gal A from the endoplasmic reticulum to the lysosome. The 150 mg QOD dose was administered in Phase 3 clinical studies and is the approved dose regimen in the US, EU, and Japan for treating Fabry patients with amenable mutations.

Migalastat, a highly water soluble drug, is eliminated primarily by the kidneys. Approximately 75% of the radiolabeled dose was recovered in urine in a mass-balance study. As such, the current label restricts migalastat administration to patients with eGFR no lower than 30 mL/min/1.73m^2. After a single dose of 150 mg migalastat HCl, compared to the normal renal function group, the plasma migalastat $AUC_{0-\infty}$ increased 1.2-, 1.8-, and 4.3-fold in mild, moderate, and severe renal

function groups, respectively. The adverse event (AE) profile was similar across categories of renal impairment. The severe renal impairment group had a similar C_{max} to normal renal function, but significantly increased terminal half-life and decreased plasma clearance (Study AT1001-015). Dose adjustments in severe renal impairment based on modeling and simulations for subjects with eGFR <30 to ≥15 mL/min/1.73 m^2 suggest that the 150 mg dose administered over 4 days in subjects with eGFR from < 30 to ≥ 20 mL/min/1.73 m^2, and over 7 days for subjects with eGFR <20 to ≥15 mL/min/1.73 m^2, would provide sufficient time to clear migalastat plasma levels to trough concentrations similar to normal renal function. For dialysis subjects, the 150 mg dose will be tested in this clinical study to determine if proper concentrations are provided for intracellular trafficking followed by removal of the majority of the dose by dialysis.

In case of overdose, general medical care is recommended. Headache and dizziness were the most common treatment-related AEs reported at doses of migalastat HCl of up to 1250 mg and 2000 mg, respectively.

There are limited data from the use of migalastat in pregnant women. Therefore, women of childbearing potential enrolled in this study have to use appropriate, reliable contraception for the study duration and for at least 30 days following (last) dosing.

A female subject is considered to be of reproductive potential (i.e., ovulating, pre-menopausal, not surgically sterile) if she has functional ovaries, ducts, and uterus with no impairment that would cause sterility.

This includes women with oligomenorrhea (even severe), and women who are pre-menopausal or who have just begun to menstruate. A female is considered to be of non-reproductive potential if she is at least 26 weeks post documented surgical sterilization (includes hysterectomy, oophorectomy, bilateral tubal ligation, or salpingectomy) or is postmenopausal and ≥ 2 years without menses. Female subjects who are post-menopausal < 2 years must be confirmed menopausal at Screening by Follicle Stimulating Hormone (FSH) and estradiol levels, based on the central laboratory’s normal ranges for women in menopause.

Transient and fully reversible infertility in male rats was associated with migalastat treatment at all doses assessed. Male subjects should maintain reliable contraceptive methods while taking migalastat.

<u>Risks resulting from the planned interventions</u>

The frequent blood sampling (by single vein puncture and / or indwelling cannula) may be accompanied by mild pain, hematoma and in rare cases inflammation of the vessel wall or injury of a nerve. Awareness should be raised to the possibility of a vasovagal attack or syncope (marked by pallor, nausea, sweating, bradycardia, and decrease in arterial blood pressure which, when below critical level, results in dizziness and /or loss of consciousness) during the sampling procedure.

The use of adhesive electrodes (electrocardiogram [ECG] leads) and / or adhesive dressings may be accompanied by mild and transient reddening and / or itching of the skin.

The total amount of blood withdrawn during the study will be approx. 108 mL in healthy subjects within about 4 weeks and approx. 331 mL in subjects on hemodialysis within about 6 weeks.

The study will be performed under controlled in-house conditions. During the clinical study, safety will be closely monitored with special attention to cardiovascular parameters (blood pressure, pulse rate, electrocardiogram), safety laboratory and well-being. An end of study examination will be performed before the subjects will be discharged from the study.

This study will be performed in non-Fabry ESRD subjects on hemodialysis as well as in age-, body weight-, and gender-matched healthy subjects. The subjects do not benefit from treatment. However, the data to be obtained from this study in regard to safety, tolerability and PK will form the basis for treatment of ESRD patients with Fabry disease who are on hemodialysis with Galafold®. Inclusion and exclusion criteria are chosen to enable a uniform study population and to minimize possible risks due to administration of migalastat.

The importance of the objective of this study is considered to outweigh the risks and burdens to the subjects. Measures are implemented to minimize burdens and risks for subjects. The benefit/risk assessment according to the German Drug Law (AMG, § 40, Abs 1, Nr. 2) is favorable and justifies the planned study in healthy volunteers.

7.5 Relevant guidelines

This clinical study protocol is developed according to ICH GCP and applicable local regulations and guidelines.

Furthermore, the recommendations of the following guidelines are considered for this study:

- Draft Guidance for Industry: Pharmacokinetics in patients with impaired renal function: study design, data analysis, and impact on dosing and labeling. Food and Drug Administration. March 2010.
- Guideline on the evaluation of the pharmacokinetics of medicinal products in patients with decreased renal function. EMA/CHMP/83874/2014. 17 Dec 2015.

8 Study objectives and endpoints - amended

Objectives[6]	Endpoints	Comments
Primary objective<br><br>To characterize the pharmacokinetics (PK) of migalastat in non-Fabry end-stage renal disease (ESRD) subjects who are receiving hemodialysis treatment (standard hemodialysis or hemodiafiltration)	Primary endpoints<br><br>*Main endpoints*<br><br>$C_{max,0-24}$ and AUC (AUC_{0-t} and AUC_{0-24}) of migalastat in plasma<br><br>*Other endpoints*<br><br>*Plasma migalastat*:<br>migalastat concentrations and further PK parameters of migalastat in plasma:<br>$AUC_{0-\infty}$ (subjects with normal renal function, Period 2 of ESRD subjects), t_{max}, $t_{1/2}$, CL/F, and V_z/F<br><br>*Migalastat in dialysate*<br>C_LD, VD, CD, A_eD, F_eD, and extraction coefficient or ratio of dialysis clearance and blood flow<br><br>*Urine migalastat (when applicable, dependent on subjects' ability to produce urine):*<br>migalastat concentrations and urine PK parameters of migalastat (A_e, Fe, CL_r)	See Section 9.5.2.3
Secondary objective<br><br>To assess the safety and tolerability of migalastat in non-Fabry ESRD subjects who are receiving hemodialysis treatment (standard hemodialysis or hemodiafiltration)	Secondary endpoints<br><br>Treatment-emergent adverse events<br><br>Clinical safety laboratory test results, ECG results, vital signs	See Section 9.5.3

[6] Objectives updated via Amendment 2 to clarify the enrollment of subjects on standard hemodialysis and hemodiafiltration

9 Investigational plan

9.1 Overall study design and plan description - amended[7]

This study will be conducted as a single center, Phase 1, open-label, and non-randomized design in 6 non-Fabry subjects with ESRD on hemodialysis (3 subjects on standard hemodialysis and 3 subjects on hemodiafiltration) and in 6 matched control subjects with normal renal function. (Please note: In instances within the protocol where standard hemodialysis and hemodiafiltration are not specified, the term "hemodialysis" refers to both.).

Renal function of the subjects will be classified by the estimated glomerular filtration rate (eGFR) according to Modification of Diet in Renal Disease (MDRD) equation (see Section 9.5.1.3).

Details are provided in Table 9-1.

Table 9-1: Overview of renal function groups - amended

**Renal function**	**eGFR according to MDRD equation [mL/min/1.73 m²]**	**Number of subjects**
ESRD on hemodialysis (standard hemodialysis or hemodiafiltration)	<15	6 (3 on standard hemodialysis, 3 on hemodiafiltration)
Normal renal function (matched 1:1 to subjects in the ESRD group based on age [±10 years], body weight [±10 kg], and sex; in each group at least 2 subjects of each sex will be enrolled).	≥80	6

eGFR: estimated glomerular filtration rate, ESRD: end-stage renal disease, MDRD: Modification of Diet in Renal Disease

Subjects with normal renal function will be matched 1:1 to subjects in the ESRD group based on their age (±10 years), body weight (±10 kg), and sex. In each group at least 2 subjects of each sex will be enrolled.

The following treatments will be administered:

ESRD subjects on hemodialysis

- Period 1: Single oral dose of 150 mg migalastat HCl 24 h before start of dialysis (i.e. in a dialysis-free interval)
- Period 2: Single oral dose of 150 mg migalastat HCl immediately before start of dialysis

[7] Section updated via Amendment 2 to clarify the enrollment of subjects on standard hemodialysis and hemodiafiltration

Periods 1 and 2, for which PK assessments will be done separately, will be carried out a fixed sequence with a washout phase of at least 8 days in between.

Subjects with normal renal function

- Single oral dose of 150 mg migalastat HCl

Order of recruitment

Recruitment will start with the ESRD subjects. For safety reasons, one ESRD subject will be treated first and the safety data will be discussed between sponsor and investigator. If the safety data for this individual subject is considered acceptable, the other subjects of the group will be treated in parallel.

Subjects with ESRD and matched subjects with normal renal function will be recruited in parallel but not dosed at the same time. A matched subject with normal renal function will be enrolled after the follow-up visit of his/her matched ESRD subject.

For each ESRD subject on hemodialysis, the study consists of:

- An ambulant screening period before the first treatment period (Day -21 to Day -2), during which eligibility of the subjects will be assessed.
- Two periods (each from Day -1 to Day 4) with single oral doses on Day 1 of each period. In each period, in the morning of Day-1, subjects will come to the study center for an ambulatory visit. Subjects will be hospitalized from the evening of Day -1 (~8 post meridiem [pm]) until discharge in the morning of Day 3 (after all examinations and assessments are performed). During the hospitalization period, subjects will leave the study center for dialysis. On Day 4 subjects will return to the study center for an ambulatory visit.
- An ambulant follow-up visit 7 days after last dosing for Period 2.

For each subject with normal renal function, the study consists of:

- An ambulant screening period (Day -21 to Day -2), during which eligibility of the subjects will be assessed.
- One period from Day -1 to Day 3 with a single oral dose on Day 1. In the morning of Day -1, subjects will come to the study center for an ambulatory visit. Subjects will be hospitalized from the evening of Day -1 (~8 pm) until discharge in the morning of Day 3 (after all examinations and assessments are performed).
- An ambulant follow-up visit 7 days after dosing.

Activities during the study are outlined in the flow charts in Section 2.1. Sampling time points of blood, urine, and dialysate for PK are provided in Section 2.2.

The duration of clinical study participation is estimated to be:

- approximately 4 weeks for subjects with normal renal function (up to 3 weeks screening period followed by 8 days for treatment phase and follow-up)
- approximately 6 weeks for subjects on hemodialysis (up to 3 weeks screening period followed by at least 16 days for treatment phase and follow-up)

The study starts with first subject signing informed consent (FSI) and ends with the last subject undergoing last visit (LSO).

9.2 Discussion of study design, including the choice of control groups - amended[8]

According to relevant guidelines (see Section 7.5) the chosen design was considered to be adequate to achieve the study objectives.

Estimated glomerular filtration rate (eGFR) will be based on the MDRD equation (see Section 9.5.1.3 for details). In this clinical study protocol, the eGFR of $\geq$80 mL/min/1.73 m^2 was chosen as the cut-off point as elderly healthy subjects will be included in the study.

To study the effect of hemodialysis, PK of the study drug should be studied under both dialysis and non-dialysis conditions. Therefore, subjects on hemodialysis will receive 2 treatments (24 h before start of dialysis and immediately before dialysis). A fixed sequence design has been chosen as it fits best to clinical practice.

Plasma migalastat $t_{1/2}$ increases with worsening renal function (up to 32.3 h in subjects with severe renal impairment). As the study drug is considered to be dialyzable (see Section 7.3), a washout phase of at least 8 days between administrations in subjects on hemodialysis is considered to be sufficient.

Two methods of hemodialysis are investigated in this study (standard hemodialysis, hemodiafiltration) to ensure that the data collected reflect the standard clinical practice. The 2 methods are similar to each other with regard to elimination of small molecules. Therefore, because of the low molecular weight of migalastat (163.17 Dalton), it is not anticipated that percent of the migalastat dose recovered will significantly differ between the 2 methods.

Subjects with normal renal function will be matched 1:1 to subjects in the ESRD group based on their age, body weight, and sex (for details, see Section 9.1). In each group at least 2 subjects of each sex will be enrolled.

Male and female subjects will be enrolled in this study. Galafold® is indicated for male and female subjects with Fabry disease. As expressed in ICH guidelines, it should be a principle that "patients entering clinical studies should be reasonably representative of the population that will later be treated by the drug". Overall, it is

[8] Section updated via Amendment 2 to clarify the enrollment of subjects on standard hemodialysis and hemodiafiltration

deemed as important to continue to document safety of migalastat in both females and males, in future studies.

Food reduces the bioavailability of migalastat by approximately 40%. Therefore, subjects will be fasted overnight and until 2 h after administration of migalastat.

For justification of dose see Section 7.4.

For justification of 24 h time difference to dialysis, see Section 9.4.4.

Subjects will be dosed in a staggered manner. For details, see Section 9.1.

9.3 Selection of study population - amended

In total, 6 subjects with ESRD on hemodialysis (3 subjects on standard hemodialysis, 3 subjects on hemodiafiltration)[9] and 6 matched subjects with normal renal function will participate in the treatment phase of the clinical study.

Only subjects meeting all inclusion (Section 9.3.1) and none of the exclusion criteria (Section 9.3.2) will be included into the treatment phase. Deviations from inclusion and exclusion criteria are not allowed. Adherence to the criteria as specified in this protocol is essential.

The criteria will be assessed at screening and a re-check will be performed on Day -1 (subjects on hemodialysis: Day -1 of the first period). For assessment of eligibility, examinations as described in Section 9.5.3 will be performed before administration of the investigational medicinal product (IMP).

9.3.1 Inclusion criteria

9.3.1.1 Subjects with normal renal function

Subjects must fulfill all of the following criteria to be eligible for participation in the treatment period.

		Screening	Day -1
1.	Healthy subjects aged 18 to 79 years	X	
2.	eGFR according to MDRD equation of $\geq$ 80 mL/min/1.73 m^2 at the screening visit	X	
3.	Subject is willing and able to provide written informed consent and authorization for use and disclosure of Personal Health Information (PHI) or research related health information.	X	
4.	If of reproductive potential, both male and female subjects agree to use a medically accepted method	X	X

[9] Included via Amendment 2

		Screening	Day -1
	of contraception during the study and for up to 30 days after dosing with migalastat.<br>Highly-effective methods of birth control are defined as those which result in a low failure rate, i.e. less than 1% per year, when used consistently and correctly (e.g., combination of intrauterine device and condom, sexual abstinence).<br>For definition of childbearing potential see Section 7.4.		
5.	Body mass index (BMI) within 18.0 to 35.0 kg/m^2.	X	X
6.	In general good physical health as determined by medical and surgical history, physical examination, 12 lead ECG, vital signs, and clinical laboratory tests.	X	X
7.	Having had no febrile or infectious illness for at least 7 days prior to administration of the IMP of the study.		X
8.	Normal blood pressure (Systolic Blood Pressure (SBP) ≥90, ≤145 mmHg; Diastolic Blood Pressure (DBP) ≥55, ≤89 mmHg) measured after 5 min rest in supine position.	X	X
9.	A pulse rate at rest of ≥50 and ≤99 b/min measured after 5 min rest in supine position.	X	X
10.	ECG recording without clinically significant abnormalities.	X	X

9.3.1.2 Subjects with ESRD - amended

		Screening	Day -1*
11.	Male or female Non-Fabry subjects aged 18 to 79 years	X	
12.	eGFR according to MDRD equation of < 15 mL/min/1.73 m^2 at the screening visit	X	
13.	Subject is willing and able to provide written informed consent and authorization for use and disclosure of PHI or research related health information or, in case of subjects with ESRD, has	X	

		Screening	Day -1*
	a legally authorized representative who has given written informed consent.		
14.	Subject receives hemodialysis (standard hemodialysis or hemodiafiltration)[10] (at least 4 h every 72 h).	X	X
15.	Subject has been stable on his/her dialysis regimen for at least 2 months.	X	X
16.	If of reproductive potential, both male and female subjects agree to use a medically accepted method of contraception during the study and for up to 30 days after the (last) dose of migalastat.<br><br>Highly-effective methods of birth control are defined as those which result in a low failure rate, i.e. less than 1% per year, when used consistently and correctly (e.g., combination of intrauterine device and condom, sexual abstinence).<br><br>For definition of childbearing potential see Section 7.4.	X	X
17.	BMI within 18.0 to 35.0 kg/m^2.	X	X
18.	Having had no febrile or infectious illness for at least 7 days prior to (first) administration of the IMP of the study.		X
19.	SBP ≥90, ≤179 mmHg; DBP ≥55, ≤100 mmHg) measured after 5 min rest in supine position	X	X
20.	A pulse rate at rest of ≥50 and ≤99 b/min measured after 5 min rest in supine position.	X	X
21.	ECG recording without clinically significant abnormalities.	X	X

*On Day -1 of the first treatment period only

9.3.2 Exclusion criteria

9.3.2.1 Subjects with normal renal function

Subjects meeting any of the following criteria must not be enrolled in the study:

[10] Added via Amendment 2

		Screening	Day -1
	**Lifestyle restrictions**		
1.	Demonstrating excess in xanthine consumption (more than 5 cups of coffee or equivalent per day).	X	
2.	More than moderate alcohol consumption (>35 g of ethanol regularly per day or >245 g regularly per week).	X	
3.	Any history of alcohol or drug abuse.	X	
4.	More than moderate smoker (>10 cigarettes/day).	X	
5.	Consumption of xanthine-containing food or beverages within 48 h before dosing.		X
6.	Consumption of grapefruit-containing food and beverages within 1 week before dosing.		X
7.	Positive urine drug screen.	X	X
8.	Positive alcohol breath test.	X	X
	**Prior medication**		
9.	Use of any medication (self-medication or prescription medication) within 4 weeks before dosing (or at least 10 times the respective elimination half-life, whichever is longer) (except hormonal contraceptives and paracetamol)	X	X
	**Medical and surgical history**		
10.	The subject has undergone kidney transplantation	X	
11.	Any history of allergy or sensitivity to migalastat (including excipients) or other iminosugars (e.g., miglustat, miglitol) or to any of the excipients (pregelatinized starch and magnesium stearate, both of vegetable origin).	X	
12.	Any history of drug hypersensitivity, asthma, urticaria or other severe allergic diathesis as well as current hay fever.	X	
13.	Any intercurrent illness or condition that may preclude the subject from fulfilling the protocol requirements or suggests to the investigator that the potential subject may have an unacceptable risk by	X	X

		Screening	Day -1
	participating in this study.		
14.	Any history of chronic or recurrent metabolic, renal, hepatic, pulmonary, gastrointestinal, neurological (esp. history of epileptic seizures), endocrinological, immunological, psychiatric or cardiovascular disease, myopathies, and bleeding tendency.	X	
15.	Demonstrating any active physical disease, acute or chronic.	X	X
	**Laboratory examinations**		
16.	Laboratory values outside the reference range that are of clinical relevance in the opinion of the investigator (e.g., suggesting an unknown disease and requiring further clinical evaluation assessed by the investigator) especially aspartate aminotransferase (AST), alanine aminotransferase (ALT), gamma glutamyl transpeptidase (gamma-GT).	X	X
17.	Positive test for human immunodeficiency virus (HIV) antibodies or HIV-1 p24-antigen	X	
18.	Positive Hepatitis B-virus surface antigen (HBsAg) test.	X	
19.	Positive Anti-hepatitis C-virus antibodies (Anti-HCV) test.	X	
	**Other**		
20.	The subject is treated or has been treated with another investigational drug within 30 days of study start.	X	X
21.	Unable to comply with study requirements, or deemed otherwise unsuitable for study entry, in the opinion of the investigator.	X	X
22.	Female subject is pregnant or breastfeeding.	X	X
23.	Blood donation within 30 days before signing informed consent to this study.	X	

9.3.2.2 Subjects with ESRD

Subjects meeting any of the following criteria must not be enrolled in the study:

		**Screening**	**Day -1***
	**Lifestyle restrictions**		
24.	Demonstrating excess in xanthine consumption (more than 5 cups of coffee or equivalent per day).	X	
25.	More than moderate alcohol consumption (>35 g of ethanol regularly per day or >245 g regularly per week).	X	
26.	Any history of alcohol or drug abuse.	X	
27.	More than moderate smoker (>10 cigarettes/day).	X	
28.	Consumption of xanthine-containing food or beverages within 48 h before (first) dosing.		X
29.	Consumption of grapefruit-containing food and beverages within 1 week before (first) dosing.		X
30.	Positive urine drug screen.	X	X
31.	Positive alcohol breath test.	X	X
	**Prior medication**		
32.	Use of any medication (self-medication or prescription medication) within 4 weeks before first dosing (or at least 10 times the respective elimination half-life, whichever is longer) (except hormonal contraceptives, paracetamol, and those drugs the renally impaired subject is currently taking for treatment of the renal or concomitant disease).	X	X
33.	Change of the chronic medication less than 14 days prior to (first) dosing.	X	X
34.	Requires treatment with Glyset® (miglitol) or Zavesca® (miglustat)	X	X
	**Medical and surgical history**		
35.	The subject has undergone kidney transplantation	X	
36.	Any history of allergy or sensitivity to migalastat (including excipients) or other iminosugars (e.g., miglustat, miglitol) or to any of the excipients	X	

		Screening	Day -1*
	(pregelatinized starch and magnesium stearate, both of vegetable origin).		
37.	Any history of drug hypersensitivity, asthma, urticaria or other severe allergic diathesis as well as current hay fever.	X	
38.	Any intercurrent illness or condition that may preclude the subject from fulfilling the protocol requirements or suggests to the investigator that the potential subject may have an unacceptable risk by participating in this study.	X	X
39.	Any severe or unsuitable concomitant medical condition (cardiovascular, neurological, hepatic, metabolic, hematological, immunological, pulmonary, or gastrointestinal disorder). The medical monitor or designee should be contacted in case of indistinct medical condition to discuss the stability of a subject’s medical condition(s) and the potential impact of the condition(s) on study participation.	X	X
	**Laboratory examinations**		
40.	Any clinically significant abnormal laboratory value(s) and clinically significant ECG findings not due to the underlying disease. In case of unclear or significant findings the medical monitor or designee should be contacted to discuss the stability of a subject’s medical condition(s) and the potential impact of the condition(s) on study participation.	X	X
41.	Positive test for HIV antibodies or HIV-1 p24-antigen	X	
42.	Positive HBsAg test.	X	
43.	Positive Anti-hepatitis C-virus antibodies (Anti-HCV) test.	X	
	**Other**		
44.	The subject is treated or has been treated with another investigational drug within 30 days of study start.	X	X
45.	Unable to comply with study requirements, or deemed otherwise unsuitable for study entry, in the opinion of the investigator.	X	X

		Screening	Day -1*
46.	Female subject is pregnant or breastfeeding.	X	X
47.	Blood donation within 30 days before signing informed consent to this study.	X	

*On Day -1 of the first treatment period only

9.3.3 Discontinuation of subjects from treatment or assessment

Subjects who withdraw from study participation before enrollment are considered screening failures.

A subject may be withdrawn from the study before completion of all study procedures for the following reasons:

- Subjects may withdraw their informed consent at any time during the study without giving reasons and without disadvantages. They may withdraw their consent for processing of data (except the data listed in § 40 (2a) AMG) derived from study participation, any privacy waivers, and participation in study procedures or active treatment.
- The investigator may decide to withdraw a subject if further participation in the study may affect the subject's safety or well-being, or if the subject developed any violation of inclusion or exclusion criteria during the study, or if the subject does not comply with the study requirements or instructions from study site personnel. If not due to immediate hazards, the investigator will consult the sponsor before withdrawal of a subject.
- The sponsor may decide to withdraw a subject if further participation in the study may affect the subject's safety or well-being, or if the subject developed conditions which would have prevented his/her entry into the study according to the inclusion or exclusion criteria, or if the subject does not comply with the study requirements or instructions from study site personnel.
- A subject may discontinue without giving notice or without action. A subject will be considered lost to follow-up if he/she repeatedly fails to return for scheduled visits and is unable to be contacted by the study site.

The investigator will document the primary reason for withdrawal and the date of discontinuation in the case report form (CRF). The investigator will make any reasonable effort to follow-up the subject's safety.

9.3.4 Subject replacement - amended

Subjects with ESRD are enrolled until 3 subjects on standard hemodialysis and 3 subjects on hemodiafiltration with both periods valid for PK analyses are available. For each of these subjects a matched healthy control is included.[11]

9.3.5 Premature discontinuation of the study

The sponsor has the right to terminate the entire study or parts thereof at any time. This may especially be the case if any information becomes known that renders the risk-benefit ratio of this study unfavorable or if the conduct of the study does not suggest a proper or correct completion within a reasonable period.

The investigator has the right to close the study center at any time.

A premature termination must be discussed between the involved parties before becoming effective.

There are no statistical criteria for study termination.

9.3.6 Subject identification

Each subject will be identified in the subject database of the study site by a unique subject identification number (PID) and a screening number. When the subjects are enrolled into the treatment phase of the study, they will be assigned to a study – specific 3-digit treatment number (sequential subject numbering starting with 001). This means that only pseudonymized data will be transferred to the sponsor or body appointed by him. The screening number will be used to identify the subject throughout the study (i.e., on case report forms and all laboratory samples). The treatment number is also used to label laboratory samples after enrollment.

The subjects will have site specific identification cards for identification during study participation. The subjects are obliged to wear these identification cards visibly during their stay at the study center. The study personnel will check the cards before each administration, measurement or sampling procedure.

9.4 Treatments

9.4.1 Treatments administered

The treatments to be administered during the study are displayed in Table 9-2.

Table 9-2: Treatments administered

Renal function	Dose of migalastat HCl	Formulation / Route of administration	Frequency of administration	No. of subjects treated

[11] Updated via Amendment 2 to reflect the enrollment of subjects on standard hemodialysis and hemodiafiltration

ESRD on hemodialysis	150 mg	Capsule / oral	2 single doses (Period 1: 24 h before start of dialysis Period 2: immediately before start of dialysis)	6
Normal renal function	150 mg	Capsule / oral	Single dose	6

ESRD: end-stage renal disease

9.4.2 Identity of investigational medicinal products

The details of the IMP are presented in Table 9-3.

Table 9-3: Identity of test drug

	**Test (AT1001)**
Name:	Migalastat HCl
Active ingredient (INN):	Migalastat
Formulation:	Capsule
Dose:	150 mg migalastat HCl (equivalent to 123 mg migalastat free base) corresponding to one capsule
Mode of administration:	Oral
Manufacturer/Marketing Authorization Holder:	Almac Pharma Services Limited, Armagh, United Kingdom/Amicus Therapeutics UK Ltd, Buckinghamshire, United Kingdom

9.4.2.1 Supply, packaging, and labeling of investigational medicinal products

The sponsor will ensure that all IMPs are characterized and manufactured in accordance with any applicable requirements of Good Manufacturing Practice (GMP) and regulatory requirements.

After the study protocol is approved by the IEC and regulatory authorities, the sponsor will supply the investigator and study sites with all study medication, together with all relevant documentation including a description of the storage conditions.

The sponsor will maintain a complete record of batch numbers and expiry dates of all IMP as well as the labels of all IMP in the Trial Master File.

The investigator will maintain, amongst other documents, a record of the batch numbers and expiry dates of all IMP as well as the labels of the IMP received at the study site in the Investigator Site File.

9.4.2.2 Drug accountability

The sponsor will maintain records that document shipment, receipt, dispensation, return, and destruction of all IMP in the Trial Master File.

The sponsor is responsible for the storage of retention samples of the IMP according to applicable laws and guidelines.

The investigator will maintain records of IMP accountability in the Investigator Site File (ISF) for all IMP provided by the sponsor.

After the end of the study, the investigator will return all used and unused IMP to the sponsor or destroy used and unused IMP according to written agreement with the sponsor.

9.4.3 Method of assigning subjects to treatment groups

After completion of all inclusion/exclusion criteria evaluations, the eligible subjects will be assigned to one of the 2 treatment groups according to their renal function characterized by eGFR according to MDRD equation at screening. For details, please see Section 9.1.

9.4.4 Selection of doses on the study

For justification of the 150 mg dose, see Section 7.4.

A time difference of 24 h between dosing and start of dialysis has been chosen for the group of subjects on hemodialysis based on the following rationale:

Migalastat HCl 150 mg every other day is the currently labeled dose for Fabry patients with amenable *GLA* variants who have eGFR ≥ 30 mL/min/1.73 m^2. In these Fabry patients with sufficient renal function, migalastat is typically absorbed within 3 h of dosing with a terminal half-life of approximately 4 h. Rapid plasma and tissue clearance allows for the majority of the migalastat dose to be renally excreted within approximately 24 h post-dose. As a pharmacological chaperone (PC) that binds and stabilizes the lysosomal enzyme α-Gal A, adequate time must be allowed for uptake and trafficking of α-Gal A to the lysosome, followed by dissociation and clearance of the inhibiting, but stabilizing PC, migalastat. In an end-stage renal disease scenario, where renal function has been replaced by dialysis with a semipermeable membrane, dosing with migalastat must be precisely timed around dialysis treatments to allow for adequate time for uptake and tissue distribution before the start of dialysis. For that reason, migalastat HCl, and the labeled dose of 150 mg will be administered 24 h before the start of dialysis in Period 1 of this study.

9.4.5 Selection and timing of dose for each subject

Regarding dose, route / mode of administration, formulations and duration of treatments, refer to Section 9.4.1 and Section 9.4.2.

Regarding the selection of dose, refer to Section 9.4.4.

Subjects with ESRD on hemodialysis will receive 2 single oral doses of 150 mg migalastat HCl together with 240 mL (240 mL may be reduced to 150 mL if required) of non-sparkling water at room temperature separated by a washout phase of at least 8 days. One dose will be administered 24 h before start of dialysis

(Period 1) and one dose will be administered immediately before start of dialysis (Period 2).

Subjects with normal renal function will receive one single oral dose of 150 mg migalastat HCl together with 240 mL of non-sparkling water at room temperature.

The study drug will be administered after an overnight fast.

A standardized snack will be given 2 h after administration, a standardized lunch will be given approximately 6 h after administration, and a standardized dinner will be given approximately 10 h after administration.

On the dosing day water intake is restricted from 1 h before dosing until 1 after dosing (except at dosing time).

On the other in-house days standardized meals and liquids will be served at customary times.

Subjects are not allowed to consume any food and beverages not provided by the staff of CRS-Kiel during the in-house study days. Subjects with ESRD may have additional meals during dialysis as appropriate which will be documented in the CRF.

Dietary restrictions are described in Section 9.4.10.

9.4.6 Blinding

The study will be performed non-blinded, as all subjects will receive the same treatment and renal function cannot be blinded.

9.4.7 Prior and concomitant therapy

For restrictions on prior therapy, see Section 9.3.2.

During the clinical study, concomitant medication/therapy is generally not allowed (with the exception of hormonal contraceptives). A symptomatic treatment of AEs (e.g., treatment of headache with 500 mg paracetamol) or topical drugs may be allowed by the discretion of the investigator. Any concomitant treatment has to be documented in the eCRF. Drugs the subjects with renal impairment are taking for treatment of the renal or concomitant disease are allowed.

Subjects with renal impairment with a change of medication or dose regimen of medically required medication within 2 months prior to first dosing will not be included into the study. A change of medication or dosing within the study will have to be avoided unless it is medically necessary.

9.4.8 Treatment compliance

Administration of the IMP will be performed under the supervision of the investigator or his designee and will be followed by a mouth and hand check.

Furthermore, treatment compliance will be controlled by evaluation of relevant drug profiles in dialysate (subjects with ESRD only), plasma, and urine after administration.

9.4.9 Treatment after end of study participation

Medical care after discharge from the study will be provided by the subject's family practitioner.

9.4.10 Restrictions and precautions

Restrictions during the study are provided in Table 9-4.

Table 9-4: Study restrictions

Substance	Restriction
Smoking	Smoking is not allowed on the dosing days until 10 h after dosing.
Alcohol	Not allowed from 48 h before the (first) drug administration until follow-up visit.
Xanthine-containing food and beverages	Not allowed from 48 h before drug administration and during the in-house phase.
Food	Standardized meals during their stay at the study site. Subjects with ESRD may have additional meals during dialysis as appropriate.
Food and beverages containing grapefruit	Not permitted from 1 week before the drug administration and during the in-house phase.
Exposure to sunlight, solarium	To be avoided from Day-1 (ESRD subjects: of the first treatment period) until follow-up visit.
Physical activity	Usual activities permitted (subjects should refrain from strenuous exercise during the study).

9.5 Population characteristics, pharmacokinetic and safety endpoints

9.5.1 Populations characteristics

9.5.1.1 Demographic characteristics

Demographic characteristics will include the following data:

Age (in years at screening), sex, body height in centimeters and body weight in kilograms.

The body mass index will be calculated as body weight divided by the square height in meter [kg/m^2]. The height at screening will be used for calculation of the body mass index on Day -1.

9.5.1.2 Medical and surgical history

Medical and surgical history will focus on current or past abnormalities or diseases of the following systems: special senses, cardiovascular, respiratory, gastrointestinal, hepatic biliary, genitourinary/reproductive, renal, endocrine/metabolic, musculoskeletal, hematologic/lymphatic, neurologic/psychiatric, dermatologic, immunologic, and infectious disease, bleeding tendency, and allergy/drug sensitivity.

9.5.1.3 Lifestyle and other baseline characteristics

A detailed history will be taken for smoking and alcohol habits, xanthine consumption, drug abuse, and previous/present medication/therapy.

The eGFR will be calculated from the creatinine concentration measured in serum according to the following formula (MDRD formula):

$$\text{eGFR [mL/min/1.73 m}^2\text{]} = 175 \times [\text{Creatinine in Serum (mg/dL)}]^{-1.154} \times [\text{Age (years)}]^{-0.203} \times (0.742 \text{ if female}) \times (1.212 \text{ if African American})$$

9.5.2 Pharmacokinetics

9.5.2.1 Sample collection and sample handling

Blood sampling

Blood samples for the determination of migalastat concentrations in plasma will be collected at the time points given in Section 2.2.

For accepted deviations for PK blood sampling see Section 2.2. Deviations from these timeframes will be commented.

The time point for blood collection takes priority over any other scheduled clinical study activities. Where other activities are scheduled together with blood collection, these will be performed before or after blood collection and blood sampling will be done exactly at the scheduled time point.

Details about the collection, sample handling and processing, storage and shipment of samples will be provided in the laboratory manual and filed in the ISF.

Urine sampling (if able to produce urine)

Urine for the determination of migalastat concentrations will be collected at the intervals given in Section 2.2, if subjects are able to produce urine. A urine sample will be taken for urine migalastat determination from each collection.
The subjects will be instructed to empty their bladder at the beginning and at the end of each collection interval. The procedure should be arranged in such a manner, that the urine of each subject will be collected as completely as possible. Suitable containers will be provided and labeled unambiguously. The following data will be recorded per subject and collection interval: urine weight (g), specific gravity, urine volume (mL, calculated from weight and specific gravity).

Details about the collection, sample handling and processing, storage and shipment of samples will be provided in the laboratory manual and filed in the ISF.

Dialysate sampling in ESRD subjects on hemodialysis

Dialysate for the determination of migalastat concentrations will be collected at the time points given in Section 2.2. Dialysate volumes will be measured.

Dialysate volume, day, start and stop times of dialysis, flow rate of the dialysis, blood flow, dry weight, dialysate fluid, ultrafiltration and eventual changes therein, dialysis machine, aditus, and information on filter and membrane will be documented in the source data.

Dialysate volume, day, start and stop times of dialysis, flow rate of the dialysis, and blood flow will additionally be documented in the eCRF.

Details about the collection, sample handling and processing, storage and shipment of samples will be provided in the laboratory manual and filed in the ISF.

9.5.2.2 Bioanalytical measurements

Bioanalysis of migalastat in plasma, urine and dialysate will be determined by validated Liquid Chromatography Tandem Mass Spectrometry (LC/MS-MS) assay methods.

The bioanalytical procedures of the laboratories are in accordance with applicable regulatory requirements.

9.5.2.3 Pharmacokinetic variables

The PK parameters will be calculated using non-compartmental procedure.

Based on the concentration time data, the following PK parameters will be calculated:

<u>Migalastat in plasma</u>

- maximum observed concentration between time zero to 24h ($C_{max,0-24}$)
- time to maximum concentration (t_{max})
- apparent terminal elimination half-life ($t_{1/2}$)
- area under the concentration-time curve from time zero to the last measurable concentration (AUC_{0-t})
- extrapolated to infinity ($AUC_{0-\infty}$) (subjects with normal renal function, Period 2 of ESRD subjects)
  If >50% of the area is extrapolated, then $AUC_{0-\infty}$ as well as other extrapolated parameters will not be reported for that individual
- area under the concentration-time curve from time zero to 24h (AUC_{0-24})
- apparent plasma clearance (CL/F)
- apparent terminal phase volume of distribution (V_z/F)

Migalastat in urine

- total amount excreted (A_e)
- fraction of the dose recovered in urine (F_e)
- renal clearance (CL_r)

Migalastat in dialysate

- dialysis clearance (C_LD)
- volume of dialysate collected during the interval (VD)
- migalastat concentration in dialysate (CD)
- amount recovered in dialysate (A_eD)
- fraction of the dose recovered in dialysate (F_eD)
- extraction coefficient or ratio of dialysis clearance and blood flow may be estimated

9.5.3 Safety pharmacology

The measurements performed by CRS-Kiel will be carried out according to CRS Standard Operating Procedures (SOPs).

Measurements will be performed at the time points given in the flow chart.

9.5.3.1 Physical examination

The physical examination will comprise physical inspection of general condition/psyche, skin, lymph nodes, head (including eyes, ears, mouth) and neck/thyroid gland, lungs, heart, abdomen, kidneys, musculoskeletal system, neurological system, vascular system.

9.5.3.2 Vital signs

Blood pressure (systolic and diastolic), pulse rate, and respiratory rate will be measured after at least 5 min rest in supine position. All recordings should be made using the same type of blood pressure recording instrument on the same arm, if possible (on both arms at screening if possible, for inclusion criteria the higher result have to be taken).

Aural body temperature will be determined.

9.5.3.3 ECG recording

A standard 12-lead ECG will be recorded after at least 5 min rest in supine position using the leads according to Einthoven and Goldberger as well as 6 precordial leads according to Wilson[7].

Printouts for each ECG (at least three complexes for each standard lead) will include date and time of recording, subject's identification, physician's evaluation, and physician's initials. The date and time of the ECG recordings as well as the overall ECG evaluation and potential abnormal findings of the ECG recordings will be transcribed into the subject's eCRF.

9.5.3.4 Safety laboratory examinations

Laboratory analyses, except for urinalysis (dipstick), drug screening and alcohol breath test will be carried out by the contract laboratory (Laboratorium Klinische Forschung GmbH, Lise-Meitner-Strasse 25 - 29, 24223 Schwentinental, Germany). This laboratory is board-accredited by the "Deutsche Akkreditierungstelle GmbH (DAkkS)" according to DIN EN ISO 15189 and participates in routine tests for analytical quality.

Urinalysis (dipstick), drug screening and alcohol breath test will be carried out at the study site.

Blood samples for safety laboratory examination will be taken in fasted state.

Clinical chemistry parameters
Albumin, amylase, P-amylase, bicarbonate, total and direct bilirubin, glucose (fasting), AST, ALT, alkaline phosphatase (AP), lactate dehydrogenase (LDH), creatine phosphokinase (CK), creatinine, urea, uric acid, potassium, sodium, calcium, chloride, total protein, cholesterol (high-density lipoprotein [HDL], low-density lipoprotein [LDL], total), cholinesterase, iron, lipase, magnesium, phosphate, triglycerides

At screening only: FSH and estradiol in females who are post-menopausal < 2 years for confirmation of postmenopausal status (see Section 7.4).

Hematology parameters
Hematocrit, hemoglobin, mean corpuscular volume (MCV), mean corpuscular hemoglobin (MCH), mean corpuscular hemoglobin concentration (MCHC), erythrocytes, leukocytes with differential count (neutrophils, lymphocytes, monocytes, eosinophils, basophils), and platelet count.

Serology parameters
HIV-1/2 antibodies/HIV-1 p24-antigen, HBsAg, and Anti-HCV.

Urinalysis parameters (if able to produce urine)
Leukocytes, nitrite, pH, protein, glucose, ketone, urobilinogen, bilirubin, blood (dipstick).

Microscopic examination of the sediment will be performed if considered necessary by the investigator.

Urine drug screen (if able to produce urine)
A urine drug screen will be performed using standard urine drug tests (proof of amphetamines, barbiturates, benzodiazepines, cannabinoids, cocaine, methadone, methamphetamine/ecstasy, morphine/opiates, tricyclic antidepressants, phencyclidine).

Alcohol breath test
Alcohol testing will be performed using a standard alcohol breath test.

Pregnancy test in females of childbearing potential
The serum pregnancy tests will be performed in females of childbearing potential.

9.5.3.5 Adverse events

For definitions, documentation, and rating of AEs refer to Section 19.4.1.

9.5.3.6 Pregnancies

For reporting and documenting pregnancies, refer to Section 19.5.1.

9.6 Appropriateness of measurements

All measurements are carried out according to standard methods.

9.7 Statistical methods and determination of sample size

9.7.1 Statistical and analytical plans

The biometrical evaluation will be carried out by the Department Clinical Data Management of CRS-Mannheim using Statistical Analysis System (SAS) software, Version 9.3 or higher of the SAS System for windows and Phoenix WinNonlin Version 6.4 or higher. Copyright, SAS Institute Inc. SAS and all other SAS Institute Inc. product or service names are registered trademarks or trademarks of SAS Institute Inc., Cary, NC, USA.

9.7.1.1 General statistical considerations - amended[12]

The following sections describe an overview of the planned statistical methods. A statistical analysis plan (SAP) will be written in which the details of the statistical methods will be described. The SAP will be finalized before database lock. Any deviations from the originally planned statistical analysis or SAP will be described and justified in the clinical study report. Changes known at the time of SAP preparation will also be described in the SAP.

A data review meeting will be held before database hard lock. Protocol deviations will be reviewed during data review meeting. Furthermore, assignment of subjects to the analysis set will be performed.

[12] Section updated via Amendment 2 to clarify the enrollment of subjects on standard hemodialysis and hemodiafiltration

Documentation of subjects who did not meet the entry criteria (screening failures) will be collected and filed only in their electronic case report forms (eCRFs); the data will not be listed. The following data will be recorded for screening failures: date of informed consent, demographic data, reason for discontinuation.

Subjects with normal renal function are subjects who fulfill the matching criteria for age, body weight, and gender.

All measured variables and derived PK parameters will be listed individually and, if appropriate, tabulated by descriptive statistics for ESRD subjects on standard hemodialysis, ESRD subjects on hemodiafiltration, subjects with normal renal function matched to ESRD subjects on standard hemodialysis, and subjects with normal renal function matched to ESRD subjects on hemodiafiltration. Figures will be provided as appropriate.

For interference statistical analyses (PK dependent on eGFR) data will be pooled for ESRD subjects and for subjects with normal renal function.

Descriptive statistics will provide the number of observation and the absolute and relative frequency of categorical variables. For the continuous variables, number of observations, arithmetic mean, standard deviation, coefficient of variation (if appropriate), median as well as minimum and maximum will be given.

Descriptive statistics of PK parameters and concentrations will additionally include the geometric mean and the geometric standard deviation. If a zero occurs in a sample, the corresponding geometric mean and geometric standard deviation will be computed, omitting this value.

As appropriate, summaries will be grouped for ESRD subjects on standard hemodialysis, ESRD subjects on hemodiafiltration, subjects with normal renal function matched to ESRD subjects on standard hemodialysis, subjects with normal renal function matched to ESRD subjects on hemodiafiltration, and time of assessment.

9.7.1.2 Analysis sets

Safety Analysis Set

All enrolled subjects who received at least one dose of the study medication will be included in the safety evaluation and the corresponding population.

Pharmacokinetic Analysis Set

All subjects for whom the primary PK parameters of migalastat can be derived from at least one treatment period will be included in the PK population.

9.7.1.3 Population characteristics - amended[13]

Demographic data

Demographic variables and baseline characteristics will be listed and summarized for ESRD subjects on standard hemodialysis, ESRD subjects on hemodiafiltration, subjects with normal renal function matched to ESRD subjects on standard hemodialysis, subjects with normal renal function matched to ESRD subjects on hemodiafiltration, and overall for the safety and the PK population (provided separately if both populations are different). The descriptive statistics will include number of observations, mean, standard deviation, median, minimum and maximum for continuous variables and number of observations and their percentages for categorical parameters.

Consumption history

Data about smoking and alcohol habits, xanthine consumption and drug abuse will be listed and summarized for ESRD subjects on standard hemodialysis, ESRD subjects on hemodiafiltration, subjects with normal renal function matched to ESRD subjects on standard hemodialysis, subjects with normal renal function matched to ESRD subjects on hemodiafiltration, and overall both for the safety and the PK population, if different.

Medical and surgical history

Medical and surgical history results will be listed and coded using Medical Dictionary for Regulatory Activities (MedDRA). Coding will be performed with the dictionary version currently used by CRS-Mannheim at time point of study start. The dictionary version will not be changed during the study. Coding terms and investigator terms will be given in the listings.

Prior and concomitant medications

Prior and concomitant medications will be listed and coded using the World Health Organization drug dictionary. Coding terms and investigator terms will be given in the listings. Coding will be performed with the dictionary version currently used by CRS-Mannheim at time point of study start. The dictionary version will not be changed during the study.

eGFR

Data will be listed and summarized for ESRD subjects on standard hemodialysis, ESRD subjects on hemodiafiltration, subjects with normal renal function matched to ESRD subjects on standard hemodialysis, and subjects with normal renal function matched to ESRD subjects on hemodiafiltration for the safety and the PK population, if different.

[13] Section updated via Amendment 2 to clarify the enrollment of subjects on standard hemodialysis and hemodiafiltration

9.7.1.4 Pharmacokinetics

Plasma concentrations

Individual concentrations of migalastat in plasma will be displayed graphically and listed on the original as well as on a logarithmic scale.

For descriptive statistics of plasma migalastat concentrations, values below lower limit of quantification (LLOQ) will be assigned a value of 0. Descriptive statistics of concentrations will be calculated if at least 1/2 of the individual data points have been measured equal or above LLOQ.

Individual data points in the lag-time between time zero and the first quantifiable concentration will be displayed as 0 on the linear concentration scale and they will not be displayed on the logarithmic scale.

Urine concentrations

Individual concentrations of migalastat in urine will be listed and summarized with descriptive statistics. Figures will be provided by group.

Dialysate concentrations

Individual concentrations of migalastat in dialysate will be listed and summarized with descriptive statistics. Figures will be provided by group.

Pharmacokinetic characteristics in plasma

Calculation of the PK characteristics will be based on actual blood sampling times [h] (relative to the corresponding administration time) rounded to 2 decimal digits and negative pre-dose times set to zero.

For calculation of the PK parameters the following rules will be applied: At time points in the lag-time between time zero and the first quantifiable concentration, concentrations below LLOQ will be calculated as zero. Concentrations below LLOQ between 2 quantifiable concentrations will be set to missing. Trailing concentrations below LLOQ will set to missing.

Descriptive statistics of PK parameters will be calculated.

For t_{max} frequency tables will be drawn based on the nominal time of t_{max}.

Figures will be provided.

Individual data points in the lag-time between time zero and the first quantifiable concentration will be displayed as 0 on the linear concentration scale and they will not be displayed on the logarithmic scale.

Correlation with eGFR

eGFR values (x-axis) will be plotted against the respective main PK parameters (y-axis).

Pharmacokinetic characteristics in urine and dialysate

Urine and dialysate PK will be based on concentrations and volumes.

Figures will be provided.

Comparisons

For the group of ESRD subjects under hemodialysis it is an intra-individual situation. Each subject serves as his own control (with versus without dialysis). Tables will display the individual data paired for each subject.

The comparison of ESRD subjects with healthy subjects will be handled as an intra-individual situation as well, because the subjects match for demographic characteristics. However, display of characteristics (PK and others) will be given both, inter- and intra-individually.

9.7.1.5 Safety and tolerability

Results of all safety measurements will be listed individually and, as appropriate, summarized.

Adverse events

All AEs will be listed together with information on onset, duration, severity, seriousness, relationship to the IMP, outcome and action taken.

Adverse events will be coded using MedDRA. Coding will be performed with the dictionary versions currently used by CRS-Mannheim at time point of study start. Dictionaries will not be changed during the study. Coding terms and investigator terms will be given in the listings.

An AE will be referred to the treatment and time point after which it occurred, i.e. any AE occurring before (first) dosing will be counted as baseline complaint/ pre-treatment AE and an event will only be considered as treatment-emergent adverse event (TEAE) if occurring after or at (first) dosing.

Only TEAEs will be analyzed using summary tables. Frequency tables for TEAEs will be given by system organ class and preferred term and will be provided by severity and relationship to IMP.

Other safety assessments

Descriptive statistics will be calculated for laboratory parameters, vital signs including body temperature, and ECG parameters. Changes from baseline will be summarized. Data outside the reference ranges will be flagged in the listings with ‘L’ for low and ‘H’ for high. Information on clinically significance as assessed by an investigator will also be provided in the listings. Additional tables with the number of all abnormal values will be provided for safety laboratory parameters. A separate table will show all abnormal results.

Physical examination findings will be listed.

9.7.1.6 Level of significance

There are no inference statistical analyses. If, with descriptive explorative intention, confidence intervals are calculated, the 95% limits will be presented for differences between groups and the 90% limits for questions of (bio-)equivalence.

9.7.1.7 Procedures in case of missing, unused, and spurious data

In general, missing data will not be replaced or imputed in any way. Only for PK concentrations the rules mentioned above are applied.

For calculation of onset and duration of an AE the following worst case rules will be applied:

- In case of incomplete onset date or onset time the missing information will be imputed such that the onset date or time is minimal [e.g. Onset of AE (date and time): 01JAN2012, 09:UNK h is set to 01JAN2012, 09:00 for calculation of onset and duration].
- In case of incomplete end date or end time the missing information will be imputed such that the duration is maximal [e.g. Onset of AE (date and time): 01JAN2012, 08:00; end of AE (date and time): 01JAN2012, 09:UNK h is set to 01 January 2012, 09:59 for calculation of duration].

9.7.1.8 Interim analysis

An interim statistical analysis will not be performed.

9.7.2 Determination of sample size - amended

The planned sample size of 6 evaluable subjects per renal function group <u>(in the ESRD group on hemodialysis: 3 subjects on standard hemodialysis, 3 subjects on hemodiafiltration)</u>[14] is not based on a power calculation, but is judged based on experience to be adequate to obtain reliable results meeting the objectives of this study.

10 Data handling and record keeping

10.1 Case report forms and recording of data

10.1.1 Electronic records

Electronic records are defined as any combination of text, graphics, data, audio, pictorial, or other information represented in digital form that is created, modified, maintained, archived, retrieved or distributed by a computer system. An eCRF is an auditable electronic record of information that generally is reported to the sponsor on each study subject according to the clinical investigation protocol.

[14] Statement added via Amendment 2

10.2 Data collection (eCRF)

Data will be collected using an eCRF system that is specifically designed for this study. The data collected will be captured in an eCRF system that meets the technical requirements described in 21 CFR part 11. The eCRF system will be fully validated to ensure that it meets the scientific, regulatory, and logistical requirements of the study before it is used to capture data from this study. Before using, all users will receive training on the system and any study specific training. After they are trained, users will be provided with individual system access rights.

Data will be collected at the investigational center by appropriately designated and trained personnel. The eCRF pages must be completed for at least each dosed subject according to the data source. Subject identity should not be discernible from the data provided on the eCRF. Data will be verified using the data source by the study monitor, and reviewed for consistency by Data Management using both automated logical checks and manual review. All data collected will be approved by the investigator at the investigational center. This approval acknowledges the investigator’s review and acceptance of the data as being complete and accurate.

All other Data Management relevant issues will be described within a Data Management Plan.

10.3 Data quality assurance

Clinical Data Management is responsible for the accuracy, quality, completeness, and internal consistency of the data from this study. Data handling, including data quality assurance, will comply with international regulatory guidelines, including ICH GCP guidelines. Data management and control processes specific to this study, along with all steps and actions taken regarding data management and data quality assurance, will be described in a Data Management Plan.

Electronic case report forms will be processed and reviewed for completeness, consistency, and the presence of mandatory values. Applicable terms will be coded according to the coding conventions for this study. Logical checks will be implemented to ensure data quality and accuracy.

Any necessary changes will be made in the clinical database, and data review and validation procedures will be repeated as needed. Data from external sources will be compared with the information available in the eCRF. Discrepancies found will be queried.

At the conclusion of the study, the eCRF and all other study data will be locked to further additions or corrections. Locking the study data represents the acknowledgement that all data have been captured and confirmed as accurate.

10.3.1 Archiving of data and data retention

The archiving period of study-related data (see essential documents, Section 8 of the Note for Guidance on Good Clinical Practice) is based on the requirements of the

"Allgemeine Verwaltungsvorschrift zur Anwendung der Arzneimittelprüfrichtlinien" and the Note for Guidance on Good Clinical Practice.

According to this, sponsor must arrange for essential clinical study documents (including case report forms) other than subject's medical files, to be kept by the owners of the data (e.g., sponsor, investigator).

Subject's medical files should be retained in accordance with applicable legislation and in accordance with the maximum period of time permitted by the clinic, institution or private practice.

The investigator/institution should take measures to prevent accidental or premature destruction of these documents.

The sponsor can require documents to be retained for a longer period of time. It is the responsibility of the sponsor to inform the clinic, institution (CRS) or practice in writing as to when these documents no longer need to be retained.

Nevertheless, before destruction of the respective documents (e.g. source data) in addition the managing directors of CRS have to be consulted.

10.3.2 Data access

The sponsor has to ensure that data collected are complete, accurate, reliable and consistent. Data changes should be recorded in a way that there is no possibility to delete or change data without tracking, i.e., the original data should be retrievable. Security systems will be in place to prevent unauthorized access to the data.

Each study site must maintain a list of individuals who are allowed to enter or change data. This list has to be filed in the investigator site file.

11 Quality assurance and quality control, audits, and inspections

11.1 Quality control and quality assurance

The sponsor is responsible for implementing and maintaining quality assurance and quality control systems with written SOPs to ensure that studies are conducted and data are generated, documented (recorded), and reported in compliance with the protocol, GCP, and the applicable regulatory requirements.

The sponsor is responsible for securing agreement from all involved parties to ensure direct access to all study related sites, source data/documents, and reports for the purpose of monitoring and auditing by the sponsor, and inspection by domestic and foreign regulatory authorities [GCP].

Quality control should be applied to each stage of data handling to ensure that all data are reliable and have been processed correctly [GCP].

Agreements, made by the sponsor with the investigator/institution and any other parties involved with the clinical study, should be in writing, as part of the protocol or in a separate agreement [GCP].

11.2 Audit

Study related activities and documents may be audited according to the respective audit plan to determine if performance and evaluation of the study were compliant with the protocol, SOPs, GCP and the applicable regulatory requirements. Audits will be performed by auditors who are independent from the clinical study and its conduct.

The investigator will permit the auditors access to the facilities and documents at agreed times.

11.3 Inspection

Regulatory authorities may conduct an official review of all study documents, facilities, records and other material related to the study.

The investigator must cooperate with any inspection.

The subjects will agree with signing the informed consent to the direct access of their data for study -related monitoring, audit, IEC review, and regulatory inspection.

12 Confidentiality of subject data

All clinical information will be recorded, processed, handled, and stored by the sponsor and investigator in such a way that it can be accurately reported, interpreted and verified while the confidentiality of records and the personal data of the subjects remain protected in accordance with the applicable law on data protection.

All parties being involved in recording and processing of study related personal data will implement appropriate technical and organizational measures to protect information and personal data against unauthorized or unlawful access, disclosure, dissemination, alteration, or destruction or accidental loss, in particular where the processing involves transmission over a network.

Where processing of personal data is carried out on behalf of the sponsor, the sponsor will use only processors providing sufficient guarantees to implement appropriate technical and organizational measures in such a manner that processing will meet the requirements of the applicable European law on data protection. This will be governed by a written contract.

All study related records and data of the subjects which will be transferred to the sponsor or regulatory authorities will be pseudonymized.

The investigator ensures that any documents or data disclosed to the sponsor do not contain any information disclosing the identity of the subject.

13 Reporting

A clinical study report according to ICH Guideline "Note for Guidance on Structure and Content of Clinical Study Reports" will be issued.

A summary of the study results will be provided to the IEC and the regulatory authority within one year after end of study.

14 Publication policy

In case the sponsor intends a publication of the study results, manuscripts thereon shall be discussed by the investigator and the sponsor’s representative(s) prior to publication. Regard shall be given to the sponsor's legitimate interests, e.g., containing optimal patent protection, coordination of submissions to health authorities or with other ongoing studies in the same therapeutic field, protection of confidential data, and information, etc.

The sponsor's comments shall be given within 30 days after receipt of the publication draft. If there is no consensus, the senior author of the manuscript and the sponsor's representative(s) shall further discuss and mutually agree on the final wording or disposition of the publication.

The above described procedure also applies to information on prematurely discontinued and other non-completed studies.

Results from investigations shall not be made available to any third parties by the investigating team outside the publication procedure as set out above.

The sponsor will not quote from publications by investigators in its scientific information and/or promotional material without full acknowledgement of the source (i.e. author and reference).

15 Insurance

The sponsor will maintain clinical study insurance coverage for the subjects participating in this study according to applicable laws and regulation.

The investigator will inform the subjects about the insurance during the informed consent procedure. Terms and conditions of the insurance will be accessible for the subjects on request. The subjects must be informed in writing about their responsibilities and they will receive a copy of the insurance regulations.

16 Responsibilities and finances

Responsibilities and finances will be specified in separate contracts.

17 References

[1] Investigator Brochure of AT1001 (migalastat hydrochloride). Version 17, 09 Aug 2019.[15]

[2] Summary of Product Characteristics Galafold® 123 mg hard capsules, 30 Apr 2018.

[3] Golper TA, Marx MA, Shuler C, Benett WM. Drug dosage in dialysis patients, in Replacement of Renal Function by Dialysis (4th ed), edited by Jacobs C, Kjellstrand CM, Koch CM, Winchester JF. London, Kluwer Academic Publishers. 1996. Pp. 750-820.

[4] Keller F, Wilms H, Schultze G, Offermann G, Molzahn M. Effect of plasma protein binding, volume of distribution and molecular weight on the fraction of drugs eliminated by hemodialysis. Clin Nephrol 1983; 19: 201-205.

[5] Lee CS, Marbury TC. Drug therapy in patients undergoing haemodialysis: Clinical pharmacokinetical considerations. Clin Pharmacokinet 1984; 9:42-66.

[6] Izzedine H, Launay-Vacher V, Baumelou A, Deray G. An appraisal of antiretroviral drugs in hemodialysis. Kidney International 2001; 60: 821-830.

[7] Burch GE. History of precordial leads in electrocardiography. Eur J Cardiol. 1978 Sep; 8 (2): 207-36.

[15] Update of Investigator's Brochure version

18 Amendments

18.1 Amendment 1

Amendment 1 dated 10 Apr 2019 is a non-substantial amendment.

18.1.1 Overview of changes to the study

This amendment is issued to change the following:

Modification	Update	Section
1	Change of address of legal representative in European Union.	Title page<br>Section 6
2	Fax number and email address changed for reporting of serious adverse events.	Section 19.4.2

18.1.2 Changes to the protocol text

18.1.2.1 Title page

This section was changed as a result of Modification 1.

Old text

**Legal representative in the European Union**	Amicus Therapeutics Europe Limited<br>~~70 Sir John Rogerson's Quay~~<br>~~Dublin 2~~<br>~~Ireland D02R296~~

New text

**Legal representative in the European Union**	Amicus Therapeutics Europe Limited<br>Block 1<br>Corporate Park<br>Ballycoolen Road<br>Blanchardstown<br>Dublin<br>D15 AKK1 Ireland

18.1.2.2 Section 6 Investigators and study administrative structure

This section was changed as a result of Modification 1.

Old text

Legal representative in the European Union
Amicus Therapeutics Europe Limited
~~70 Sir John Rogerson's Quay~~
~~Dublin 2~~
~~Ireland D02R296~~

New text

Legal representative in the European Union
Amicus Therapeutics Europe Limited
Block 1
Corporate Park
Ballycoolen Road
Blanchardstown
Dublin
D15 AKK1 Ireland

18.1.2.3 Section 19.4.2 Reporting serious adverse events

This section was changed as a result of Modification 2.

Old text

Serious adverse events must be documented and reported to Amicus within **24 h of any study personnel knowledge of the events**. Serious adverse event reports must be faxed to the designated safety fax number (see below) to ensure appropriate dissemination and processing of the information. An alternate email address is provided as a backup, if the fax transmission is unsuccessful.

SAE FAX number: ~~+1 646 963 2056~~
If the primary method fails, please use email: ~~saereporting_fabry@amicusrx,com~~

New text

Serious adverse events must be documented and reported to Amicus within **24 h of any study personnel knowledge of the events**. Serious adverse event reports must be faxed to the designated safety fax number (see below) to ensure appropriate dissemination and processing of the information. An alternate email address is provided as a backup, if the fax transmission is unsuccessful.

SAE FAX number: + 1-866-422-1278
If the primary method fails, please use email: safetyreporting@amicusrx.com

18.2 Amendment 2

Amendment 2 dated 29 Aug 2019 is a non-substantial amendment.

18.2.1 Overview of changes to the study

This amendment is issued to clarify the following:

Modifi-cation	Update	Section
1	The 2 methods of hemodialysis being used to assess recovery of migalastat in dialysate are clarified:<br>The 2 methods include hemodiafiltration and standard hemodialysis. Both methods are routinely used in clinical practice in the EU and are similar to each other with regard to elimination of small molecules, such as migalastat (163.17 Dalton). Therefore, because of the low molecular weight of migalastat, it is not anticipated that percent of the migalastat dose recovered will significantly differ between the 2 methods.<br>In the final protocol (version 2.0), the term "hemodialysis" is used as the mode of dialysis to be tested in this study without specification to either of the two aforementioned methods. To ensure that the data collected reflect the standard clinical practice, this amendment clarifies the use of hemodiafiltration, in addition to standard hemodialysis, in this study. Since a total of 6 subjects are planned for evaluation, the sample size for each method is planned to be balanced to include 3 subjects on hemodiafiltration and 3 subjects on standard hemodialysis. This amendment has no impact on the safety of the study participants, or on the validity of data.	Section 2<br>Section 8<br>Section 9.1<br>Section 9.2<br>Section 9.3<br>Section 9.3.1.2<br>Section 9.3.4<br>Section 9.7.1.1<br>Section 9.7.1.3<br>Section 9.7.2
2	The protocol was harmonized regarding order of recruitment and dosing as described in Note to File No. 01 dated 19 Jun 2019: Subjects with ESRD and matched subjects with normal renal function will be recruited in parallel but not dosed at the same time. A matched subject with normal renal function will be enrolled after the follow-up visit of his/her matched ESRD subject.	Section 2

**Modification**	**Update**	**Section**
3	In the revision of the Investigator's Brochure adjudication of adverse drug reactions resulted in reduction from 27 preferred terms (Edition 16) to 9 preferred terms (Edition 17).	Section 7.2

18.2.2 Changes to the protocol text

18.2.2.1 Section 2 Synopsis

This section was changed as a result of Modification 1 and 2.

Old table

**[…]**	
**Study objectives:**	Primary objective:<br>• To characterize the pharmacokinetics (PK) of migalastat in non-Fabry end-stage renal disease (ESRD) subjects who are receiving hemodialysis treatment<br>Secondary objective:<br>• To assess the safety and tolerability of migalastat in non-Fabry ESRD subjects who are receiving hemodialysis treatment
**[…]**	
**Study design:**	This study will be conducted as a single center, Phase 1, open-label, and non-randomized design in non-Fabry subjects with ESRD on hemodialysis and matched control subjects with normal renal function.<br>[…]<br>Subjects with normal renal function<br>• Single oral dose of migalastat<br>Recruitment will start with the ESRD subjects. For safety reasons, one ESRD subject will be treated first and the safety data will be discussed between sponsor and investigator. If the safety data for this individual subject is considered acceptable, the other subjects of the group will be treated in parallel.<br>~~After the ESRD subjects have completed the study, subjects with normal renal function will be recruited.~~ These subjects will be matched 1:1 to subjects in the ESRD group based on their age (±10 years), body weight (±10 kg), and sex. In each group at least 2 subjects of each sex will be enrolled.

**[…]**	
**Number of subjects:**	12 subjects are planned (6 ESRD subjects and 6 subjects with normal renal function)
**[…]**	
**Plan for statistical analyses:**	All measured variables and derived PK parameters will be listed individually and, as appropriate, tabulated by descriptive statistics for ~~subjects with ESRD and for subjects with normal renal function~~. Figures will be provided as appropriate.<br>[…]

New table

**[…]**	
**Study objectives:**	Primary objective:<br>• To characterize the pharmacokinetics (PK) of migalastat in non-Fabry end-stage renal disease (ESRD) subjects who are receiving hemodialysis treatment (standard hemodialysis or hemodiafiltration)<br>Secondary objective:<br>• To assess the safety and tolerability of migalastat in non-Fabry ESRD subjects who are receiving hemodialysis treatment (standard hemodialysis or hemodiafiltration)
**[…]**	
**Study design:**	This study will be conducted as a single center, Phase 1, open-label, and non-randomized design in non-Fabry subjects with ESRD on hemodialysis (standard hemodialysis or hemodiafiltration) and matched control subjects with normal renal function.<br>[…]<br>Subjects with normal renal function<br>• Single oral dose of migalastat<br>Recruitment will start with the ESRD subjects. For safety reasons, one ESRD subject will be treated first and the safety data will be discussed between sponsor and investigator. If the safety data for this individual subject is considered acceptable, the other subjects of the group will be treated in parallel.<br>Subjects with ESRD and matched subjects with normal renal function will be recruited in parallel but not dosed at the same time. A matched subject with normal renal function will be enrolled after the follow-up visit of his/her matched ESRD subject. These subjects will

	be matched 1:1 to subjects in the ESRD group based on their age (±10 years), body weight (±10 kg), and sex. In each group at least 2 subjects of each sex will be enrolled.
**[…]**	
**Number of subjects:**	12 subjects are planned (6 ESRD subjects [3 subjects on standard hemodialysis, 3 subjects on hemodiafiltration] and 6 subjects with normal renal function)
**[…]**	
**Plan for statistical analyses:**	All measured variables and derived PK parameters will be listed individually and, as appropriate, tabulated by descriptive statistics for ESRD subjects on standard hemodialysis, ESRD subjects on hemodiafiltration, subjects with normal renal function matched to ESRD subjects on standard hemodialysis, and subjects with normal renal function matched to ESRD subjects on hemodiafiltration. Figures will be provided as appropriate.<br>[…]

18.2.2.2 Section 7.2, Description of the investigational medicinal product

The description of adverse reactions in this section was changed as a result of Modification 3.

Old text:

[…]

Common adverse reactions (≥ 1/100, < 1/10) were: ~~depression, paresthesia, dizziness, hypoesthesia, vertigo, palpitations, dyspnea, epistaxis, diarrhea, nausea, abdominal pain, constipation, dry mouth, defecation urgency, dyspepsia, rash, pruritus, muscle spasms, myalgia, torticollis, pain in extremity, proteinuria, fatigue, pain, blood creatinine phosphokinase increased, and weight increase~~.

[…]

New text:

[…]

Very common adverse reactions (≥ 1/10) were: abdominal pain, diarrhoea, nausea, fatigue, pain, dizziness and headache.

Common adverse reactions (≥ 1/100, < 1/10) were: rash and pruritus.

18.2.2.3 Section 8 Study objectives and endpoints

This section was changed as a result of Modification 1.

Old table

**Objectives**	**Endpoints**	**Comments**
Primary objective<br><br>To characterize the pharmacokinetics (PK) of migalastat in non-Fabry end-stage renal disease (ESRD) subjects who are receiving hemodialysis treatment	Primary endpoints<br><br>[…]	See Section 9.5.2.3
Secondary objective<br><br>To assess the safety and tolerability of migalastat in non-Fabry ESRD subjects who are receiving hemodialysis treatment	Secondary endpoints<br><br>[…]	See Section 9.5.3

New table

**Objectives**	**Endpoints**	**Comments**
Primary objective<br><br>To characterize the pharmacokinetics (PK) of migalastat in non-Fabry end-stage renal disease (ESRD) subjects who are receiving hemodialysis treatment (standard hemodialysis or hemodiafiltration)	Primary endpoints<br><br>[…]	See Section 9.5.2.3
Secondary objective<br><br>To assess the safety and tolerability of migalastat in non-Fabry ESRD subjects who are receiving hemodialysis treatment (standard hemodialysis or hemodiafiltration)	Secondary endpoints<br><br>[…]	See Section 9.5.3

18.2.2.4 Section 9.1 Overall study design and plan description

This section was changed as a result of Modification 1.

Old text

This study will be conducted as a single center, Phase 1, open-label, and non-randomized design in 6 non-Fabry subjects with ESRD on hemodialysis and in 6 matched control subjects with normal renal function.

Renal function of the subjects will be classified by the estimated glomerular filtration rate (eGFR) according to Modification of Diet in Renal Disease (MDRD) equation (see Section 9.5.1.3).

Details are provided in Table 9-1:

Table 9-1: Overview of renal function groups

Renal function	eGFR according to MDRD equation [mL/min/1.73 m^2]	Number of subjects
ESRD on hemodialysis	<15	6
Normal renal function (matched 1:1 to subjects in the ESRD group based on age [±10 years], body weight [±10 kg], and sex; in each group at least 2 subjects of each sex will be enrolled).	≥80	6

eGFR: estimated glomerular filtration rate, ESRD: end-stage renal disease, MDRD: Modification of Diet in Renal Disease

[…]

New text

This study will be conducted as a single center, Phase 1, open-label, and non-randomized design in 6 non-Fabry subjects with ESRD on hemodialysis (3 subjects on standard hemodialysis and 3 subjects on hemodiafiltration) and in 6 matched control subjects with normal renal function. (Please note: In instances within the protocol where standard hemodialysis and hemodiafiltration are not specified, the term "hemodialysis" refers to both..

Renal function of the subjects will be classified by the estimated glomerular filtration rate (eGFR) according to Modification of Diet in Renal Disease (MDRD) equation (see Section 9.5.1.3).

Details are provided in Table 9-1.

Table 9-1: Overview of renal function groups

Renal function	eGFR according to MDRD equation [mL/min/1.73 m^2]	Number of subjects
ESRD on hemodialysis	<15	6

(standard hemodialysis or hemodiafiltration)		(3 on standard hemodialysis, 3 on hemodiafiltration)
Normal renal function (matched 1:1 to subjects in the ESRD group based on age [±10 years], body weight [±10 kg], and sex; in each group at least 2 subjects of each sex will be enrolled).	≥80	6

eGFR: estimated glomerular filtration rate, ESRD: end-stage renal disease, MDRD: Modification of Diet in Renal Disease

[…]

18.2.2.5 Section 9.2 Discussion of study design, including the choice of control groups

This section was changed as a result of Modification 1.

Old text

[…]

Plasma migalastat $t_{1/2}$ increases with worsening renal function (up to 32.3 h in subjects with severe renal impairment). As the study drug is considered to be dialyzable (see Section 7.3), a washout phase of at least 8 days between administrations in subjects on hemodialysis is considered to be sufficient.

Subjects with normal renal function will be matched 1:1 to subjects in the ESRD group based on their age, body weight, and sex (for details, see Section 9.1). In each group at least 2 subjects of each sex will be enrolled.

[…]

New text

[…]

Plasma migalastat $t_{1/2}$ increases with worsening renal function (up to 32.3 h in subjects with severe renal impairment). As the study drug is considered to be dialyzable (see Section 7.3), a washout phase of at least 8 days between administrations in subjects on hemodialysis is considered to be sufficient.

Two methods of hemodialysis are investigated in this study (standard hemodialysis, hemodiafiltration) to ensure that the data collected reflect the standard clinical practice. The 2 methods are similar to each other with regard to elimination of small molecules. Therefore, because of the low molecular weight of migalastat (163.17 Dalton), it is not anticipated that percent of the migalastat dose recovered will significantly differ between the 2 methods.

Subjects with normal renal function will be matched 1:1 to subjects in the ESRD group based on their age, body weight, and sex (for details, see Section 9.1). In each group at least 2 subjects of each sex will be enrolled.

[…]

18.2.2.6 Section 9.3 Selection of study population

This section was changed as a result of Modification 1.

Old text

In total, 6 subjects with ESRD on hemodialysis and 6 matched subjects with normal renal function will participate in the treatment phase of the clinical study.

[…]

New text

In total, 6 subjects with ESRD on hemodialysis (3 subjects on standard hemodialysis, 3 subjects on hemodiafiltration) and 6 matched subjects with normal renal function will participate in the treatment phase of the clinical study.

[…]

18.2.2.7 Section 9.3.1.2 Subjects with ESRD

This section was changed as a result of Modification 1.

Old table

		**Screening**	**Day -1***
14	Subject receives hemodialysis (at least 4 h every 72 h).	X	X

New table

		**Screening**	**Day -1***
14	Subject receives hemodialysis (standard hemodialysis or hemodiafiltration) (at least 4 h every 72 h).	X	X

18.2.2.8 Section 9.3.4 Subject replacement

This section was changed as a result of Modification 1.

Old text

Subjects with ESRD are enrolled until ~~6~~ subjects with both periods valid for PK analyses are available. For each of these subjects a matched healthy control is included.

New text

Subjects with ESRD are enrolled until 3 subjects on standard hemodialysis and 3 subjects on hemodiafiltration with both periods valid for PK analyses are available. For each of these subjects a matched healthy control is included.

18.2.2.9 Section 9.7.1.1 General statistical considerations

This section was changed as a result of Modification 1.

Old text

[…]

All measured variables and derived PK parameters will be listed individually and, if appropriate, tabulated by descriptive statistics for subjects with ESRD and for subjects with normal renal function. Figures will be provided as appropriate.

Descriptive statistics will provide the number of observation and the absolute and relative frequency of categorical variables. For the continuous variables, number of observations, arithmetic mean, standard deviation, coefficient of variation (if appropriate), median as well as minimum and maximum will be given.

[…]

As appropriate, summaries will be grouped for subjects with ESRD, for subjects with normal renal function, and time of assessment.

New text

[…]

All measured variables and derived PK parameters will be listed individually and, if appropriate, tabulated by descriptive statistics for ESRD subjects on standard hemodialysis, ESRD subjects on hemodiafiltration, subjects with normal renal function matched to ESRD subjects on standard hemodialysis, and subjects with normal renal function matched to ESRD subjects on hemodiafiltration. Figures will be provided as appropriate.

For interference statistical analyses (PK dependent on eGFR) data will be pooled for ESRD subjects and for subjects with normal renal function.

Descriptive statistics will provide the number of observation and the absolute and relative frequency of categorical variables. For the continuous variables, number of observations, arithmetic mean, standard deviation, coefficient of variation (if appropriate), median as well as minimum and maximum will be given.

[…]

As appropriate, summaries will be grouped for ESRD subjects on standard hemodialysis, ESRD subjects on hemodiafiltration, subjects with normal renal function matched to ESRD subjects on standard hemodialysis, subjects with normal renal function matched to ESRD subjects on hemodiafiltration, and time of assessment.

18.2.2.10 Section 9.7.1.3 Population characteristics

This section was changed as a result of Modification 1.

Old text

Demographic data

Demographic variables and baseline characteristics will be listed and summarized for ~~subjects with ESRD, for subjects with normal renal function~~, and overall for the safety and the PK population (provided separately if both populations are different). The descriptive statistics will include number of observations, mean, standard deviation, median, minimum and maximum for continuous variables and number of observations and their percentages for categorical parameters.

Consumption history

Data about smoking and alcohol habits, xanthine consumption and drug abuse will be listed and summarized for ~~subjects with ESRD, for subjects with normal renal function~~, and overall both for the safety and the PK population, if different.

[…]

eGFR

Data will be listed and summarized for ~~subjects with ESRD and for subjects with normal renal function~~ both for the safety and the PK population, if different.

New text

Demographic variables and baseline characteristics will be listed and summarized for ESRD subjects on standard hemodialysis, ESRD subjects on hemodiafiltration, subjects with normal renal function matched to ESRD subjects on standard hemodialysis, subjects with normal renal function matched to ESRD subjects on hemodiafiltration, and overall for the safety and the PK population (provided separately if both populations are different). The descriptive statistics will include number of observations, mean, standard deviation, median, minimum and maximum for continuous variables and number of observations and their percentages for categorical parameters.

Consumption history

Data about smoking and alcohol habits, xanthine consumption and drug abuse will be listed and summarized for ESRD subjects on standard hemodialysis, ESRD subjects on hemodiafiltration, subjects with normal renal function matched to ESRD subjects on standard hemodialysis, subjects with normal renal function matched to ESRD subjects on hemodiafiltration, and overall both for the safety and the PK population, if different.

[…]

eGFR

Data will be listed and summarized for ESRD subjects on standard hemodialysis, ESRD subjects on hemodiafiltration, subjects with normal renal function matched to ESRD subjects on standard hemodialysis, and subjects with normal renal function matched to ESRD subjects on hemodiafiltration for the safety and the PK population, if different.

18.2.2.11 Section 9.7.2 Determination of sample size

This section was changed as a result of Modification 1.

Old text

The planned sample size of 6 evaluable subjects per renal function group is not based on a power calculation, but is judged based on experience to be adequate to obtain reliable results meeting the objectives of this study.

New text

The planned sample size of 6 evaluable subjects per renal function group (in the ESRD group on hemodialysis: 3 subjects on standard hemodialysis, 3 subjects on hemodiafiltration) is not based on a power calculation, but is judged based on experience to be adequate to obtain reliable results meeting the objectives of this study.

19 Adverse events and serious adverse events

Investigators and study staff are responsible for detecting, documenting, and reporting AEs and SAEs. For each subject, reporting of AEs and SAEs begins after written informed consent/assent is provided.

19.1 Definitions

19.1.1 Adverse event

An AE is defined as any untoward medical occurrence in a patient or clinical investigation subject administered a pharmaceutical product and which does not necessarily have a causal relationship with this treatment. An AE can therefore be any unfavorable and unintended sign, symptom, or disease temporally associated with the use of a medicinal product, whether or not considered related to the medicinal product.

Therefore, AEs include:

- The onset of new signs, symptoms, conditions, and illnesses
- Exacerbation of pre-existing conditions or illnesses
- Abnormal laboratory findings deemed clinically significant by the investigator except results which are clinically significant due to the underlying disease

- Physical examination changes deemed clinically significant by the investigator
- Abnormal medical evaluation findings (e.g., ECG) that are not documented at the Screening Visit and/or, in the investigator’s opinion, represent a clinically significant change in the subject’s health during study participation
  - Screening medical evaluation findings (e.g., ECG) that were not previously provided as medical history, and can be determined as starting prior to the Screening Visit, are not considered AEs and will be recorded as medical history.

Adverse events will be recorded in the eCRF and subject’s source record beginning from the time written consent/assent is provided through the follow-up visit (7 days after the last dose of study drug).

A single diagnosis should be entered when known. If a clear diagnosis cannot be determined at the time of eCRF and the subject’s source record entry, each sign and symptom must be recorded individually, until a final diagnosis is established. All conditions, signs, or symptoms that are present in the subject's medical history at screening should only be reported as AEs if they worsen (i.e., increase in severity) following the first dose of study drug.

Adverse events that begin or pre-existing conditions that worsen after the first dose of study drug will be considered TEAEs.

19.1.2 Serious adverse events

An SAE is any AE occurring at any dose that results in any of the following outcomes:

- Death
- Is life-threatening
  - Any AE, in the view of either the investigator or Amicus, in which its occurrence places the subject at immediate risk of death. It does not include an AE that, had it occurred in a more serious form, might have caused death.
- Requires inpatient hospitalization or prolongs existing hospitalization
  - Hospitalization signifies the subject has been admitted, regardless of duration, for observation and/or treatment that would not have been appropriate in a physician’s office or outpatient setting.
  - Hospitalizations for elective or pre-planned treatment of a pre-existing condition do not have to be reported as SAEs provided:

    - The condition is documented in the subject's medical history (eCRF and source record) and has not worsened since the informed consent/assent was first signed; and
    - The planned procedure is documented in the subject's source record at the Screening Visit.
  - Emergency room/department or outpatient treatments that do not result in admission do not have to be reported as an SAE, unless another SAE criterion is met.
    - Events assessed and treated in these circumstances should be captured as AEs.
  - Hospitalizations solely based on subject logistics (e.g., subject is admitted due to limited hospital accessibility for what would otherwise be an outpatient procedure) do not have to be reported as SAEs, provided they are clearly defined as such in the subject's source record.
- Persistent or significant incapacity or substantial disruption of the ability to conduct normal life functions
- Congenital anomaly/birth defect

An important medical event that may not result in one of the above serious outcomes may be considered an SAE when, based upon appropriate medical judgment, it may jeopardize the subject and may require medical or surgical intervention to prevent one of the listed serious outcomes.

Examples of such medical events include allergic bronchospasm requiring intensive treatment in an emergency room or at home, blood dyscrasias, or convulsions that do not result in in-patient hospitalization, or development of drug dependency or drug abuse.

If the following 4 elements are known, the event must be reported as described in Section 19.4.2.

- Identifiable subject
- Event term
- Study drug
- Identifiable reporter

Additionally, the investigator's assessment of an event's relationship to study drug (see Section 19.2) is essential for Amicus to appropriately process the report and must be included.

Subjects must be informed and understand that they should report events meeting the definition of serious to study personnel as soon as possible (and to not wait until their next study visit).

If a non-serious event becomes serious, the change in status must be appropriately entered in the eCRF and the subject's source record, and reported to Amicus as described in Section 19.1.2.

If the investigator becomes aware of an SAE that occurs more than 30 days after the last dose of study drug and considers the event possibly, probably, or definitely related to study drug, the investigator should contact the Amicus Medical Monitor to determine how the SAE should be documented and reported (see Section 19.4.2).

19.2 Relationship to study drug

The investigator or a medically qualified sub-investigator will review each event and assess its relationship to the study drug based on available information according to the following guidelines:

- Definite: A reaction that follows a distinct temporal relationship from administration of the study drug; that follows a known reaction to the agent or chemical group of the study drug; and that cannot be explained by the subject's clinical state or other factors.
- Probable: A reaction that follows a reasonable temporal sequence from administration of the study drug; that follows a known or expected response pattern to the suspected study drug; and that could not be reasonably explained by the known characteristics of that subject's clinical state.
- Possible: A reaction that follows a reasonable temporal sequence from administration of the study drug; that follows a known or expected response pattern to the suspected study drug; but that could readily have been produced by a number of other factors.
- Unlikely: A reaction that does not follow a reasonable temporal sequence from administration of the study drug; however, causality from the study drug cannot be ruled out.
- Unrelated: A reaction for which sufficient data exist to indicate that the etiology is unrelated to the study drug.

For the purpose of expedited SAE regulatory reporting obligations (i.e., to regulatory authority and IEC, events assessed by the investigator as definitely, probably, or possibly related to study drug will be considered "related" to study drug (i.e., associated with the use of study drug). Events assessed as unlikely or unrelated will be considered "not related" to study drug (i.e., not associated with the use of study drug).

19.3 Severity assessment

The investigator or a qualified sub-investigator will review each event and use the following definitions for rating intensity:

- Mild: Awareness of sign, symptom or event, but the AE is easily tolerated and does not interfere with daily activity.
- Moderate: Discomfort enough to cause interference with usual activity and may warrant intervention, but the subject is still able to function.
- Severe: Incapacitating with inability to do usual activities or significantly affects clinical status, and requires medical intervention.

It is important to distinguish the difference between events that meet the definition of serious and events that are deemed as severe in intensity. AEs assessed as severe in intensity are not SAEs unless at least 1 of the outcomes in Section 19.1.2 occurs. An AE of any intensity must be reported as an SAE if at least 1 outcome in Section 19.1.2 occurs.

19.4 Reporting events

19.4.1 Reporting adverse events

AEs will be recorded in the eCRF and subject source record beginning from the time written consent/assent is provided through the follow-up visit (7 days after the last dose of study drug). Required information will be detailed in the eCRF Completion Guidelines.

19.4.2 Reporting Serious Adverse Events - amended

Serious adverse events must be documented and reported to Amicus within **24 h of any study personnel knowledge of the events**. Serious adverse event reports must be faxed to the designated safety fax number (see below) to ensure appropriate dissemination and processing of the information. An alternate email address is provided as a backup, if the fax transmission is unsuccessful.

SAE FAX number: + 1-866-422-1278[16]
If the primary method fails, please use email: safetyreporting@amicusrx.com[17]

Serious adverse event forms should be as complete as possible, with all known information at the time. All relevant supporting documentation (e.g., admission and progress notes, results of diagnostic evaluations/procedures/examinations, etc) available at the time of reporting should be included in the fax (or email, if necessary) along with the SAE report form. All supporting documents must be thoroughly reviewed and de-identified in accordance with local data privacy regulations prior to sending to Amicus. The subject's study number must be included

[16] Fax number changed via Amendment 1
[17] Email address changed via Amendment 1

on each page of a fax or noted in the subject line or body of an email. Reporting timelines (as described above) must not be delayed while obtaining or preparing supporting information.

If more than 1 SAE is identified in a subject simultaneously, separate SAE reports should be generated for each event.

Information not available at the time of the initial report (e.g., event end date and outcome, discharge summary, etc.) must be faxed (or emailed, if necessary) within **24 h of any study personnel knowledge of the information**. All supporting documents must be thoroughly reviewed and de-identified in accordance with local data privacy regulations prior to sending to Amicus. The subject's study number must be included on each page of the fax or in the subject line of the email.

Medical history, concomitant medication, and AE information obtained through SAE reporting must also be recorded in the eCRF.

19.4.3 Additional Reporting Requirements for Suspected Unexpected Serious Adverse Reactions

Amicus is responsible for processing suspected unexpected serious adverse reactions (SUSARs). SUSARs are also referred to as alert reports, expedited safety reports, and IND safety reports.

A SUSAR is defined as any SAE that is determined to be associated with the use of study drug and is unexpected (not currently listed in the safety reference information [see migalastat IB[1]] or is not listed at the specificity or severity that has been observed). Amicus will notify all investigators currently conducting migalastat clinical studies of all SUSARs in accordance with applicable regulations. Suspected unexpected serious adverse reactions will be reported to the regulatory authority and IEC according to the rules in effect in each country where study sites are located:

- If the SUSAR is fatal or life-threatening, regulatory authorities and ethics committees will be notified within 7 calendar days after Amicus learns of the event.
- If the SUSAR is not fatal or life-threatening, regulatory authorities and ethics committees will be notified within 15 calendar days after Amicus learns of the event.

These notifications will need to be filed in the site's Study File Notebook and submitted to the site's IEC in accordance with policy.

Safety updates will be provided periodically to the regulatory authority and IEC responsible for the study according to the rules in effect in Germany. These updates will include information on SUSARs and other relevant safety findings.

19.5 Other reporting situations

19.5.1 Pregnancy

Pregnancy information for female subjects and female partners of male subjects participating in the study is collected by Amicus. Pregnancy in and of itself is not regarded as an AE (unless there is a suspicion that study drug may have interfered with the effectiveness of a contraceptive medication).

If a female subject becomes pregnant during the course of the study, or if the female partner of a male subject becomes pregnant during the subject's participation in the study, Amicus must be informed within 5 working days of any study staff knowledge of the pregnancy. If an SAE occurs in conjunction with the pregnancy, the SAE must be reported as described in Section 19.4.2. Amicus will provide pregnancy report forms and instructions to study personnel regarding collection of pregnancy and outcome information (subject to receipt of data privacy release approvals where required under local privacy laws). Pregnancy report forms must be faxed (or emailed, if necessary) to the designated safety fax number (or email address) (see Section 19.4.2), to ensure appropriate dissemination and processing of the information.

19.5.2 Medication errors, including overdose

Medication error refers to any unintended error in the dispensing or administration of a study drug.

Overdose refers to administration of a quantity given per administration or given per time period that is above its maximum recommended dose.

Medication errors, including overdose, should be captured in the source document and entered into the appropriate page of the eCRFs. If a medication error is associated with AEs or SAEs, AE/SAE reporting requirements are to be followed (Section 19.4.1 and Section 19.4.2, respectively).

19.5.3 Reporting of possible study drug product quality defects

Any defect or possible defect associated with the study drug must be reported to Amicus (clinicalcomplaints@amicusrx.com) within 1 working day of any study personnel knowledge of the possible defect. The study drug and packaging components in question, if available, must be segregated and stored in a secure area at the site under the specified storage conditions until it is determined whether or not the study drug and/or packaging is required for investigation of the possible defect. If the possible defect is associated with an SAE, the SAE must be reported as described in Section 19.4.2. The SAE report must include the possible study drug defect complaint.
